# HfO_2_-based memristive synapses with asymmetrically extended p-n heterointerfaces for highly energy-efficient neuromorphic hardware

**DOI:** 10.1126/sciadv.aec2324

**Published:** 2026-03-20

**Authors:** Babak Bakhit, Xiao Xie, Simon M. Fairclough, Atif Jan, Ingemar Persson, Giuliana Di Martino, Bonan Zhu, Caterina Ducati, Quanxi Jia, Bilge Yildiz, Andrew J. Flewitt, Judith L. MacManus-Driscoll

**Affiliations:** ^1^ Department of Materials Science and Metallurgy, University of Cambridge, Cambridge CB3 0FS, UK.; ^2^ Electrical Engineering, University of Cambridge, JJ Thomson Avenue, Cambridge CB3 0FA, UK.; ^3^ Thin Film Physics Division, Department of Physics (IFM), Linköping University, Linköping 58183, Sweden.; ^4^ School of Aerospace Engineering, Beijing Institute of Technology, Beijing 100081, China.; ^5^ Centre for Analysis and Synthesis, Lund University, Lund 22100, Sweden.; ^6^ Department of Materials Design and Innovation, University at Buffalo, 136 Bell Hall, Buffalo, NY 14260, USA.; ^7^ Department of Nuclear Science and Engineering, Massachusetts Institute of Technology, 77 Massachusetts Avenue, Cambridge, MA 02139, USA.; ^8^ Department of Materials Science and Engineering, Massachusetts Institute of Technology, 77 Massachusetts Avenue, Cambridge, MA 02139, USA.

## Abstract

The escalating energy consumption of existing artificial intelligence hardware has become a serious global issue that demands immediate action. Neuromorphic computing offers promises to drastically reduce this footprint. Here, we introduce multicomponent p-type Hf(Sr,Ti)O_2_ thin films for energy-efficient, resistive switching–based neuromorphic devices. We demonstrate interfacial memristors with ultralow switching currents (≤~10^−8^ A), exceptional cycle-to-cycle and device-to-device uniformities, and retention >10^5^ s. They reveal hundreds of ultralow conductance levels with a modulation range of >50 (without reaching any saturation) and reproducibly satisfy unsupervised learning rules. This performance originates from incorporating a self-assembled p-n heterointerface between p-type Hf(Sr,Ti)O_2_ and n-type TiO_x_N_y_, resulting in a fully depleted space-charge layer asymmetrically extended into Hf(Sr,Ti)O_2_, a large built-in potential, and extremely low saturation current density under reverse bias. Ultralow conductance modulation is controlled by tuning p-n heterointerface’s energy-barrier height through electro-ionic charge migration. This materials-engineering strategy addresses energy consumption and variability in existing memristors, opening a pathway toward energy-efficient neuromorphic computing systems.

## Teaser

Memristive p-n heterointerfaces enable ultralow-energy, highly stable artificial synapses for future neuromorphic hardware.

## INTRODUCTION

Artificial intelligence (AI) is arguably the defining technology of the century that can provide enormous advantages in multiple sectors (*1*–*5*). However, despite the remarkable advances in developing AI algorithms (*6*), the hardware needed for such intensive data processing and storage faces fundamental challenges (*2*–*5*, *7*–*9*). The serious issue of the current AI hardware is the enormous energy consumption (*3*, *7*, *10*, *11*), accelerating with an annual increase rate of ~30% (*12*). This challenge primarily arises from the physical separation of computation and storage units in conventional “Von Neumann architecture” computers that uses substantial energy and time in the frequent data movements between these two units, especially for big data (*13*–*15*). This massive energy consumption, which is alarmingly increasing by ever-growing AI adoption, has already become one of the main global issues with a substantial impact on climate change that urgently demands immediate actions (*16*–*18*). Moreover, training AI models requires energy-efficient data centers with sufficiently high capacities to store enormous amount of digital information, which cannot be achieved by common memory technologies (*8*, *11*, *19*). In this context, neuromorphic compute-in-memory architectures, in which the computing unit is designed within the memory block, are at the core of attention for the next generation of sustainable and high-precision AI hardware (*4*, *7*, *20*–*23*).

The human brain is the best model of a robust biodevice that energy-efficiently processes and stores complex signals in a dense network of neurons and synapses (tiny gaps between the neurons). The neurons communicate through transmitting electrical pulses (also known as spikes), which are converted into chemical signals, across the synapses. These electrochemical processes gradually change synaptic weights and subsequently trigger various actions such as learning, memorizing, recognizing, decision-making, etc. For example, storing information takes place in the form of synaptic weights, or synaptic plasticity is the underlying mechanism for knowledge-based learning (*24*–*26*). Neuromorphic electronic devices emulate the brain’s multilevel (analog) pre- and postacquisitions through a network of programmable processing units that behave like neurons. These bioinspired technologies eliminate the energy-intensive, time-consuming data movement between the compute and memory units (*4*, *13*), resulting in saving >70% of current computing power consumption (*27*), and can be trained to learn and effectively solve problems (*4*, *21*, *28*).

Current AI hardware relies mainly on transistor-based integrated circuits, and a much larger number of smaller transistors are needed as AI grows (*8*, *29*). However, these three-terminal semiconductor-based devices are reaching fundamental quantum mechanical and fabrication limitations (*1*). Unlike transistors, two-terminal nonvolatile memory devices, with simpler capacitor-like structures and fundamentally different materials and device physics, are serious contenders for neuromorphic AI hardware (*1*, *30*–*33*). Among various emerging two-terminal nonvolatile memories, memristors are gaining considerable attention for neuromorphic technologies (*8*, *22*, *34*). However, despite their great promises for a broad range of high-throughput memory and neuromorphic applications, they still have major challenges. In particular, those based on resistively switchable binary oxides, like widely used HfO_2_ (*35*), typically rely on filamentary resistive switching and usually need initial electroforming processes and current compliances to avoid device hard breakdown (*36*, *37*). Such devices suffer from device-to-device and cycle-to-cycle variations, mainly due to filaments’ stochastic nature (*38*–*41*). This critical issue substantially limits the computational accuracy and device performance. The other major problems are their typically restricted numbers of distinguishable conductance levels and difficulties to control them. In filamentary devices, the conductance levels are directly tuned by varying the current compliance (*42*), which is inherently limited, costs further energy, and needs extra special devices adding more design complexities (*43*). Further, these devices usually require high electroforming voltages to create the conductive filaments, and low-resistance states (LRS) (ON states) typically occur at high currents, leading to high power usage that is unfavorable for energy-efficient neuromorphic hardware (*4*, *23*).

In this research, we introduce p-type Hf(Sr,Ti)O_2_ as a class of multicomponent HfO_2_-based switching oxides and propose a two-step, thin-film growth strategy that enables fabricating ultralow-current, analog memristors. The devices show interfacial nonvolatile resistive switching with outstanding uniformity and high retention. They also reveal hundreds of stable and replicable conductance levels (ranging from ~2.5 × 10^−9^ to ~1.4 × 10^−7^ S) with a conductance-modulation range of >50 (without reaching any saturation). These were realized by applying identical 1.0-V electrical spikes, comparable to brain-like signaling. Excellent operational synaptic stability of these Hf(Sr,Ti)O_2_-based devices, essential for spiking neural networks and AI hardware, is confirmed by applying ~40,000 electronic spikes. Essential for the bioinspired synaptic electronic systems, these devices also demonstrate a reproducible emulation of key neural learning rules such as short-term synaptic plasticity and spike timing–dependent plasticity (STDP). Our devices successfully overcome the energy-consumption and variability challenges of current memristors while meeting the essential requirements for energy-efficient neuromorphic technologies.

## RESULTS

### Memristive and neuromorphic characteristics

Multicomponent Hf(Sr,Ti)O_2_ thin films were sputter-deposited on TiN/a-SiO_2_/Si following a two-step growth approach: Approximately 15-nm-thick layers were firstly cosputtered from HfO_2_ and SrTiO_3_ targets in a nonreactive atmosphere [$P_{O_{2}}$ = 0 SCCM (standard cubic centimeter per minute)], and then oxygen with a flow rate of $P_{O_{2}}$ = 20 SCCM was immediately added to the chamber resulting in ~1-nm-thick layers while keeping other deposition parameters constant.

**Fig. 1.**
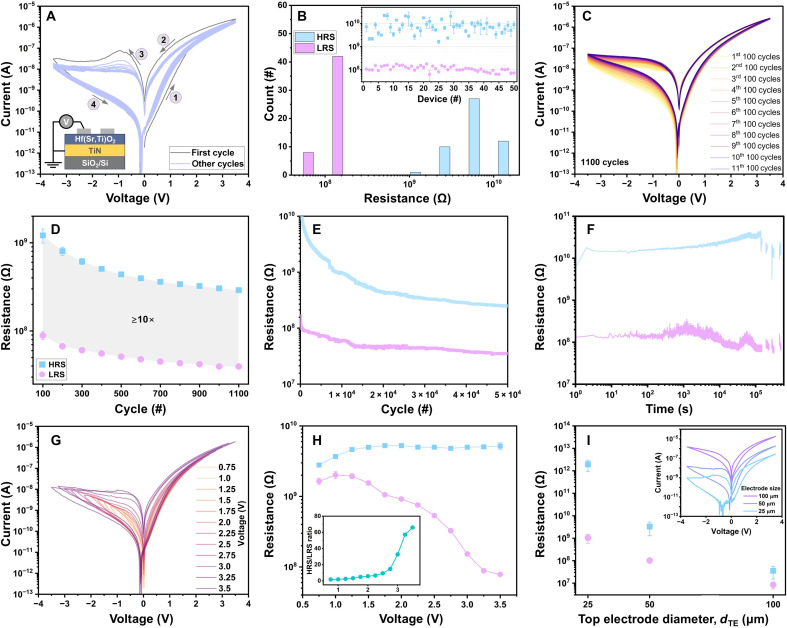
Memristive characteristics. Electrical measurements were carried out by applying a voltage to the Mo top electrodes, while the TiN bottom electrode was grounded. All pristine devices have HRS. (**A**) Resistive switching characteristics (first 30 *I-V* sweeping cycles) of a typical Mo/Hf(Sr,Ti)O_2_/TiN/a-SiO_2_/Si device. The inset in (A) schematically illustrates the cross-sectional device stack and electrical measurement geometry. (**B**) LRS and HRS distribution data of 50 devices obtained at −0.5 V. Error bars in the inset in (B) represent the average values of LRS and HRS determined from 50 *I-V* cycles. (**C**) Endurance data determined by collecting 1100 consecutive *I-V* sweeping cycles. (**D**) LRS and HRS values obtained from the endurance data in (C). (**E**) LRS and HRS data determined from pulsed endurance measurements by applying ±3.5 V pulses. (**F**) LRS and HRS retention data after applying preset pulses of ±3.5 V. (**G**) *I-V* curves for peak voltages ranging from ±0.75 to ±3.5 V with increments of ±0.25 V. (**H**) LRS and HRS values obtained from the *I-V* curves in (G). Error bars in (H) represent the average values of LRS and HRS obtained from 50 *I-V* cycles for each applied voltage. The inset in (H) shows the corresponding HRS/LRS ratios. The data in (A) to (H) are determined from devices with the Mo *d*_TE_ = 50 μm. (**I**) LRS and HRS values obtained from devices with *d*_TE_ = 25, 50, and 100 μm at −0.2 V. These data were collected from 50 different devices for each *d*_TE_.

Figure 1 summarizes the memristive characteristics of Mo/Hf(Sr,Ti)O_2_/TiN devices. Figure 1A shows the first 30 current-voltage (*I*-*V*) curves of a typical device. The cross-sectional device stack and electrical measurement geometry are schematically illustrated in the inset of Fig. 1A. The pristine device has a high-resistance state (HRS). The device resistance exponentially decreases by applying a positive voltage to the Mo top electrode, resulting in switching the device to its LRS (SET process) without needing an initial electroforming process and current compliance. Reversing the voltage polarity then switches the device to its HRS (RESET process). After acquiring the first *I*-*V* curve, the HRS/LRS ratio (memory window) slightly decreases for both voltage polarities, but it remains highly stable for the other *I*-*V* cycles. The *I*-*V* curves do not exhibit the abrupt current increase (characteristic of filamentary devices); instead, they display the gradual, continuous behavior indicative of interface-type switching (*30*). Figure 1B compares the LRS and HRS distributions of 50 different devices. For each device, the LRS and HRS data points represent the average values obtained from 50 *I*-*V* cycles. All devices show highly stable hysteretic *I*-*V* curves that prove exceptional cycle-to-cycle uniformity, with error bars in the inset to Fig. 1B. The resistance state distributions also indicate excellent device-to-device uniformity with memory windows ≥10.

Two different measurement strategies were used to assess the cyclic endurance of these devices: (i) collecting 1100 consecutive *I*-*V* curves and (ii) acquiring LRS and HRS data for >10^4^ pulse-switching cycles. Figure 1C exhibits 1100 consecutive *I*-*V* cycles from a representative device. The *I*-*V* curves at positive voltages show remarkable cycle-to-cycle uniformity, while both LRS and HRS simultaneously undergo slight decreases at negative voltages. However, the memory window does not notably change and remains ≥10 (Fig. 1D). The small drift, which is almost negligible at voltages ≤−0.5 V, stabilizes after ~500 cycles, indicating that it is a slight, transient effect rather than progressive degradation. This behavior is consistent with the field-driven redistribution of mobile ionic species and gradual reconfiguration of interfacial electronic trap states. These processes can induce modest adjustments in the local band-bending, barrier height, and interface stoichiometry during early cycling that cause measurable but self-limiting changes in resistance as the defect population evolves toward a quasi-steady distribution (*44*, *45*). These devices operate through the modulation of the interfacial energy barrier rather than through the formation and rupture of conductive filaments; thus, the drift is unlikely to arise from structural damage. Instead, it likely originates from the minor adjustments of interfacial charge and defect configurations under repeated bias. After this short settling period, both resistance states remain almost stable over subsequent cycles. The endurance data obtained from the pulse-switching measurement (Fig. 1E) indicate robust, stable resistance states up to 5 × 10^4^ switching cycles.

Figure 1F shows the binary-state retention characteristics measured after applying preset pulses of ±3.5 V and reading the resistance every 1 s. Both LRS and HRS remain highly stable for up to 5 × 10^5^ s, with a memory window of >10. The interface switching typically depends on the distribution of many trapped charges and defects rather than on a single mobile species (*44*). Once the charge/defect profile is established by the programming pulses, a quasi-equilibrium space-charge configuration can become energetically trapped (stabilized by multiple defects bound at interface or subinterface sites), making the depletion profile thermally robust even in the presence of limited microscopic ionic mobility (*46*). This field-driven defect redistribution followed by thermally slow relaxation is consistent with the short settling period (~500 cycles) observed in Fig. 1C and the subsequent long-term stability. Similar behavior was reported in interfacial memristors exhibiting analog switching and long retention when deep traps or high local binding energies are present (*47*–*49*). To quantify this long retention, we estimated the thermal lifetime using an Arrhenius relation, $\tau =\tau_{0}\text{exp}\left(E_{a}/k_{B}T\right)$. The activation energies of *E*_a_ = ~0.9 to 1.3 eV (assuming τ_0_ values in the range of 10^−13^ to 10^−9^ s) yield τ ≥ 10^5^ s at *T* = ~25°C. Reported trap depths and vacancy binding/migration energies in HfO_2_-based systems typically fall within or exceed this range (*50*–*54*), making the observed retention quantitatively consistent with the expected thermal stability of deep interface states and bound oxygen-vacancy complexes in HfO_2_-based systems.

To evaluate the effect of atmospheric exposure, a set of devices was stored under standard laboratory ambient conditions (40 to 60% humidity, 20° to 23°C) for 90 days. Electrical measurements performed after storage show no measurable degradation in switching behavior compared with devices stored in the dry cabinet. These results demonstrate the stable electrical performance of our devices in atmospheric exposure, consistent with the chemical robustness of HfO_2_-based systems.

Figure 1G exhibits the *I*-*V* curves of a typical device acquired at different SET-RESET voltages. Several distinct resistance levels are evident in these *I*-*V* curves. The corresponding LRS and HRS values plotted in Fig. 1H show a gradual increase in the memory window from ~2.0 for ±0.75 V to ~15.0 for ±2.75 V and then a rapid increase to ~63.0 for ±3.5 V. The stability of these data was further evaluated by recording 50 *I*-*V* cycles for each SET-RESET voltage, confirming an excellent cycle-to-cycle uniformity for each sweeping voltage. Figure 1I compares the average values of LRS and HRS as a function of Mo top-electrode diameter (*d*_TE_). Both resistance states increase by decreasing *d*_TE_, but this upward trend is more notable for HRS compared to LRS. This is typically considered as a reliable indication of interfacial resistive switching. Overall, the Mo/Hf(Sr,Ti)O_2_/TiN devices exhibit robust memristive characteristics required for advanced data storage technologies.

**Fig. 2.**
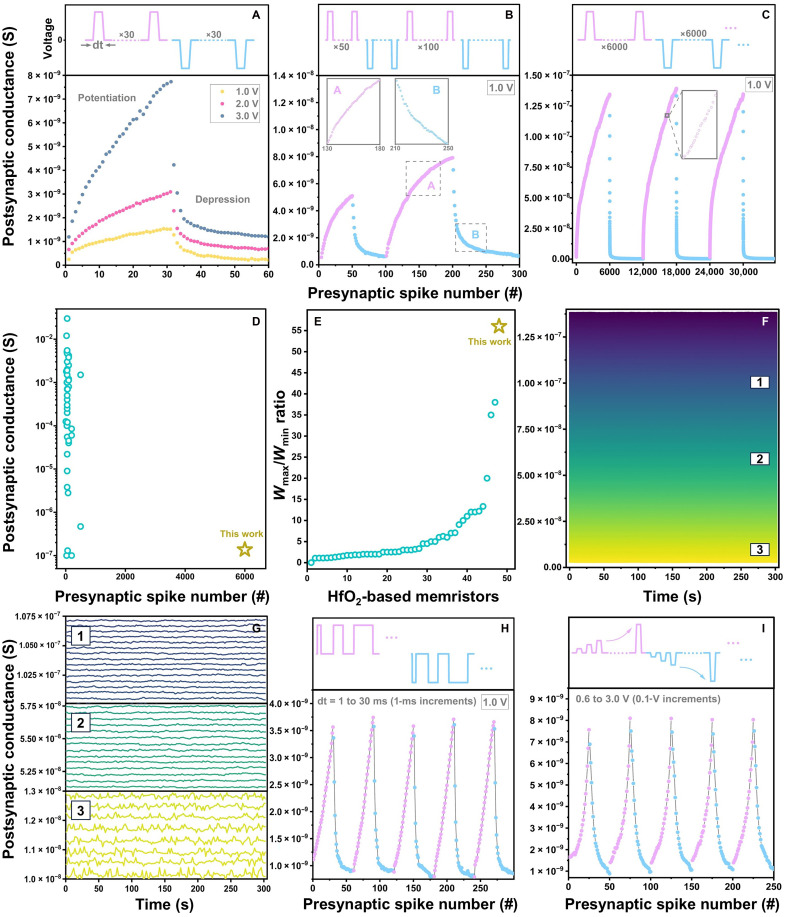
Neuromorphic characteristics (synaptic efficacy). Long-term potentiation and depression as a function of the number of presynaptic spikes with various programming schemes: (**A**) identical spike scheme; spikes with amplitudes of 1.0, 2.0, and 3.0 V, (**B**) and (**C**) identical spike scheme; 1.0-V spikes with various spike numbers (time intervals dt = 1 ms). The presynaptic spikes of each scheme are schematically illustrated above each panel. The remarkable reproducibility of the 6000-spike training scheme was also confirmed for several different devices, agreeing with their memory-based device-to-device uniformity. (**D**) Conductance numbers and values of this work compared to ~50 reported HfO_2_-based memristors (further details given in table S1) and (**E**) their *W*_max_/*W*_min_ ratios (analog window). (**F** and **G**) Multilevel retention data obtained by implementing 1.0-V identical spikes. Each level was obtained after implementing 20 identical spikes. Training the devices with fewer spikes could cause the retention data to overlap, mainly due to noise levels present in ultralow currents. Long-term potentiation and depression data obtained by using (**H**) identical spike scheme; 1.0-V spikes with dt increasing from 1 to 30 ms in 1-ms increments, and (**I**) nonidentical spike scheme; spikes with amplitudes changing from ±0.6 to ±3.0 V in ±0.1-V increments (dt = 1 ms).

**Fig. 3.**
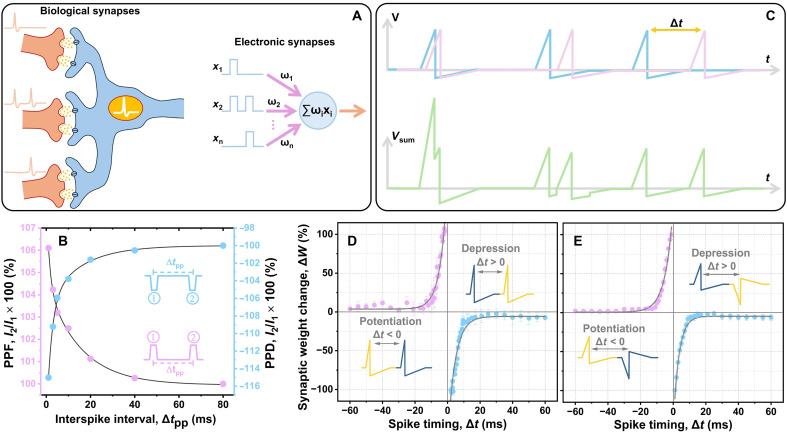
Neuromorphic characteristics (synaptic plasticity). (**A**) Simplified schematic illustrations of biological and electronic synapses. (**B**) PPF and PPD ratios as a function of interspike time intervals Δ*t*_pp_ between two successive |1.0|-V presynaptic spikes. The maximum SD obtained from the PPF and PPD data is < ~2%. (**C**) Schematic plots of neuromorphic voltage profiles used for realizing STDP effects via applying paired spikes with similar polarities. The lower profile is constructed by adding the synaptic voltage profiles shown in the upper plot. Relative synaptic weight change Δ*W* as a function of time difference between presynaptic and postsynaptic spikes Δt realized by implementing triangle-shaped |1.0|-V spikes: paired spikes with (**D**) similar and (**E**) opposite polarities. The solid black curves show exponential fits.

Figures 2 and 3 show the key neuromorphic characteristics of Mo/Hf(Sr,Ti)O_2_/TiN devices. Similar to biological neurons, it is essential for synaptic electronic devices to mimic both memory and learning functionalities (*1*), with satisfying at least two requirements: synaptic efficacy and plasticity (*7*, *55*). Synaptic efficacy refers to the ability of bioinspired devices to alter stored synaptic weights (*W*) (conductance levels) in step-wise fashions upon applying presynaptic spikes, while synaptic plasticity is the device’s ability to modulate *W* by implementing a particular learning rule (*7*). In general, these devices need to have many distinct synaptic weights, ideally varying in linear and symmetrical manners (*11*, *55*, *56*).

Typical long-term potentiation and depression (synaptic efficacy) of the Hf(Sr,Ti)O_2_-based devices, realized by implementing various spike programming schemes, are plotted in Fig. 2. In all schemes, applying positive spikes increases the device postsynaptic conductance levels (potentiation), while the conductance decreases by negative-spike application (depression). These synaptic characteristics directly depend on the amplitudes and time intervals of spikes. Figure 2A exhibits the synaptic weights realized from identical training schemes with spike amplitudes of 1.0, 2.0, and 3.0 V. Incremental and decremental weight changes are distinct with proper precision but not a perfect symmetry. The potentiation nonlinearity coefficients (φ) determined by applying identical 1.0-, 2.0-, and 3.0-V spikes are φ = 0.11, 0.08, and 0.06, respectively, which are close to the ideal value φ = 0; see fig. S1.

The operational capability of these artificial synapses was further assessed by implementing an identical programming scheme with 1.0-V spikes for which the total number of positive and negative spikes increased from 100 to 12,000; see Fig. 2 (B and C) and fig. S2. For all trainings, the conductance steps steadily change with proper separations and excellent replicability, without reaching any saturation. There is a sufficiently high linearity in the conductance modulation obtained from the 6000-spike scheme after applying ~500 spikes with excellent cycle-to-cycle uniformity. Table S1 summarizes the detailed neuromorphic characteristics of our Mo/Hf(Sr,Ti)O_2_/TiN devices with ~50 different HfO_2_-based memristors (showing filamentary, interface, and hybrid resistive switching). Figure 2 (D and E) compares the conductance numbers and values of all these works and their conductance-modulation dynamic ranges (*W*_max_/*W*_min_ ratios). All data points in Fig. 2 (D and E) were extracted entirely from the potentiation measurements of HfO_2_-based memristors rather than from the number of retained, distinguishable conductance levels. We report a continuous, analog tunability over 6000 applied spikes ranging from ~2.5 × 10^−9^ to ~1.4 × 10^−7^ S with *W*_max_/*W*_min_ ratio > 50. This analog number gives the conductance evolution during repeated spiking (analog programming resolution) rather than the number of stable synaptic states. The short-to-intermediate term stability of the conductance modulation is confirmed for a subset of intermediate levels on the order of hundreds of seconds (neuromorphic timescales required for typical online training and short-term learning tasks) in Fig. 2 (F and G) and fig. S2. These retention characteristics demonstrate that the underlying interfacial mechanism produces stable, distinguishable states suitable for neuromorphic operation.

To further improve the conductance-modulation symmetry and linearity, we optimized the programming schemes by implementing (i) identical 1.0-V spikes with different time intervals (dt) (Fig. 2H) and (ii) nonidentical spikes with different amplitudes (Fig. 2I). The results prove the enhanced symmetric and linear synaptic efficacy. The outstanding operational synaptic stability of the Hf(Sr,Ti)O_2_-based devices is also confirmed in fig. S3 by using identical and nonidentical training schemes with applying ~40,000 spikes.

The synaptic update energy was roughly estimated using E=(V×I×t). On the basis of the measured switching currents (~5 × 10^−9^ to ~9 × 10^−11^ A) at *V* = −0.5 V and *t* = 1 ms (typical pulse width used in this work), the synaptic energy falls in the range of ~2.5 pJ to ~45 fJ, which is comparable to or lower than values reported for energy-efficient neuromorphic hardware (*57*, *58*). Together with the uniform, nonfilamentary conductance modulation and the large analog window (*W*_max_/*W*_min_ ratio > ~50), these observations suggest that our memristors have promising system-level potential, although a full network-level hardware demonstration is left for future work.

Figure 3A schematically illustrates simplified biological and electronic synapses. Figure 3B shows paired-pulse facilitation and depression (PPF and PPD) ratios, triggered by applying a pair of |1.0|-V spikes, as a function of interspike time intervals Δ*t*_pp_. Such short-term synaptic plasticity is essential for decoding temporal information in visual and auditory signals. PPF occurs when the second synaptic response is stronger than the first one (*I*_2_ > *I*_1_), while PPD happens when the second synaptic response is weaker than the first response (*I*_2_ < *I*_1_). Positive spikes generate PPF, whereas negative spikes produce PPD. The PPF and PPD ratios are *I*_2_/*I*_1_ × 100%, where *I*_1_ and *I*_2_ are the maximum postsynaptic currents of first and second spikes, respectively. While the PPF ratio decreases by ~6% with increasing Δ*t*_pp_, the absolute value of the PPD ratio follows a decrease of ~15%. In the PPF measurements, for Δ*t*_pp_ shorter than the relaxation time (τ) of mobile charge carriers triggered by the first spike, a fraction of these carriers does not gain adequate time to recombine and diffuse back to their equilibrium positions. Hence, the postsynaptic current is facilitated upon applying the second spike. An opposite manner can be expected for the PPD responses. The best fits for these experimental data have a double-exponential decay function that perfectly matches the short-term biological synaptic plasticity, which consists of two distinct phases: (i) a rapid phase that lasts for a few milliseconds and (ii) a slow phase with a longer millisecond duration (*59*)

$$\text{PPF}\left(\text{PPD}\right)\;\left(\%\right)=Y+A_{1}\cdot \text{exp}\left(-\frac{{\Delta t}_{\text{pp}}}{\tau_{1}}\right)+A_{2}\cdot \text{exp}\left(-\frac{{\Delta t}_{\text{pp}}}{\tau_{2}}\right)$$ (1)

where *A* and τ are the initial facilitation(depression) magnitude and relaxation time for each phase, respectively. For PPF, *Y* = 100%, *A*_1_ = 3.8%, *A*_2_ = 4.6%, τ_1_ = 1.4 ms, and τ_2_ = 15.4 ms, while for PPD, *Y* = −100%, *A*_1_ = −13.8%, *A*_2_ = −6.7%, τ_1_ = 2.2 ms, and τ_2_ = 14.9 ms. Having τ_1_ ≫ τ_2_ demonstrates the presence of both rapid and slow phases in the Hf(Sr,Ti)O_2_-based artificial synapses, in agreement with the biological synapse responses.

STDP is a key unsupervised learning rule in neuromorphic devices for time-dependent functionalities such as speech recognition and image detection (*7*), in which the synaptic weights are modulated as a function of the time difference between presynaptic and postsynaptic spikes Δ*t* (*55*). Figure 3C schematically illustrates neuromorphic voltage plots used to realize STDP effects via applying paired spikes with similar polarities. Figure 3 (D and E) shows relative STDP weight changes Δ*W* realized by implementing two different conventional programming schemes with triangle-shaped |1.0|-V spikes: paired spikes with similar and opposite polarities. The Hf(Sr,Ti)O_2_-based artificial synapse is initially activated upon firing the first spike. While its conductance gradually decreases after the first stimulation, the second spike is applied at a certain Δ*t* to strengthen or weaken the device conductance, depending on the training spike state. Thus, the largest relative weight changes |Δ*W*| occur at the smallest |Δ*t*| values, and |Δ*W*| substantially decreases by increasing |Δ*t*|. Here, both training schemes result in asymmetric, anti-Hebbian STDP effects. Potentiation is achieved by firing postsynaptic spikes before presynaptic ones (Δ*t* < 0), while depression is generated by applying postsynaptic spikes after presynaptic spikes (Δ*t* > 0), schematically illustrated in Fig. 3 (D and E). Similar to STDP in biological neural networks, the artificial synaptic weight-change data can be fit well with the following equation (*60*)

∆W(∆t) (%)={A+exp(−∣∆t∣τ+), ∆T>0A−exp(−∣∆t∣τ−), ∆T<0 (2)

where *A*^±^ and τ^±^ are the exponential-function scaling factors and time constants, respectively. For the paired-spike scheme with similar polarities (Fig. 3B), *A*^±^ values are −158.2 and +167.6, and τ^±^ values are 4.6 and −4.3, while *A*^±^ values are −141.7 and +134.3, and τ^±^ values are 3.4 and −4.9 for the paired-spike scheme with opposite polarities (Fig. 3E). The replicability of these STDP data was also confirmed from different other devices (fig. S4), demonstrating that the Hf(Sr,Ti)O_2_-based devices can effectively emulate the spatiotemporal biological STDP learning rules within millisecond-scale learning windows.

### Materials characterizations

Rutherford backscattering spectrometry (RBS) analyses in fig. S5 indicate that the multicomponent dioxide sputter–deposited following the two-step growth strategy is stoichiometric (O/metal ratio = ~2.0), and the TiN bottom electrode is oxidized, forming a thick TiO_x_N_y_ layer between Hf(Sr,Ti)O_2_ and TiN. The corresponding elemental-concentration depth profile reveals that N in TiO_x_N_y_ increases from ~2.0 atomic % (at %) at close to the Hf(Sr,Ti)O_2_/TiO_x_N_y_ interface to ~46.5 at % at the TiO_x_N_y_/TiN interface, while O decreases from ~65.0 at % to ~5.0 at %. This implies the formation of O- and N-gradient TiO_x_N_y_ layer between TiN and Hf(Sr,Ti)O_2_.

**Fig. 4.**
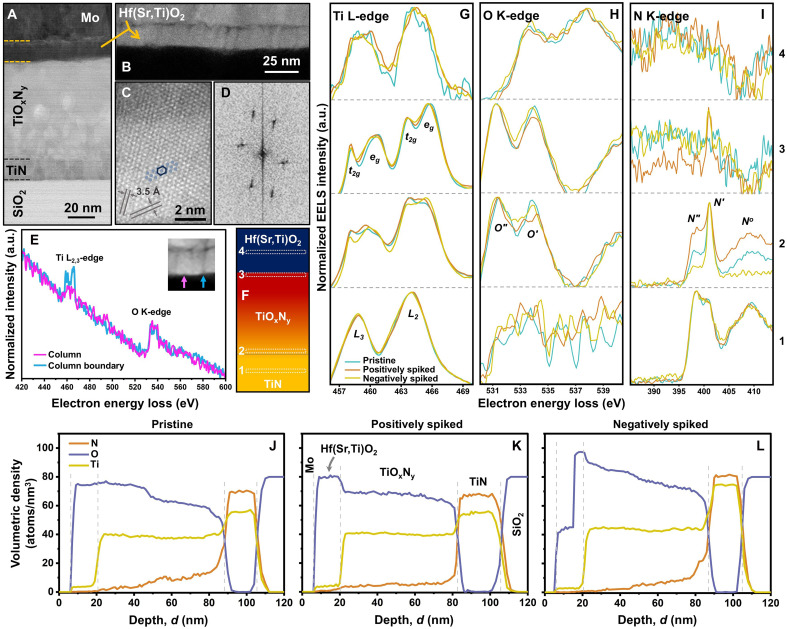
Materials characterization using TEM. Cross-sectional (**A**) ABF-STEM image acquired from the pristine Mo/Hf(Sr,Ti)O_2_/TiN/a-SiO_2_/Si device and (**B**) HAADF-STEM image from Hf(Sr,Ti)O_2_. While TiN preserves its dense columnar microstructure, the TiO_x_N_y_ layer consists of enlarged equiaxed crystallites. (**C**) High-resolution HAADF-STEM image from inside a Hf(Sr,Ti)O_2_ column with (**D**) its corresponding fast Fourier transform image that has reciprocal reflections with a hexagon-shape pattern. (**E**) Ti-L_2,3_ and O-K EELS spectra acquired from inside a bright column and a dark column boundary, indicated by arrows in the inset HAADF-STEM image. The O-K signals from both regions have comparable intensities, confirming that oxygen is uniformly distributed in these regions. However, the intensity of the Ti-L_2,3_ signal acquired from the column boundary is almost doubled compared to inside the column, demonstrating that the dark column boundaries are Ti-rich. (**F**) Schematic cross-sectional illustration of Hf(Sr,Ti)O_2_/TiO_x_N_y_/TiN indicating regions where (**G**) Ti-L_2,3_, (**H**) O-K, and (**I**) N-K EELS spectra were acquired from, at pristine and spiked states. Absolute Ti, O, and N volumetric-density depth profiles of (**J**) pristine, (**K**) positively, and (**L**) negatively spiked devices as a function of depth *d*. a.u., arbitrary units.

Figure 4 summarizes the cross-sectional electron microscopy results of a Mo/Hf(Sr,Ti)O_2_/TiN device. The cross-sectional annular bright-field–scanning transmission electron microscopy (ABF-STEM) image in Fig. 4A exhibits an ~16-nm-thick Hf(Sr,Ti)O_2_ layer and confirms the presence of TiO_x_N_y_, agreeing with the RBS data. The Z-contrast high-angle annular dark-field (HAADF)–STEM image acquired from Hf(Sr,Ti)O_2_ reveals the formation of a columnar microstructure, comprising ~7-nm-wide crystalline nanocolumns that are aligned along the vertical growth direction and have ~3.5-Å plane spacings (see Fig. 4, B to D), agreeing with x-ray diffraction (XRD) data in fig. S6. Figure 4B shows a contrast difference between columns and column boundaries. While the columns are bright, the column boundaries appear dark. These dark atomic-scale thin areas can be related to the formation of low-Z (here, Ti and O) rich regions. Comparing Ti-L_2,3_ and O-K electron energy-loss spectroscopy (EELS) spectra acquired from a bright column and a dark column boundary in Fig. 4E shows that the column boundaries are Ti-rich compared to the columns.

Cross-sectional EELS analysis was carried out to explore the influence of implementing identical |1.0|-V spikes on device electronic structures. Figure 4 (G to I) compares the Ti-L_2,3_, O-K, and N-K EELS spectra of pristine and programmed devices, acquired from regions indicated in Fig. 4F. For all three conditions, Ti valence states change from pure Ti^3+^ (featured by an ∼5.2-eV spin-orbit coupling of Ti 2p electrons) in unoxidized TiN to a mixture of Ti^3+^ and Ti^4+^ (doublets indicating the octahedral t_2g_-e_g_ splitting) in TiO_x_N_y_, to pure Ti^4+^ near the TiO_x_N_y_/Hf(Sr,Ti)O_2_ interface, to dominant Ti^3+^ inside Hf(Sr,Ti)O_2_. The O-K doublets shift by ∼2.4 eV toward higher energy-loss values from TiO_x_N_y_ to Hf(Sr,Ti)O_2_ due to substantial changes in the electronic environment and core exciton–binding energies. The N-K spectra acquired from TiN have two sharp overlapping peaks and a broad shoulder, while N signals were not detected inside Hf(Sr,Ti)O_2_.

While programming the device does not notably influence on the shape of the EELS signals from bulk TiN and Hf(Sr,Ti)O_2_, the spectra acquired from the interfaces, regions 2 and 3 in Fig. 4F, exhibit differences. Figure 4 (G to I) and fig. S7 reveal that the e_g_/*t*_2g_ and O″/O′ ratios relatively increase, regardless of spike polarity. Yet, the N-K signals are dependent on the polarity of spikes. The N″/N′ ratios and N° peak intensities are considerably increased by implementing positive spikes, whereas they almost disappear upon negative-spike applications. These changes can be caused by electronic and ionic charge motion across TiO_x_N_y_, distinctly manifested in the N-K signals from the TiN/TiO_x_N_y_ interfaces. Upon positive-spike applications, negatively charged oxygen ions drift from TiN into TiO_x_N_y_, reducing TiN and increasing the relative intensities of N″ and N° peaks. However, oxidizing TiN through reversing the spike polarity decreases these peaks’ intensities (*61*, *62*).

The ionic migration is further revealed by determining absolute EELS volumetric-density depth profiles (Fig. 4, J to L). At the pristine state, Fig. 4J shows gradients of O and N inside TiO_x_N_y_, agreeing with the RBS data. While training the device with positive spikes results in ionic drift toward Hf(Sr,Ti)O_2_ (particularly for O anions), applying negative spikes reverses the ionic motion direction. Moreover, O and N signals are not detected in Mo layers after device programming.

**Fig. 5.**
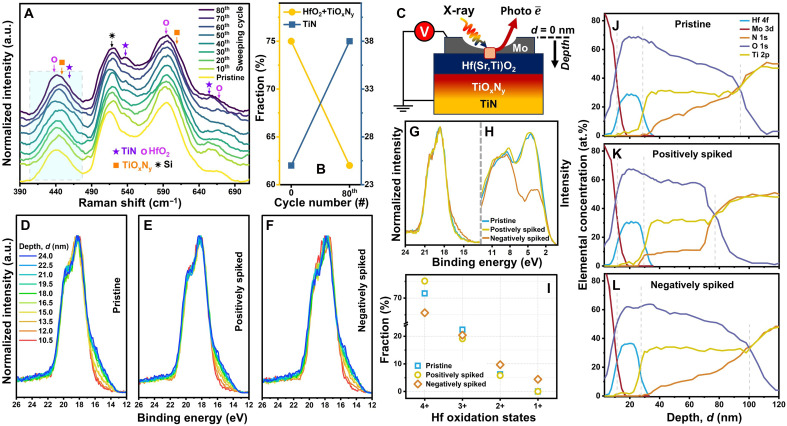
Materials characterization using Raman and XPS. (**A**) Normalized Raman spectra determined from in situ measurements during applying 80 voltage-sweeping cycles up to +1.0 V. (**B**) Area fractions under TiN and (TiO_x_N_y_ + HfO_2_) components of broad Raman peaks appearing between 400 and 480 cm^−1^ for pristine and 80 time-cycled states. (**C**) Schematic illustration of XPS depth-profile measurements. Approximately 16-nm-thick circular-shape Mo contacts were sputter-deposited on the Hf(Sr,Ti)O_2_/TiN surface to electrically pulse the devices, preserve the interfaces from air exposure, and ensure acquiring intact core levels from some certain depths into Hf(Sr,Ti)O_2_. Mo surfaces are assigned to depth *d* = 0 nm. Intensity-normalized Hf 4f core-level spectra plotted without any *y*-axis offset as a function of depth *d* acquired from (**D**) pristine, (**E**) positively, and (**F**) negatively spiked (|1.0| V) devices. (**G**) Intensity-normalized, peak shift–corrected Hf 4f core-level and (**H**) as-measured valence-band spectra acquired from pristine and spiked devices at depths *d* = ~13.5 nm. (**I**) Relative fractions of Hf oxidation states obtained from deconvoluting the Hf 4f core-level spectra shown in (G). Relative elemental-concentration depth profiles of (**J**) pristine, (**K**) positively, and (**L**) negatively spiked devices as a function of depth *d*. Overestimated XPS-obtained O concentrations compared to RBS data are due to O redeposition during Ar-ion sputter-etching process and XPS systematic uncertainties.

Figure 5B compares normalized Raman spectra obtained from in situ measurements during applying 80 sweeps from 0 to +1.0 V. Upon positive-voltage applications, there are evolutions in the intensities and shapes of TiN components. The intensities of convoluted TiN peaks gradually increase. Moreover, the area under the broad peak appearing between 400 and 480 cm^−1^ increases by 16% (fig. S8). Deconvoluting the peaks obtained at pristine and 80 time-cycled states reveals that the area under the TiN component increases by 13% (Fig. 5B). These indicate a general upward oxygen diffusion that depletes the TiO_x_N_y_ layer of oxygen and relatively increases the thickness of the N-rich region of TiO_x_N_y_. One may infer that negative electric-field application would yield an opposite effect; thus, the influences of both spike polarities are explored comprehensively below using x-ray photoelectron spectroscopy (XPS).

Depth-resolved XPS measurements were conducted to investigate the oxidation states and elemental reconfigurations through the thickness of the devices (see Fig. 5C), at pristine, positively and negatively spiked (|1.0| V) conditions. Figure 5 (D to F) and fig. S9 show the depth-resolved Hf 4f core-level spectra acquired at depths *d* = ~10.5 to ~24.0 nm. For each condition, all peaks appear at the same position, proving that there is no detectable Hf 4f chemical shift in Hf(Sr,Ti)O_2_ as a function of depth. While the Hf 4f_7/2_ signals from the pristine and positively spiked devices appear at ~18.2 eV, the signals from the negatively spiked device arise at ~17.7 eV. This ~0.5-eV chemical shift can be attributed to oxygen migration from Hf(Sr,Ti)O_2_ into TiO_x_N_y_ upon the negative-spike application that increases the number of broken Hf-O bonds; indirectly indicating oxygen vacancy (*V*_O_) formation in Hf(Sr,Ti)O_2_. There are also shoulders appearing at the lower binding energies, implying the existence of Hf with lower oxidation states and possible core-level damage contributions (discussed in fig. S9).

Figure 5 (G and H) compares the Hf 4f core-level and valence-band spectra acquired at *d* = ~13.5 nm. These regions are still in the Mo electrode but as close to Hf(Sr,Ti)O_2_ as possible so that signals from the intact Hf 4f core levels were acquired without being affected by Ar ion sputter etching. The Hf 4f peaks for all three states have the same widths and shapes, except for the low–binding energy shoulder that is larger and wider for the negatively spiked device. We deconvoluted these peaks to obtain Hf oxidation states (see fig. S10). While the Hf 4f spectra of the pristine and positively spiked devices comprise Hf^4+^, Hf^3+^, and Hf^2+^ doublets, Hf(Sr,Ti)O_2_ in the negatively spiked device has all four Hf^n+^ oxidation states. Figure 5I shows that Hf^4+^ doublets are the dominant components for all three conditions. Applying positive spikes results in an increase of ~4% in the Hf^4+^ fraction compared to the pristine device, whereas negative spikes decrease this fraction by ~6%. The Hf 4f spectrum of negatively spiked device has more Hf^2+^ than the other devices. Similar changes were not observed in O 1s core-level spectra (further discussed in fig. S11). Overall, while applying positive spikes slightly further oxidizes Hf(Sr,Ti)O_2_, negative spikes reduce Hf(Sr,Ti)O_2_ that along with the observed Hf 4f chemical shifts supports the *V*_O_ formation upon negative-spike applications.

Normalized Mo 3d core-level spectra in fig. S12 do not show any notable peak broadening and shift, indicating that there are no detectable chemical changes at the Mo/Hf(Sr,Ti)O_2_ interface upon applying training spikes, consistent with our EELS data. Compared to the Hf 4f core-level changes, implementing negative spikes has a more substantial effect on the valence band of Hf(Sr,Ti)O_2_. The as-measured valence-band spectra for all three conditions (Fig. 5H) have two wide, semiconvoluted signals appearing with no chemical shift. While ~7.4-eV components have similar intensities, the intensity of ~2.6-eV component for the negatively spiked device is almost half of those for pristine and positively spiked devices, which is another indirect fingerprint for *V*_O_ formation.

Figure 5 (J to L) compares the relative elemental-concentration depth profiles of the pristine and spiked devices. While Hf(Sr,Ti)O_2_ contains ~70 at % O in the pristine and positively spiked devices, applying negative spikes leads to a decrease of ~10 at % in O, supporting the Hf(Sr,Ti)O_2_ reduction. Agreeing with the RBS and EELS data, the XPS depth profile of the pristine device demonstrates the O and N gradients in TiO_x_N_y_. Implementing positive spikes results in a substantial diffusion of O and N from TiO_x_N_y_/TiN toward the Hf(Sr,Ti)O_2_/TiO_x_N_y_ interface, while changing the spike polarity leads to downward O and N diffusion toward TiN. However, in all three cases, Ti, without any notable change in its concentration, is uniformly distributed along the TiO_x_N_y_ layers, indicating that it does not move during device programming, in agreement with EELS depth profiles in Fig. 4 (J to L).

## DISCUSSION

### p-type electronic conductivity in Hf(Sr,Ti)O_2_

Opposite to most HfO_2_-based memristors that show dominant filamentary switching (*63*, *64*), these devices exhibit bipolar diode–like characteristics with pure interfacial conductance changes. To determine the key modulating interface, we first evaluated the TiO_x_N_y_/TiN stack by removing Hf(Sr,Ti)O_2_ and studying Au/Cr/TiO_x_N_y_/TiN devices. The corresponding *I*-*V* curves in fig. S13 do not exhibit any hysteretic loops. Replacing Mo electrodes with Ti and Au also does not change the shapes of the corresponding *I*-*V* curves; see figs. S14 and S15. The XPS measurements around the Mo/Hf(Sr,Ti)O_2_ interfaces do not show any detectable Mo oxidation upon applying training spikes (fig. S12). These results confirm that the TiO_x_N_y_/TiN and Mo/Hf(Sr,Ti)O_2_ interfaces do not notably influence the conductance changes.

Therefore, we postulate that the observed ultralow conductance modulation is primarily attributed to the formation of a p-n–like heterointerface between p-type Hf(Sr,Ti)O_2_ and n-type, O-rich TiO_x_N_y_. The asymmetric *I*-*V* curves of the Mo/Hf(Sr,Ti)O_2_/TiN devices consist of a current rectification at positive voltages that follows a substantial increase in resistance during the reverse voltage sweeps. We compared the *I*-*V* curves of these devices with those of Mo/Hf(Sr,Ti)O_2_/La_0.7_Sr_0.3_MnO_3_/SrTiO_3_ that form a Schottky-dominated bottom interface (fig. S16). The rectification ratio is ~155 for Mo/Hf(Sr,Ti)O_2_/TiN and ~2.6 for Mo/Hf(Sr,Ti)O_2_/La_0.7_Sr_0.3_MnO_3_. The considerable increase in resistance during reverse voltage sweeps together with the pronounced rectification ratio observed in the Mo/Hf(Sr,Ti)O_2_/TiN devices are typical electrical characteristics of p-n diodes. In addition, to further evaluate the polarity dependence, we initially applied a negative sweep (0 V → −3.5 V → 0 V). No measurable SET transition or memory window was observed. After switching to a positive sweep (0 V → +3.5 V → 0 V), the device exhibited clear hysteresis and reproducible bipolar behavior, as shown in Fig. 1A. This confirms that switching initiates under forward bias, consistent with the built-in p-n heterointerface asymmetry.

**Fig. 6.**
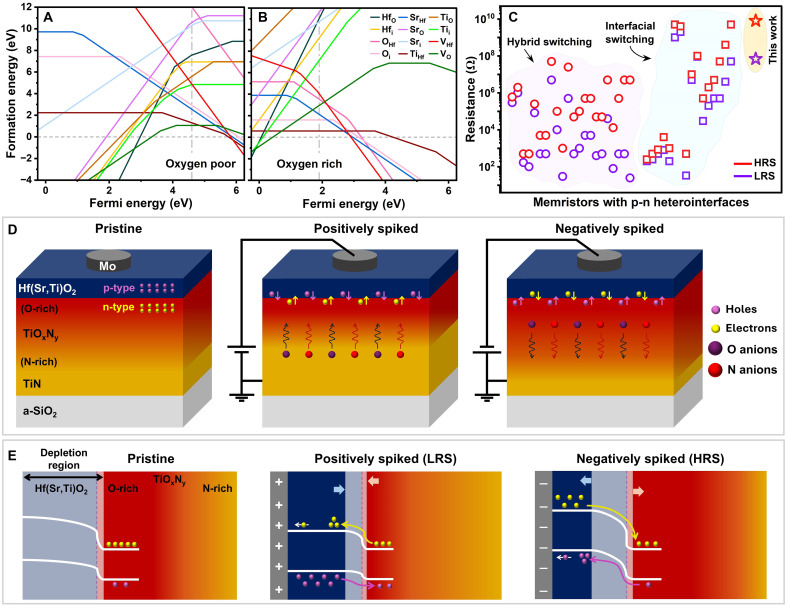
Principal characteristics and conductance modulation mechanism model. Defect and dopant formation energies calculated for (**A**) understochiometric (O-deficient) and (**B**) stochiometric (O-rich) Hf(Sr,Ti)O_2_ at equilibrium. (**C**) LRS and HRS values of this-work and ~40 reported memristors with p-n heterointerfaces that are made of different materials, including n-type TiO_2_, and show interfacial or hybrid filamentary-interfacial resistive switching. (**D**) Schematic illustration summarizing the conductance modulation model proposed for the Mo/Hf(Sr,Ti)O_2_/TiN/a-SiO_2_/Si devices and (**E**) corresponding energy band diagrams changing upon spike applications.

The n-type semiconductor nature of TiO_2_ is already well known (*65*). However, p-type electronic conductivity in Hf(Sr,Ti)O_2_ requires substantiation although it is expected on the basis of our work’s hypothesis, creation of p-type donors from lower-valent ion doping on Hf^4+^ sites (*66*, *67*). Thus, we conducted a series of ab initio calculations and Hall effect measurements to ascertain p-type electronic conductivity in Hf(Sr,Ti)O_2_. The equilibrium energy–level diagram of Hf(Sr,Ti)O_2_ in Fig. 6A indicates that inducing p-type conductivity under O-deficient conditions is unlikely because of charge compensation effects (*68*). However, in O-rich environments, although Ti does not have any p-type doping influences as its defect levels are situated well above the valence band maximum (VBM), we found that Sr dopants can effectively lower the Fermi level below the equilibrium state (Fig. 6B). Our carrier concentration calculations also indicate that holes are the majority charge carriers. These findings uncover that Sr acts as a p-type dopant in stoichiometric Hf(Sr,Ti)O_2_. Consistent with these results, the positive Hall coefficient in fig. S17 further corroborates the presence of p-type conduction in Hf(Sr,Ti)O_2_. We believe that the key reasons for the formation of p-type Hf(Sr,Ti)O_2_ stem from the synergistic effects of (i) engineering its bandgap and (ii) substituting Hf^n+^ in the Hf cation sublattice with Sr^2+^ acceptor dopants. For stoichiometric layers, ultraviolet-visible (UV-vis) measurements and density-of-state calculations reveal a decrease in the bandgap from ~6.0 eV for HfO_2_ to ~4.5 eV for Hf(Sr,Ti)O_2_, which is mainly attributed to adding Ti dopants (see figs. S18 and S19). This substantial bandgap reduction largely overcomes doping limitations associated with wide-bandgap HfO_2_ (*68*, *69*), enabling Sr in an O-rich environment to increase the concentration of holes and thus, effectively shift the Fermi level closer to VBM.

Figure 6C compares the LRS and HRS values of our devices with ~40 different memristors of wide-ranging systems reported to form p-n heterointerfaces. These memristors show interfacial or hybrid filamentary-interfacial resistive switching (detailed in table S2 and fig. S20). Our devices are distinguished by their highest resistance states and largest interfacial-memristive memory window.

### Conductance modulation mechanism model

We propose that the analog conductance modulation in our devices occurs via two main mechanisms that operate concurrently: (i) transfer of electronic charge carriers across the Hf(Sr,Ti)O_2_/TiO_x_N_y_ interface and (ii) electromigration of negative ionic carriers inside the bulk TiO_x_N_y_ and Hf(Sr,Ti)O_2_ layers, as schematically illustrated in Fig. 6D. Figure S21A shows the isolated energy band diagrams of TiN, O-rich TiO_x_N_y_, Hf(Sr,Ti)O_2_, and Mo, where the Fermi energy level lies close to VBM inside p-type Hf(Sr,Ti)O_2_, while it is near the conduction band minimum in n-type O-rich TiO_x_N_y_. At the pristine state, the devices are initially in their HRS due to a large built-in potential generated at the p-Hf(Sr,Ti)O_2_/*n*-TiO_x_N_y_ heterointerface (fig. S21B). Defective TiO_x_N_y_ has a substantially higher carrier concentration than stochiometric Hf(Sr,Ti)O_2_. Thus, the built-in potential mainly forms inside Hf(Sr,Ti)O_2_, creating a depletion region asymmetrically extended in this layer (Fig. 6E). The thickness of this region can exceed that of Hf(Sr,Ti)O_2_, as further discussed in fig. S21.

By applying positive spikes, holes and electrons are gradually injected into the depletion region and n-type O-rich TiO_x_N_y_, respectively (see Fig. 6E). The charge carriers are increasingly captured into trapping sites such as point defects, mainly vacancies that typically have high concentrations in the lattice of sputter-deposited layers and cations in the Hf(Sr,Ti)O_2_ sublattice. This agrees with our *I*-*V* curve analyses indicating that the conduction is governed by the space charge–limited current model (further details in fig. S22). The carrier transfer during device programming is also indirectly demonstrated by our XPS and EELS data. Thus, the controlled carrier transport steadily decreases the thickness of the depletion region, lowers the barrier height at the p-n heterointerface, and hence, increases the device conductance in an analog manner. The remarkably low conductance values of our devices can be mainly attributed to two key factors: (i) wide depletion region and (ii) limited defect concentration (mainly *V*_O_) and hole mobility inside stoichiometric, p-type Hf(Sr,Ti)O_2_, which the latter is substantially lower than in typical p-type semiconductors (*70*).

In addition to the interfacial electronic carrier transfer, our measurements also revealed bulk ionic drifts upon the positive-spike applications. The results indicate that the negatively charged N and O ions undergo a long-range diffusion inside TiO_x_N_y_ toward Hf(Sr,Ti)O_2_. This gradually reduces TiO_x_N_y_ and increases the thickness of the N-rich region and hence, the TiO_x_N_y_ conductivity. This ionic-drift process can also decrease the width of depletion region at the p-n heterointerface (*71*), contributing to increasing the device conductance upon positive spiking.

Conversely, inverting the spikes’ polarity generates a reverse current, the depletion region becomes wider, trapped carriers are released from the trapping sites, and negatively charged ions electromigrate from Hf(Sr,Ti)O_2_ toward the TiO_x_N_y_/TiN interface, which further oxidizes TiN and extends the TiO_x_N_y_ thickness. The asymmetric conductance changes obtained upon applying identical negative spikes can be mainly related to the asymmetric nature of the space-charge region that is much wider inside Hf(Sr,Ti)O_2_ than TiO_x_N_y_, originating from the unequal defect and carrier concentrations in Hf(Sr,Ti)O_2_ and TiO_x_N_y_. We also attribute the excellent device-to-device uniformity observed in our devices’ resistive switching and neuromorphic performance to the laterally uniform distribution of point defects in the oxide layers and the p-n heterointerface.

In summary, we introduce ultralow current (≤10^−8^ A) analog memristive devices fabricated from fully complementary metal-oxide semiconductor compatible materials of p-type Hf(Sr,Ti)O_2_ on *n*-type TiO_x_N_y_ (on TiN/SiO_2_/Si). The devices show interfacial nonvolatile resistive switching with outstanding cycle-to-cycle and device-to-device uniformities and high retention (>10^5^ s). They also demonstrate several hundreds of stable and replicable conductance levels over neuromorphic timescales ranging from ~2.5 × 10^−9^ to ~1.4 × 10^−7^ S, with a conductance-modulation range of >50 (without reaching any saturation), obtained by applying identical 1.0-V spikes. Outstanding operational synaptic stability of these Hf(Sr,Ti)O_2_-based devices, essential for spiking neural networks and AI hardware, is confirmed by applying ~40,000 electronic spikes. Essential for the bioinspired synaptic electronic systems, these devices also demonstrate a reproducible emulation of key neural learning rules such as short-term synaptic plasticity and STDP. The unique performance originates from the formation of a self-assembled p-n heterointerface between p-type Hf(Sr,Ti)O_2_ and n-type TiO_x_N_y_, leading to a completely depleted space-charge region that is asymmetrically extended into Hf(Sr,Ti)O_2_, a large built-in potential, and a substantially low saturation current density under reverse bias. The achieved ultralow conductance modulation is controlled by gradually changing the energy-barrier height of the p-n heterointerface through electro-ionic charge migration. Our unique synaptic electronic devices overcome the energy-consumption and variability challenges of current memristors and pave the way toward energy-efficient neuromorphic technologies.

## MATERIALS AND METHODS

### Thin-film growth

TiN and Hf(Sr,Ti)O_2_ thin films were sputter-deposited in a chamber equipped with 5.08-cm (2-inch) Ti, HfO_2_, and SrTiO_3_ targets on commercial Si(001) with a 200-μm-thick amorphous SiO_2_ top layer as substrates. The a-SiO_2_/Si substrates were mounted on a substrate holder at the top of the growth chamber that was rotated at a speed of 20 rpm during the thin-film growth to provide uniform compositions and thicknesses. The system base pressure was <5.0 × 10^−8^ torr (0.67 × 10^−5^ Pa). Before each deposition, targets were presputtered in a pure Ar atmosphere under closed shutters for 1 hour. Thereafter, the growth chamber was baked for 12 hours and then coated with pure Ti for 1 hour.

In the beginning, TiN was deposited on the a-SiO_2_/Si substrates using the Ti target by ion-assisted dc magnetron sputtering in a reactive nitride atmosphere. TiN layers have an electrical resistivity of ~0.3 × 10^−7^ Ω·m, N/Ti ratio of ~0.96, and density and surface roughness of ~5.6 g/cm^3^ and ~0.5 nm, respectively (*72*). Thereafter, the multicomponent oxide thin films were grown on TiN/a-SiO_2_/Si in the same chamber at 700°C by radio frequency magnetron cosputtering from HfO_2_ and SrTiO_3_ targets, following two steps: First, they were sputter-deposited in a nonreactive atmosphere ($P_{O_{2}}$ = 0 SCCM) for 600 s and then oxygen with $P_{O_{2}}$ = 20 SCCM was immediately added to the growth chamber for 200 s, without changing other deposition parameters. Excluding the oxygen diffusion, this growth design could theoretically result in a double-layer formation: (i) an ~15-nm-thick under-stoichiometric Hf(Sr,Ti)O_1.8_ bottom layer and (ii) an ~1-nm-thick stoichiometric Hf(Sr,Ti)O_2_ top layer. Further details about the Hf(Sr,Ti)O_2_ thickness optimization and its relation to device performance are provided in the Supplementary Materials.

### Device fabrication

The device fabrication was carried out firstly by spin-coating a layer of positive UV photoresist (AZ 4533) on the surface of Hf(Sr,Ti)O_2_/TiN/a-SiO_2_/Si. The samples were then baked at 100°C for 2 min and exposed to UV light for 10 s through a photolithography mask. The resist was thereafter developed in an AZ351B developer. Then, the samples were coated with Mo by dc magnetron sputtering. Afterward, the samples were immersed in ethanol without any sonication to remove the unexposed UV resist and lift off the metal on its top. Last, they were baked for 10 min at 60°C to evaporate ethanol’s residuals. This process resulted in fabricating circular-shaped Mo top electrodes with different diameters on Hf(Sr,Ti)O_2_/TiN/a-SiO_2_/Si. A top-view image of a typical device is provided in fig. S23.

### Electrical and neuromorphic measurements

The Mo/Hf(Sr,Ti)O_2_/TiN/a-SiO_2_/Si devices were used for memory and neuromorphic characterizations. Throughout the project, we followed a standard protocol of storing all samples in a dry, controlled-atmosphere cabinet and performing all electrical measurements under similar controlled laboratory ambient conditions. This minimizes potential variability from adsorbed moisture or oxygen (*73*). In all measurements, which were carried out at room temperature, the voltage was applied to the Mo top electrodes, while the TiN bottom electrode was grounded. The conductivity of the grounded area was a few ohms. The nonvolatile memory and neuromorphic performance of these devices were obtained using a computer-controlled Keysight B2912A connected to a probe station.

STDP data were obtained by measuring weight changes via applying the superposition of voltage waveforms from presynaptic and postsynaptic spikes. Each synaptic profile consisted of two triangular parts with |1.0|-V amplitude and 10-ms width, using linear rise and fall times of 5 to 15 ms. The voltage waveforms were systematically moved with respect to each other as a function of the time difference between presynaptic and postsynaptic spikes Δ*t* and then subtracted from each other. For each Δ*t* value, the synaptic weight change Δ*W* was extracted from the average of 50 recorded data points. STDP behavior was verified across at least five different devices via two conventional programming schemes: paired spikes with similar and opposite polarities.

### Hall measurements

Hall measurements were undertaken on Hf(Sr,Ti)O_2_ grown on quartz substrates in a DynoCool physical property measurement system, from Quantum Design.

### Materials characterization

#### Rutherford backscattering spectrometry

The elemental compositions of the Hf(Sr,Ti)O_2_ thin films grown on the TiN/a-SiO_2_/Si substrates were obtained by RBS in a 5-MV 15SDH-2 tandem accelerator. 2-MeV ^4^He^+^ ions were used for RBS measurements, and backscattered ions were detected at a scattering angle of 170°. Possible ion-channeling effects were minimized by adjusting the equilibrium incidence angle to 5° with respect to the surface normal and performing multiple small random-angular movements within a range of 2° during data acquisition (*74*).

#### XRD and XRR

XRD and x-ray reflectivity (XRR) scans were carried out in a PANalytical Empyrean high-resolution x-ray diffractometer operated at 45 kV and 40 mA with a Cu K_α_ source (λ = 0.15406 nm) to determine the crystal structure, thickness, roughness, and density of the thin films.

#### Scanning transmission electron microscopy

Cross-sectional STEM analyses were carried out in a monochromated probe C_s_ aberration-corrected Thermo Fisher Scientific Spectra 300 electron microscope operated at 300 kV monochromated <100 meV at 100 pA. Images were acquired in both ABF and HAADF modes. EELS spectra were acquired using a Gatan Continuum 1066 EELS spectrometer energy resolution of 150 meV per channel integrated over 1 s. Absolute volumetric density spectra for N-K, Ti-L_2,3_, and O-K edges were calculated using Gatan Microscopy Suite 3.52 with implemented routines for multiple linear least squares fitting models with Hartree-Slater scattering cross sections and including zero-loss centring, power law background subtraction, plural scattering removal, and excluding energy-loss near edge structure regions of 20 eV. TEM specimens were prepared by focused ion beam technique using a FEI Helios Nanolab DualBeam instrument.

#### Raman spectroscopy

In situ Raman spectroscopy was performed using Integrated Optics’ continuous wave laser at a single longitudinal mode, wavelength of 633 nm, and power of 1.5 mW. Raman signals were transmitted to an Andor Kymera 328i spectrometer connected to an Oxford Instruments’ Newton EMCCD camera. The sample was electrically connected to a 7-nm-thick conductive top electrode (Au/Cr) to enable in situ Raman measurements. Instead of a probe tip, the potential was applied to the sample with a conducting cantilever to avoid puncturing the top electrode into the sample. Apex Probes cantilever tips were coated with a 3-nm-thick Cr/6-nm-thick Au layer to apply potential. The Keithley 2634B source meter was used for in situ electrical measurements.

#### X-ray photoelectron spectroscopy

Depth-resolved XPS core levels were obtained in a Thermo Fisher Scientific Escalab 250Xi instrument with monochromatic Al K_α_ radiation (ℎ𝜈 = 1486.6 eV) at ~1 × 10^−9^ Torr using 400-eV Ar-ion surface sputter-etching. To prevent the destructive influences of sputter-etching on XPS core levels and fabricate top electrodes, after the Hf(Sr,Ti)O_2_ growth, each sample was immediately transferred to an inert-atmosphere glove box, then a shadow mask was fixed on top of that and immediately afterward was loaded into another sputtering chamber, dedicated to metal deposition. After sputter-depositing circular-shape Mo top electrodes (with thicknesses and diameters of ~16.0 and ~1.0 mm, respectively) on the Hf(Sr,Ti)O_2_/TiN/a-SiO_2_/Si surface, two devices were positively and negatively spiked (|1.0| V), and last, the sample was immediately transferred to the XPS instrument. During all transport steps, the sample was kept in a vacuum vessel, which could be connected to both glove box and XPS instrument. This preparation protocol minimized the sample air exposure. The Mo thin films were kept in electrical contact with the XPS sample holder to prevent possible peak shifts resulting from sample charging. Depth-resolved, high–energy resolution XPS core levels were acquired from 400 μm–by–400 μm regions located in the center of 1 mm–by–1 mm sputter-etched areas. Elemental quantification and Hf 4f XPS peak deconvolution were carried out using CasaXPS software. XPS depth scales (time) were converted to depth (nanometer) by using the average thicknesses obtained from STEM images.

#### UV photoelectron spectroscopy

Work functions were determined by UV photoelectron spectroscopy measurements conducted in Thermo Fisher Scientific Escalab 250Xi instrument using He I radiation (ℏω = 21.22 eV). To remove native surface oxides, sample surfaces were gently sputter-etched using 200-eV Ar ions.

#### UV-vis spectroscopy

The optical energy bandgaps (*E*_g_) of stoichiometric HfO_2_ and Hf(Sr,Ti)O_2_ layers sputter-deposited on quartz substrates were determined from their absorption spectra acquired using a Shimadzu UV-3600i plus spectrophotometer, following the Tauc method (*75*): (α·ℎ𝜈)^2^ = *A*(ℎ𝜈 − E_g_), where α, ℎ, and 𝜈 are the absorption coefficient, Planck’s constant, and photon’s frequency, and *A* is a constant. The *E*_g_ values were obtained by extrapolating the linear parts of the Tauc plots, (α·ℎ𝜈)^2^ versus ℎ𝜈, to *x* axis intersection points.

### Ab initio calculations

First-principles calculations were conducted using the Vienna ab initio Simulation Package with spin polarization (*76*). A 550-eV cutoff energy was used for the plane wave basis set. The PAW pseudopotential (dataset version PBE_54) of Sr_sv, Ti_pv, Hf_pv, and O were used (*77*), with Heyd-Scuseria-Ernzerhof hybrid functional (HSE06) to obtain bandgaps closer to experimental values (*78*). AiiDA framework was used for data provenance and workflow automation with the aiida-vasp plugin (*79*). The initial orthorhombic phase HfO_2_ (space group of Pca2_1_) structure was obtained from the Materials Project (mp-685097). Full relaxation was performed using a force threshold of 0.03 eV/Å, with a Γ-centered *k*-point grid density of 0.05 × 2π Å^−1^. To calculate the formation energies of different defects, a 96-atom 2 × 2 × 2 supercell was constructed. The Brillouin zone of the supercell was sampled using a single Γ point to reduce computational costs. To ensure the reliability of this approach, we also calculated formation energies with a Γ-centered 2 × 2 × 2 *k*-point grid for several cases and found negligible differences. The formation energy of defects *D* in the charge state *q* can be expressed as

$$\Delta H_{D,q}\left(E_{F},\mu \right)=\left[E_{D,q}-E_{H}\right]+\sum_{i}n_{i}\mu_{i}+qE_{F}+E_{\text{corr}}$$

(*80*), where *E*_D,q_ and *E*_H_ are total the energies of defect and host supercell, respectively. μ_i_ is the chemical potential of type i atom, with n_i_ atoms added or removed to form the defect. *E*_F_ is the Fermi level, and *E*_corr_ is the adopted Kumagai-Oba finite-size charge correction (*81*). The preparation and postprocessing of each charged defect supercell were carried out using the doped package (*82*). Moreover, the defect concentration and self-consistent Fermi level were analyzed using py-sc-fermi (*83*).

## Supplementary Materials

### This PDF file includes:

Supplementary Text Figs. S1 to S23 Tables S1 and S2 References
