# Supplementary material for: HfO2-based memristive synapses with asymmetrically extended p-n heterointerfaces for highly energy-efficient neuromorphic hardware
Source: Sci Adv. 2026 Mar 20;12(12):eaec2324. doi: 10.1126/sciadv.aec2324 (PMC13004029; doi:10.1126/sciadv.aec2324)
Supplement: Supplementary file 1 — Supplementary Text Figs. S1 to S23 Tables S1 and S2 References [file sciadv.aec2324_sm.pdf]

Science Advances
AAAS

Supplementary Materials for

HfO_2-based memristive synapses with asymmetrically extended p-n heterointerfaces for highly energy-efficient neuromorphic hardware

Babak Bakhit *et al.*

Corresponding author: Babak Bakhit, bb643@cam.ac.uk

Sci. Adv. **12**, eaec2324 (2026)
DOI: 10.1126/sciadv.aec2324

This PDF file includes:

Supplementary Text
Figs. S1 to S23
Tables S1 and S2
References

Figure S1 shows the normalised weight (conductance) changes for the long-term potentiation and depression (LTP and LTD) of a typical Mo/Hf(Sr,Ti)O_2/TiN device obtained by implementing identical training schemes with 1.0-V, 2.0-V, and 3.0-V spikes. The nonlinear behaviour of the weight updates, referred to as the nonlinearity coefficient (φ), is determined by modelling the normalised potentiation and depression (W_{LTP} and W_{LTD}) data given in Fig. 2(A) in the main paper based on the following equations *(84)*:

$$W_{LTP} = B[1 - \exp(-F/A)] + W_{min}$$

$$W_{LTD} = -B[1 - \exp((F - F_{max})/A)] + W_{max}$$

$$B = (W_{max} - W_{min})/[1 - \exp(-F_{max}/A)]$$

$$\varphi = 1.726/(A + 0.162)$$

where W_{min} and W_{max} are the minimum and maximum weight values, respectively; F is the number of presynaptic spikes; F_{max} is the maximum number of implemented spikes; A is a constant that refers to the device nonlinearity characteristic; and B is a scaling parameter for normalising the curve to the conductance-modulation dynamic range (W_{max}/W_{min} ratio). The modelled curves yield the nonlinearity coefficients of φ = 0.11, 0.08, and 0.06 for LTP and φ = 0.40, 0.67, and 1.0 for LTD obtained from the schemes with 1.0-V, 2.0-V, and 3.0-V spikes, respectively. The φ values for LTP are close to the ideal value (φ = 0), while those of LTD are not. This asymmetric behaviour is mainly due to the device nature, further discussed in the conductance modulation mechanism model section in the main paper.

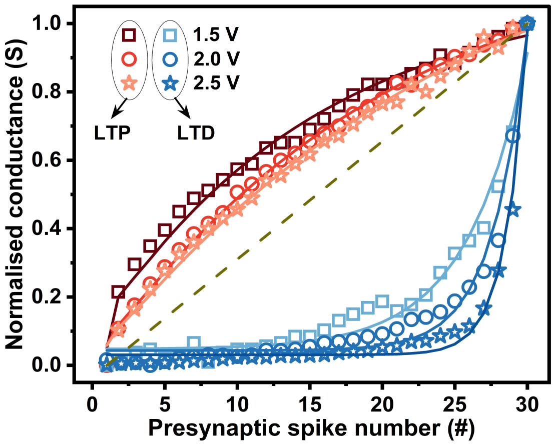

Fig. S1. Conductance updates. Normalised conductance updates together with their fits for the long-term potentiation and depression (LTP and LTD) of a typical Mo/Hf(Sr,Ti)O_2/TiN/a-SiO_2/Si device obtained by implementing identical training schemes with 1.0-V, 2.0-V, and 3.0-V spikes.

Figure S2 shows the artificial synaptic weights realised from an identical training scheme with 6000 positive and 6000 negative spikes (1.0-V spikes and dt = 1 ms) together with a representative subset of five intermediate conductance levels. After applying ~500 spikes, the conductance increase follows a highly linear behaviour.

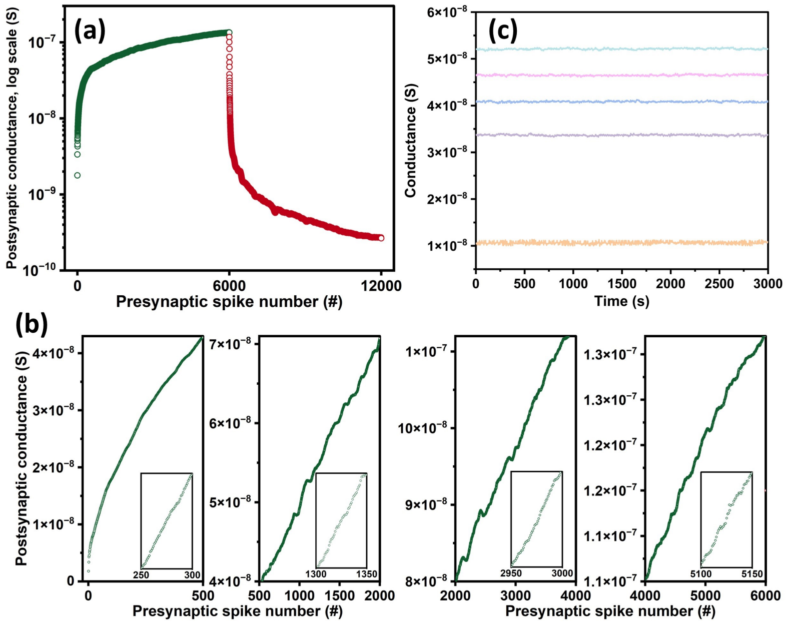

Fig. S2. Artificial synaptic weights. Synaptic weights realised from an identical training scheme with 6000 positive and 6000 negative spikes (1.0-V spikes and dt = 1 ms) together with a subset of five intermediate conductance levels.

Figure S3 shows the artificial synaptic weights obtained by implementing identical and non-identical training schemes for 39600 spikes (1320 consecutive cycles). For both cases, the potentiation and depression have highly stable weights and are appropriately symmetric. The non-identical scheme can be considered a harsh training programme that can more easily lead to device failure compared to the identical training scheme with 1.0-V spikes shown in Fig. S3(A). However, the data shown in Fig. S3(B) perfectly demonstrate highly stable conductance changes with an average W_{max}/W_{min} ratio of > 10, without any device failure.

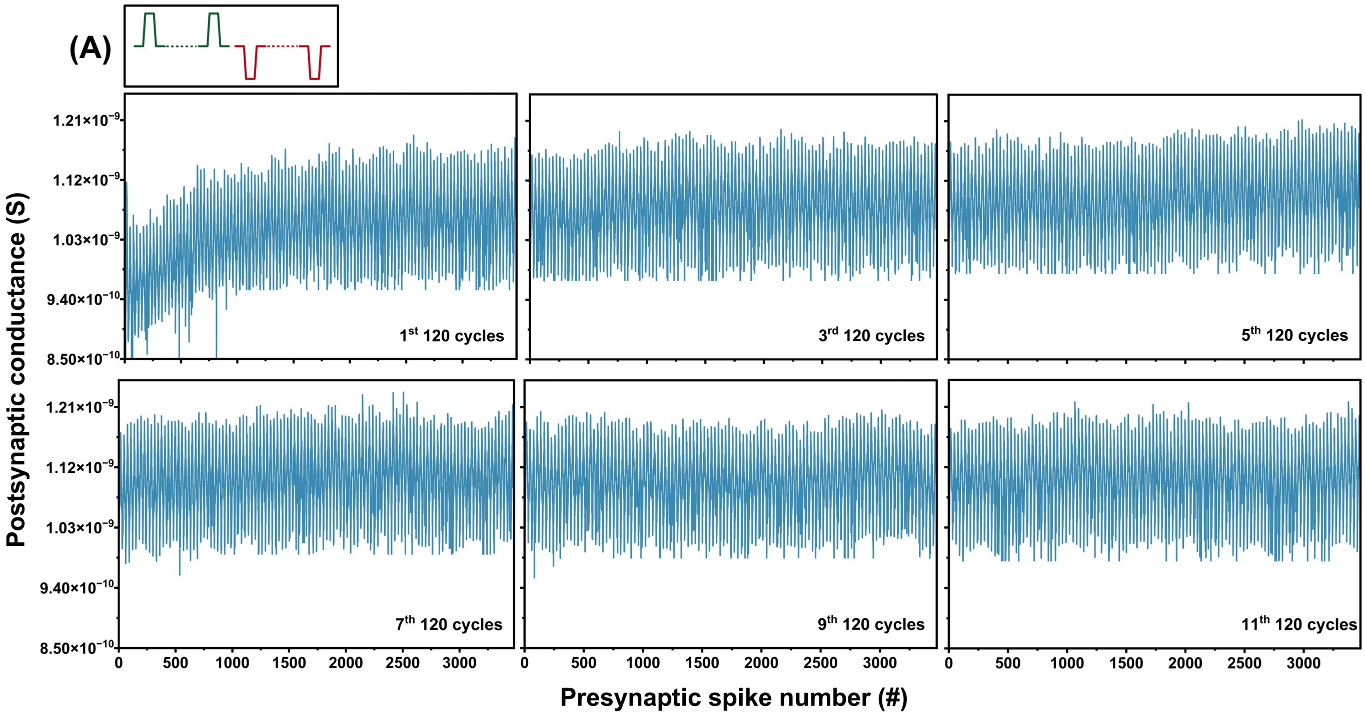

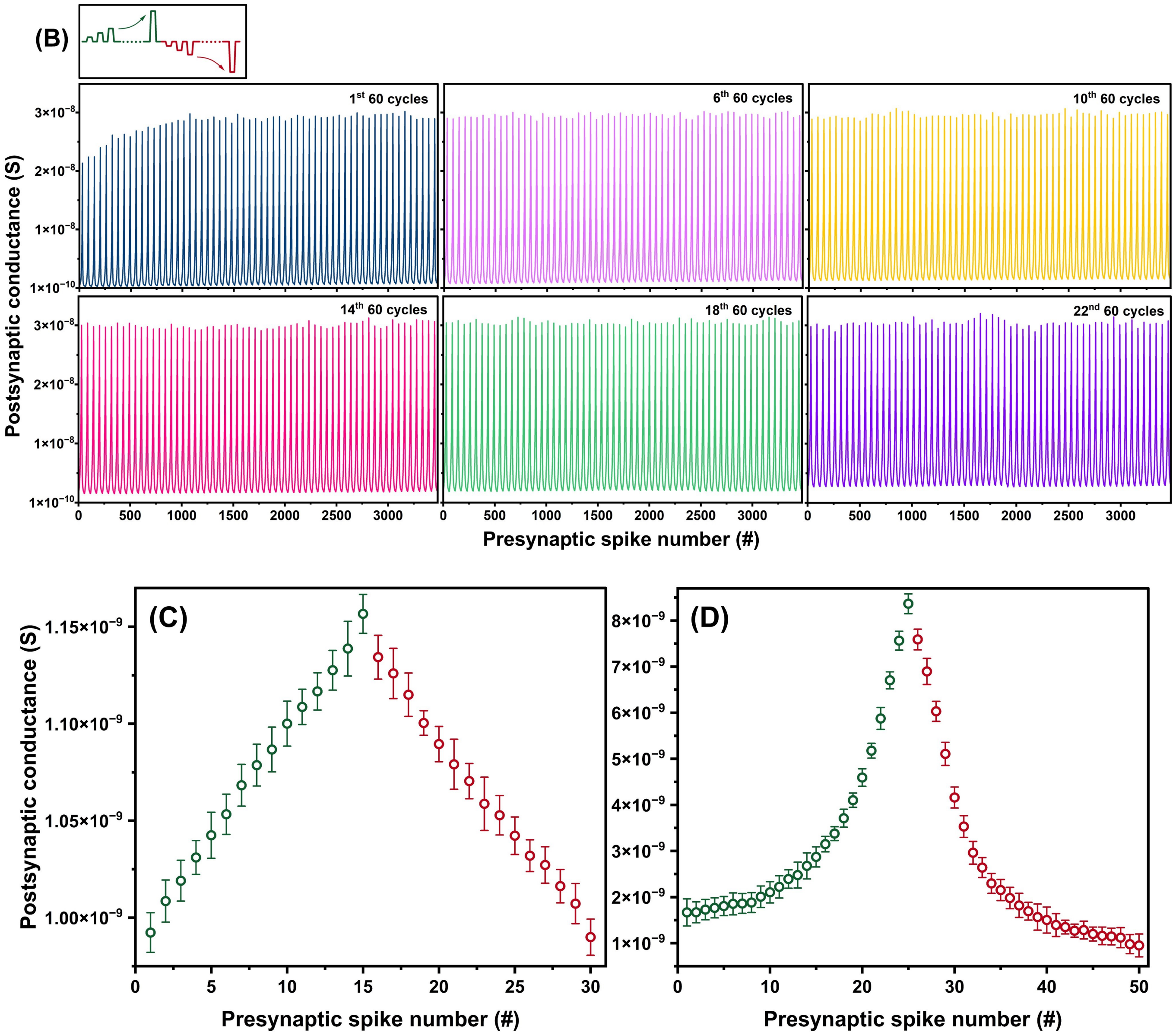

Fig. S3. Robust synaptic weights. Artificial synaptic weights realised by implementing (A) an identical training scheme with 1.0-V spikes and (B) a non-identical training scheme with amplitudes changing (increasing and decreasing) from ±0.6 to ±3.0 V in ±0.1-V increments (dt = 1 ms) for 39600 spikes (1320 consecutive cycles). Average values of the synaptic weights obtained by applying the (C) identical and (D) non-identical training scheme.

Figure S4 shows the relative asymmetric STDP weight changes ΔW for five different Hf(Sr,Ti)O_2-based devices, realised by implementing two different conventional programming schemes with triangle-shaped 1.0-V spikes: paired spikes with similar and opposite polarities.

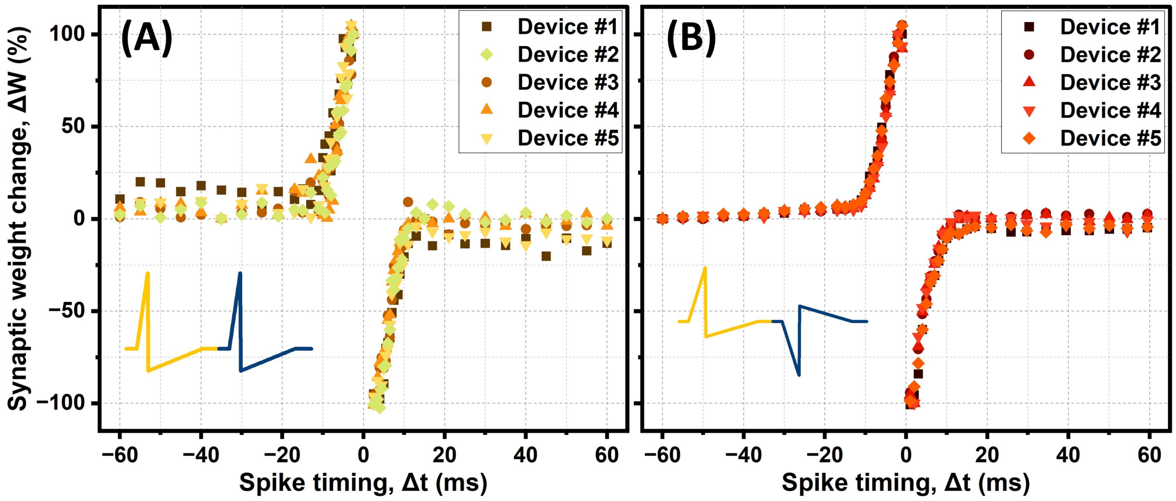

Fig. S4. STDP weight changes. Relative synaptic weight change ΔW as a function of time difference between presynaptic and postsynaptic spikes Δt with triangle-shaped 1.0-V spikes: paired spikes with (A) similar and (B) opposite polarities.

Figure S5 shows the RBS experimental spectra and SIMNRA simulations of the Hf(Sr,Ti)O_z thin films sputter-deposited on TiN/a-SiO_2/Si in the non-reactive atmosphere (P_{O_2} = 0 sccm) and following the two-step growth strategy. The O/metal ratio, z = O/(Hf + Ti + Sr), in the Hf(Sr,Ti)O_z thin film grown in the non-reactive atmosphere is ~1.80 (demonstrating an under-stochiometric oxide formation), while the z value for the multi-component oxide sputter-deposited following the two-step growth condition is ~2.0, proving the formation of an almost stochiometric oxide. In both cases, the multi-component oxides contain ~4.0 at.% Ti and ~3.6 at.% Sr. The slightly higher Ti concentration can be mainly due to their different sputtering yields.

Opposite to the RBS spectrum of the device with under-stoichiometric Hf(Sr,Ti)$O_{1.8}$ oxide that does not show TiN oxidation (Fig. S5(A)), the RBS spectrum in Fig. S5(B) indicates that the TiN bottom layer of the device with stoichiometric Hf(Sr,Ti)O_2 was oxidised. The corresponding depth profile, the inset in Fig. S5(B), reveals the formation of a thick TiO_xN_y layer at the Hf(Sr,Ti)O_2/TiN interface in which N gradually increases from ~2.0 at.% at regions close to the Hf(Sr,Ti)O_2/TiO_xN_y interface to ~46.5 at.% at the TiO_xN_y/TiN interface, while O decreases from ~65.0 to ~5.0 at.%. The Ti depth profile inside the TiN and TiO_xN_y layers remains almost constant at ~33.0 at.% and ~48.5 at.%, respectively.

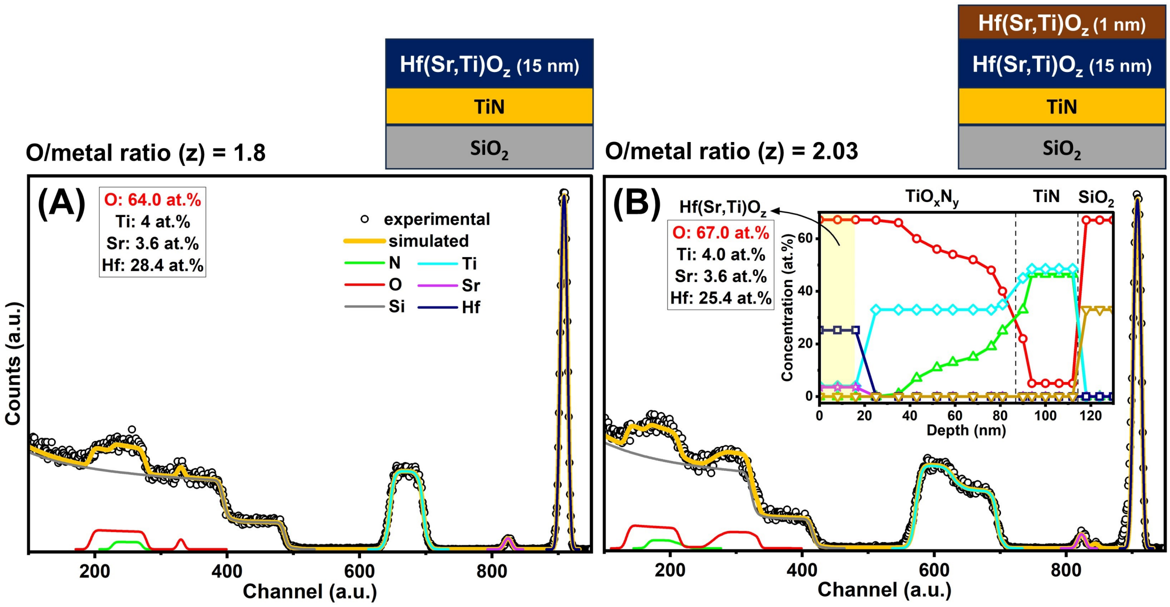

Fig. S5. RBS spectra. RBS experimental spectra and SIMNRA simulations of Hf(Sr,Ti)O_z thin films grown on TiN/a-SiO_2/Si (A) in the non-reactive atmosphere and (B) following the two-step growth strategy. The RBS depth scale (×10^{15} atoms/cm^2) was converted to depth (nm) by using the average thickness measured by STEM. The ideal thin film structure for each growth design is schematically illustrated on top of each panel. To minimise possible ion-channelling effects, the equilibrium incidence angle is adjusted to 5° with respect to the surface normal, and multiple-small-random-angular movements were carried out within a range of 2° during data acquisition.

Figure S6 compares the XRD θ-2θ scans of as-deposited TiN, Hf(Sr,Ti)$O_{1.8}$/TiN, and Hf(Sr,Ti)O_2/TiN. The peaks at 32.8° arise from the forbidden 002 Si(001) reflection that appears due to multiple scattering events. The highly dense and smooth TiN layers crystallise in a cubic crystal structure with a mixture of [111] and [200] orientations. The XRD pattern of Hf(Sr,Ti)$O_{1.8}$/TiN consists of the TiN peaks and an extra peak appearing at 2θ = ~30.3°, which can be assigned to the orthorhombic HfO_2-based phase with [111] orientation. This data indicates the formation of Hf(Sr,Ti)$O_{1.8}$ solid solution with no XRD-detectable phase separation. The θ-2θ scan of Hf(Sr,Ti)O_2/TiN preserves the TiN and HfO_2-based components but also has a set of extra peaks that can be assigned to the formation of tetragonal TiO_2(N) phase.

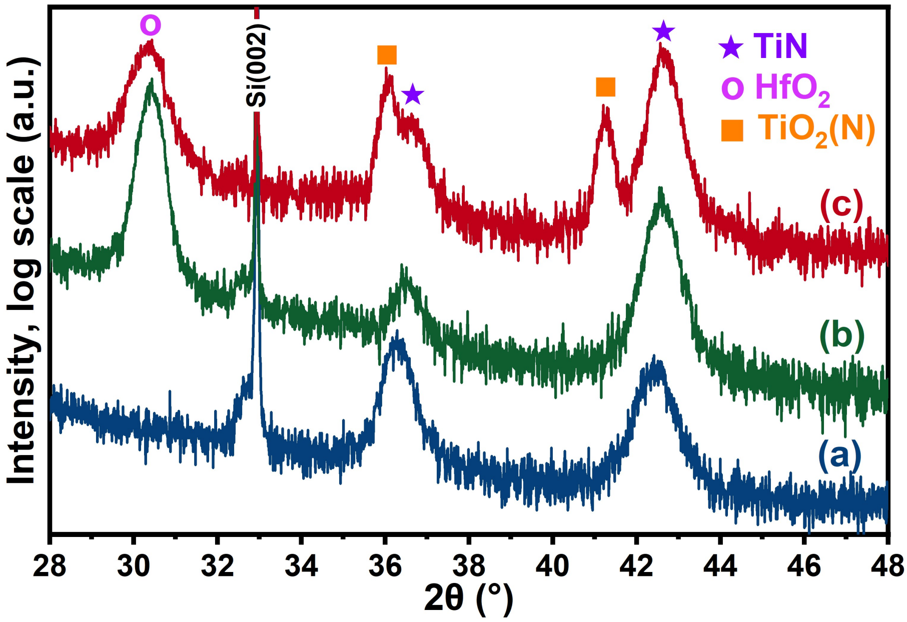

Fig. S6. XRD θ-2θ data. XRD scans of as-deposited (A) TiN, (B) Hf(Sr,Ti)$O_{1.8}$/TiN, and (C) Hf(Sr,Ti)O_2/TiN.

Figure S7 shows Ti-L, O-K, and N-K EELS spectra acquired from the TiO_xN_y/TiN interfaces in pristine state and after implementing positive and negative 1.0-V spikes. The signal intensities

were normalised, and the spectra were plotted with y-axis offset to reveal possible changes in peak positions and shapes.

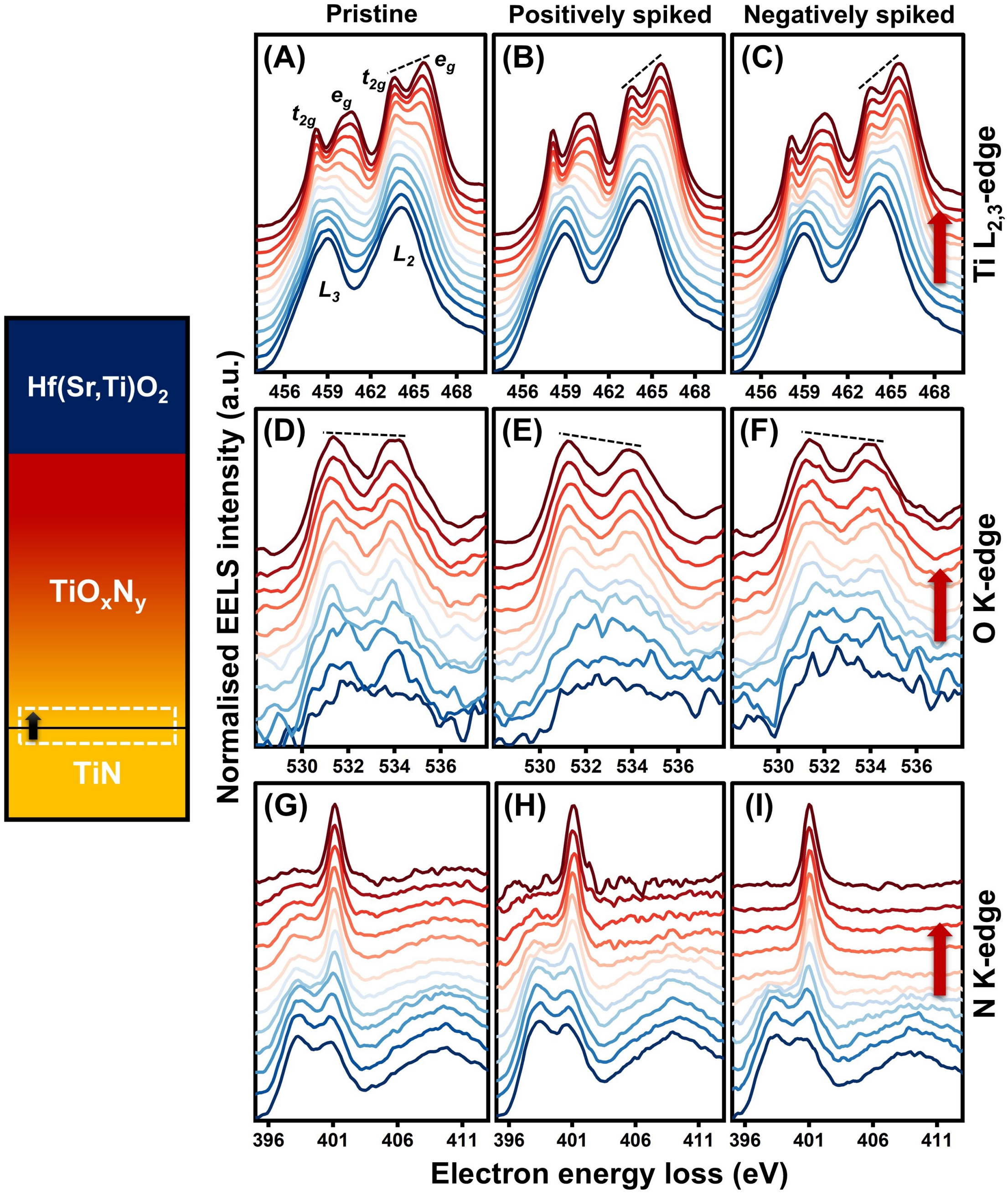

Fig. S7. EELS spectra. Ti-L, O-K, and N-K EELS spectra acquired from the TiO_xN_y/TiN interfaces in pristine state and after implementing positive and negative 1.0-V spikes. There is an ~1.0-nm interval between each pair of spectra.

Figure S8 shows the normalised Raman spectra obtained from *in-situ* Raman spectroscopy during applying 80 I-V voltage-sweeping cycles up to +1.0 V, plotted without any y-axis offset. Applying 80 voltage-sweeping cycles results in an increase of ΔA = 16% in the area under the peak appeared between 400 cm^{-1} and 480 cm^{-1} as well as an increase in the intensity of the TiN components, indicated by red arrows in Fig. S8(A). Figures S8(B) and S8(C) show the peaks appeared between 400 cm^{-1} and 480 cm^{-1} for the pristine and 80th-cycled states. They were deconvoluted to determine the area fractions under the Ti and (TiO_xN_y + HfO_2-based) components.

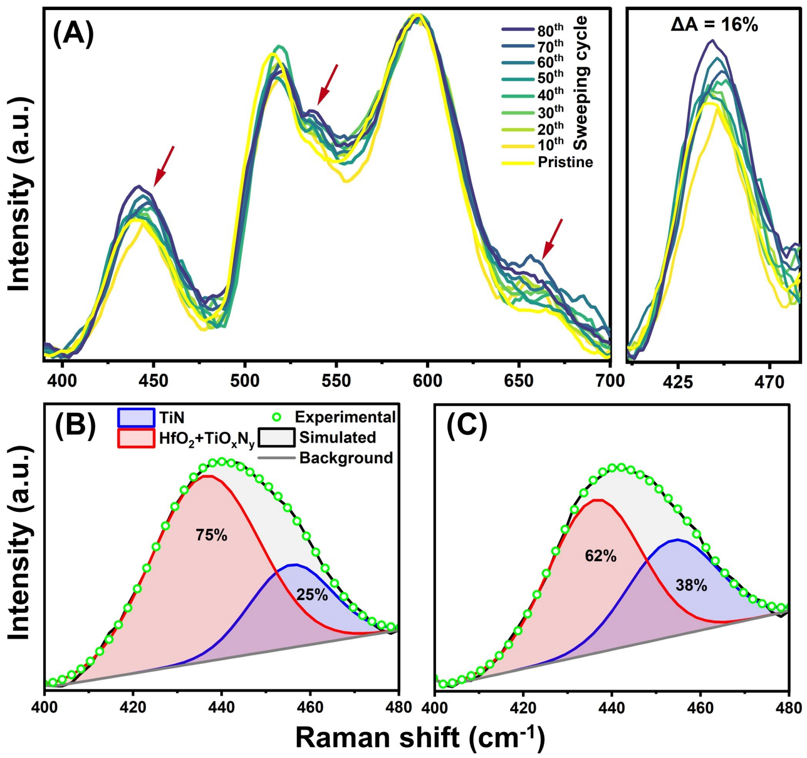

Fig. S8. Raman spectra. (A) Normalised Raman spectra obtained from *in-situ* Raman spectroscopy during applying 80 I-V voltage-sweeping cycles up to +1.0 V. Deconvoluted peaks appearing between 400 cm^{-1} and 480 cm^{-1} for the (B) pristine and (C) 80th-cycled states.

Figure S9 shows the depth-resolved, high-energy-resolution Hf 4f XPS core-level spectra as a function of depth *d*, acquired from the Mo/Hf(Sr,Ti)O_2/TiN devices in a pristine state and after implementing positive and negative 1.0-V spikes. To prevent damaging core levels by Ar-ion sputter-etching during depth profiling, ~16-nm-thick circular-shape Mo contacts were deposited on the Hf(Sr,Ti)O_2/TiN surface, without exposing the sample to air. The binding energies were calibrated against the Fermi edges. The spectra are plotted based on the depth from the Mo surface (d = 0 nm) towards Hf(Sr,Ti)O_2 in a stacked mode with different y-axis offsets.

For all three device states, the peak shapes at binding energies ≥ ~17.5 eV do not change as a function of *d*; however, there are shoulders appearing at the lower binding-energies of the Hf 4f spectra, indicating the presence of Hf with lower oxidation states (Hf^{n+} with n ≤ 3). The area under these shoulders increases up to d = ~16.5 nm but does not change for d > ~16.5 nm, which are sputter-etched regions entirely inside Hf(Sr,Ti)O_2. We previously demonstrated that Ar-ion sputter-etching during HfO_2 depth-resolved XPS measurements causes damages to the Hf 4f core levels that appear as low-binding-energy shoulders; however, the shape and area under these shoulders do not change as a function of depth *(85)*. Thus, this can be deduced that excluding the core-level-damage contributions, the shape of the Hf 4f spectra do not significantly change along the Hf(Sr,Ti)O_2 thickness for all three conditions.

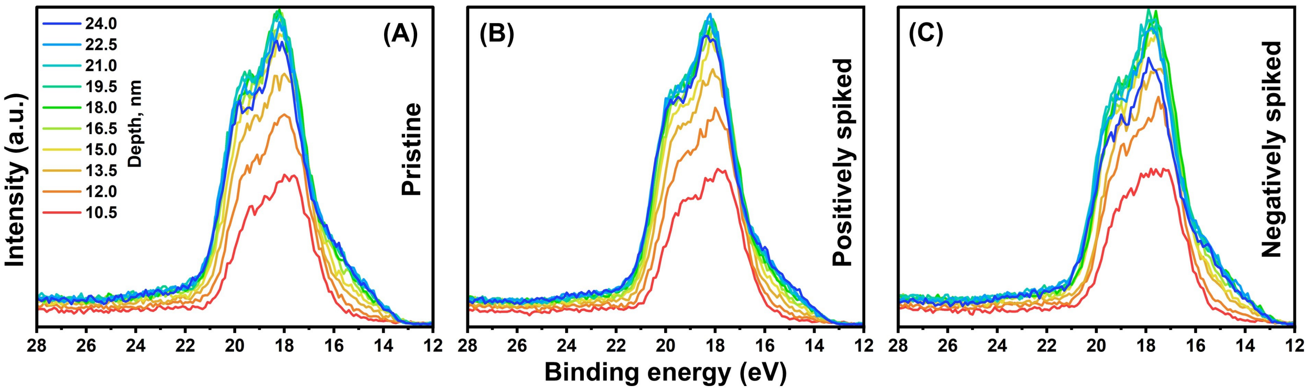

Fig. S9. Hf 4f XPS spectra. Depth-resolved, high-energy-resolution Hf 4f XPS core-level spectra as a function of depth *d*, acquired from the Mo/Hf(Sr,Ti)O_2/TiN/a-SiO_2/Si devices (A) in pristine state and after implementing (B) positive and (C) negative 1.0-V spikes.

Figure S10 shows the deconvoluted Hf spin-split $4f_{5/2}$-$4f_{7/2}$ doublet peaks of reference HfO_2 grown by our two-step sputtering design and Hf(Sr,Ti)O_2 at pristine, positively- and negatively-spiked conditions acquired at depths d = ~13.5 nm. The Hf 4f core-level spectra were deconvoluted after the Shirley-type background subtraction by maintaining the same $4f_{5/2}$-$4f_{7/2}$ binding-energy separations (1.6 eV), line shapes (Gaussian-Lorentzian), full-width-at-half-maximum values (1.6 eV), and $4f_{5/2}$:$4f_{7/2}$ area ratios (3:4), while peak areas and positions were changed.

Opposite to the Hf 4f spectrum of reference HfO_2 in Fig. S10(A) that was fitted well with a single pair of $4f_{5/2}$ and $4f_{7/2}$ components, associating to fully oxidised Hf (Hf^{4+}), the spectra of Hf(Sr,Ti)O_2 for all three conditions consist of additional doublets, Figs. S10(B)-S10(D). Since the as-deposited Hf(Sr,Ti)O_2 thin film is stoichiometric, the presence of extra Hf oxidation states can be mainly attributed to doping effects.

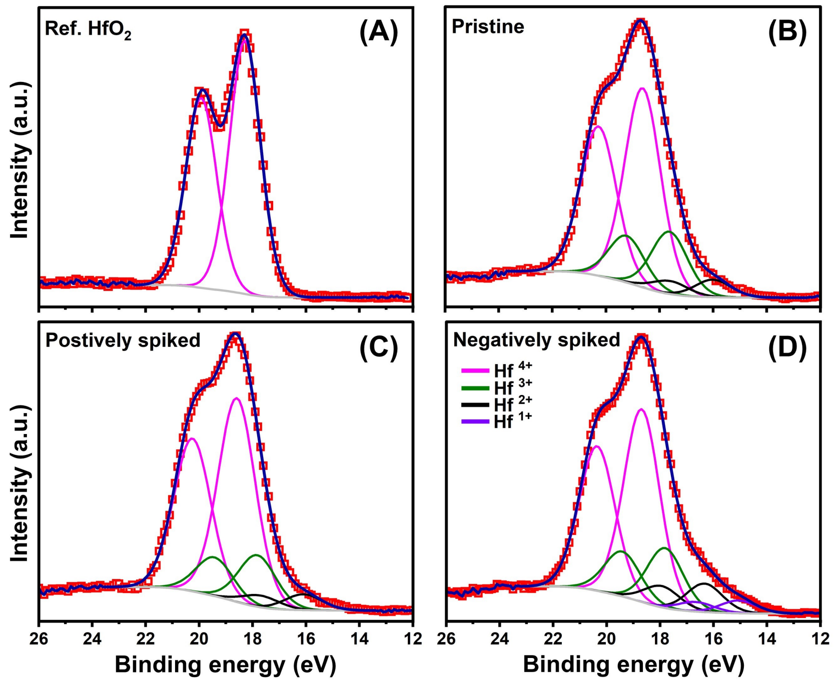

Fig. S10. Deconvoluted Hf 4f XPS spectra. Deconvoluted Hf spin-split $4f_{5/2}$-$4f_{7/2}$ doublet peaks of (A) reference HfO_2 and Hf(Sr,Ti)O_2 at (B) pristine, (C) positively- and (D) negatively-spiked states acquired at *d* = ~13.5 nm.

Similar to the Hf 4f spectra, the same changes can be expected in O 1s core-level spectra. Yet, the normalised O 1s spectra acquired from the pristine and spiked Mo/Hf(Sr,Ti)O_2/TiN devices at depths *d* = ~13.5 nm, plotted in Fig. S11, consist of peaks with similar widths and shapes, with no chemical shift and visible peak splitting. This is mainly due to the fact that the peaks of O 1s with different oxidation states appear very close, and the variations in the fractions of their oxidation states are not adequately high enough to change the peak shapes *(85)*.

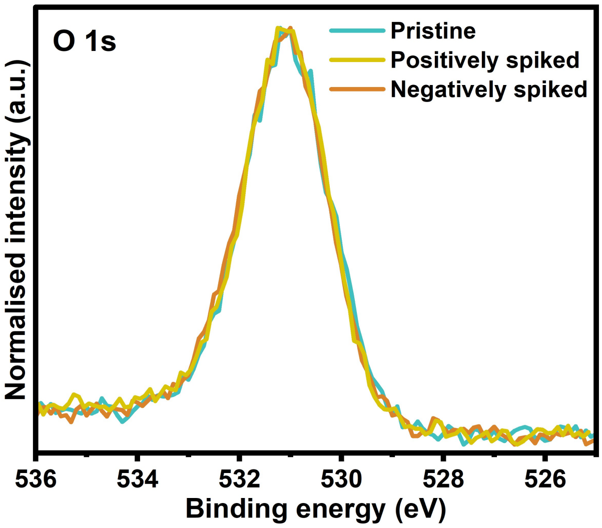

Fig. S11. O 1s XPS spectra. Normalised high-energy-resolution O 1s XPS core-level spectra acquired from the pristine and spiked Mo/Hf(Sr,Ti)O_2/TiN/a-SiO_2/Si devices at depths *d* = ~13.5 nm.

Figure S12 compares the normalised high-energy-resolution Mo 3d XPS core-level spectra acquired from the pristine and spiked Mo/Hf(Sr,Ti)O_2/TiN devices at depths *d* = ~10.5, ~14.5, and ~15.5 nm. For all three conditions, the Mo $3d_{5/2}$ peaks acquired from *d* = ~10.5, ~14.5, and ~15.5 nm appear at ~229.7 eV. This indicates that there is no considerable chemical shift upon applying

training voltage pulses at different depths. In addition, there is no significant peak broadening. Overall, the Mo 3d core-level spectra do not show any detectable chemical changes at the Mo/Hf(Sr,Ti)O_2 interface upon applying training spikes.

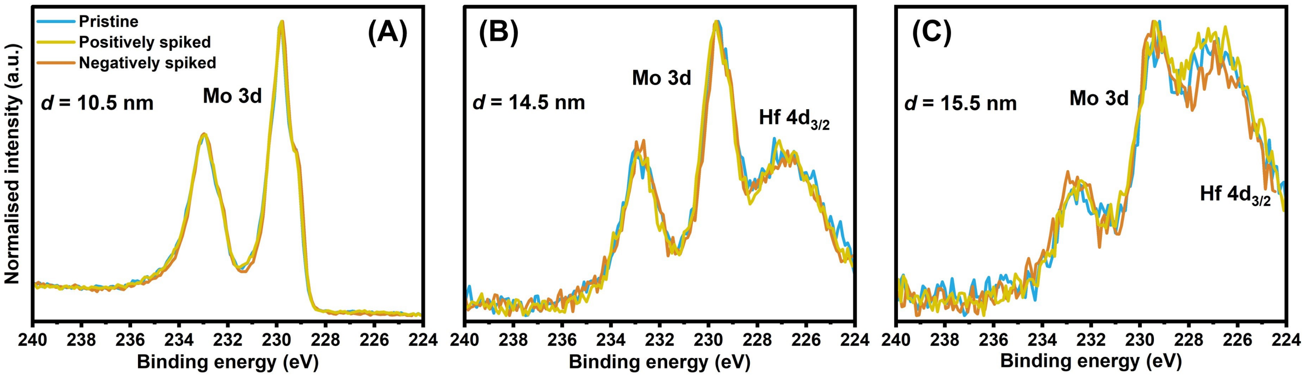

Fig. S12. Mo 3d XPS spectra. Normalised high-energy-resolution Mo 3d XPS core-level spectra acquired from the pristine and spiked Mo/Hf(Sr,Ti)O_2/TiN/a-SiO_2/Si devices at depths *d* = (A) ~10.5 nm, (B) ~14.5 nm, and (C) ~15.5 nm.

Figure S13 shows the I-V curves of Au/Cr/TiO_xN_y/TiN/a-SiO_2/Si devices for which Hf(Sr,Ti)O_2 was firstly sputter-etched from Hf(Sr,Ti)O_2/TiO_xN_y/TiN in the XPS chamber using 200-eV Ar ions, and then 100-nm-thick Au/2-nm-thick Cr contacts were deposited on TiO_xN_y. The I-V curves do not show any hysteretic loops. Furthermore, symmetric I-V characteristic can be seen when a bias was scanned between negative and positive voltages. This observation rules out the possible impact of the TiO_xN_y/TiN region on our devices' switching mechanism.

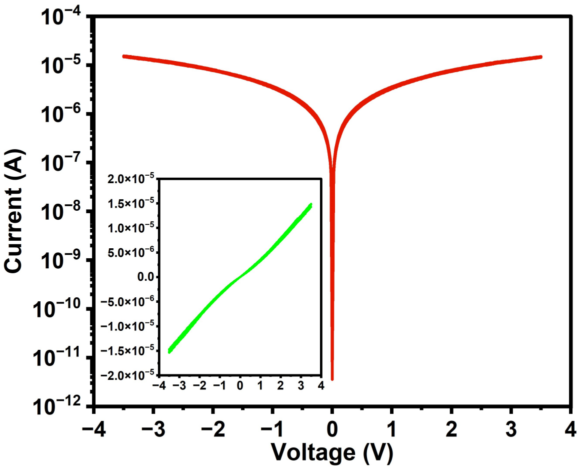

Fig. S13. I-V cycles. 30 I-V sweeping cycles of Au/Cr/TiO_xN_y/TiN devices (d_{TE} = 50 μm).

Figure S14 compares 30 I-V sweeping cycles of Hf(Sr,Ti)O_2/TiN devices with Ti and Au/Cr top electrodes (d_{TE} = 50 μm) to assess the effect of electrode materials with different work functions ($\emptyset_{\mathrm{Ti}}$ = ~4.3 eV and $\emptyset_{\mathrm{Au}}$ = ~5.3 eV *(86)*) and oxygen affinities ($\Delta H^{\circ}_{f,\mathrm{TiO_2}}$ = -945 kJ/mol *(87)*, $\Delta H^{\circ}_{f,\mathrm{MoO_2}}$ = -593 kJ/mol *(88)*, and $\Delta H^{\circ}_{f,Au}$ = not available). The UPS-determined work functions of TiN and Mo are $\emptyset_{\mathrm{TiN}}$ = ~4.0 eV and $\emptyset_{\mathrm{Mo}}$ = ~4.6 eV, respectively (Fig. S15). The shapes of the I-V curves appear similar to those of Mo/Hf(Sr,Ti)O_2/TiN devices shown in Fig. 1 in the main paper, confirming that the top-electrode material does not play a significant role in the switching mechanism.

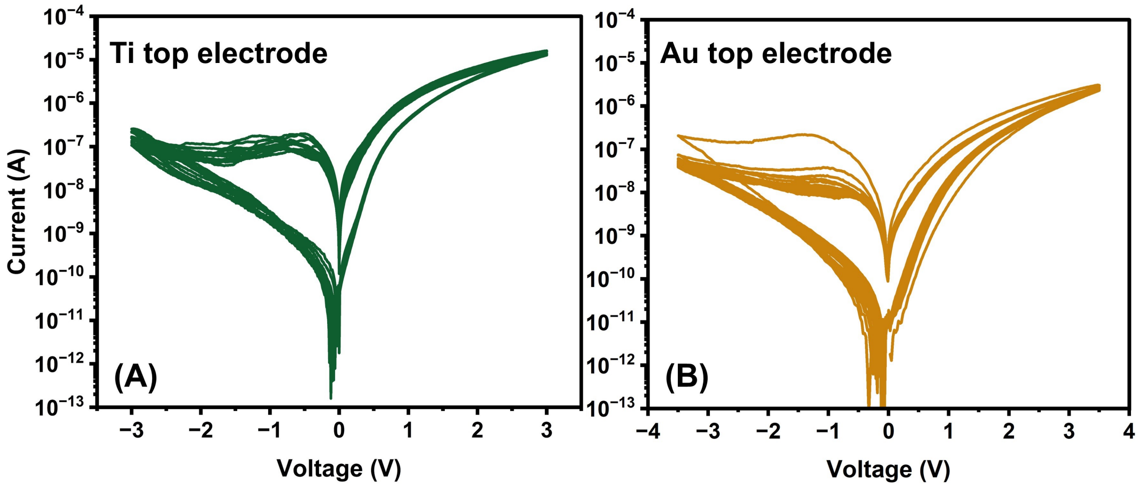

Fig. S14. Top electrode role in switching performance. 30 I-V sweeping cycles of $Hf(Sr,Ti)O_2/TiN/a\text{-}SiO_2/Si$ devices with (A) Ti and (B) Cr/Au top electrodes (d_{TE} = 50 µm).

Figure S15 shows the UPS spectra of the TiN and Mo thin films. Having the samples grounded, work functions (∅) were determined following Einstein equation: $\emptyset = \hbar\omega - E_{cutoff}$ by identifying secondary electron cut-off energies, Fig. S15(A), and calibrating Fermi-energy edges at 0 eV, see Fig. S15(B). The UPS-obtained work functions of TiN and Mo are $\emptyset_{\mathrm{TiN}}$ = ~4.0 eV and $\emptyset_{\mathrm{Mo}}$ = ~4.6 eV, respectively.

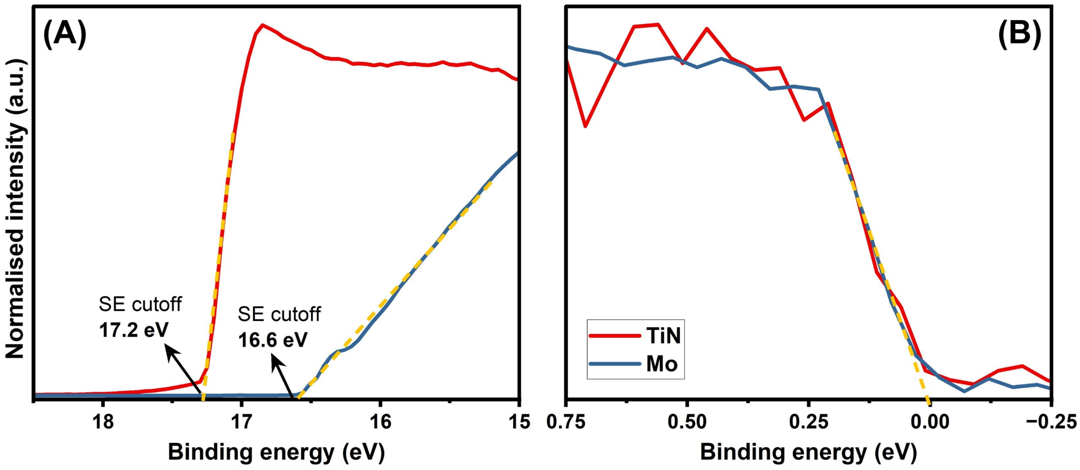

Fig. S15. UPS spectra. UPS spectra of sputter-deposited TiN and Mo thin films.

Figure S16 shows the I-V sweeping cycles of $Mo/Hf(Sr,Ti)O_2$ devices with TiN and electrically conductive *p*-type $La_{0.7}Sr_{0.3}MnO_3$ (LSMO) bottom electrodes (d_{TE} = 50 µm). While the TiN electrode was deposited on $a\text{-}SiO_2/Si$, LSMO was grown on a single-crystal $SrTiO_3$ substrate. In both cases, applying a positive voltage exponentially increases the current, changing the resistance state of the devices from HRS to LRS. The current of the $Mo/Hf(Sr,Ti)O_2/TiN$ device at +3.5 V is ~2000 times lower than that of $Mo/Hf(Sr,Ti)O_2/LSMO$. However, these devices show significantly different I-V

curves upon negative-voltage applications (in terms of both shape and current level). Over the entire negative-voltage range, the Mo/Hf(Sr,Ti)O_2/TiN device has a substantially lower current compared to Mo/Hf(Sr,Ti)O_2/LSMO. At -1.0 V and LRS, the absolute current is ~7.5×10^{-8} A for Mo/Hf(Sr,Ti)O_2/TiN, while it is ~1.0×10^{-3} A for Mo/Hf(Sr,Ti)O_2/LSMO. In addition, the rectification ratio, defined as the ratio of currents at the same voltages that have different polarities, is ~155 for Mo/Hf(Sr,Ti)O_2/TiN and ~2.6 for Mo/Hf(Sr,Ti)O_2/LSMO at ±3.0 V and HRS. We also determined the ideality factor (IF) of the Mo/Hf(Sr,Ti)O_2/TiN device from its I-V curve plotted in Fig. S16(C) using the

$$\mathrm{IF} = \frac{\mathrm{q}}{\mathrm{k_B.T}} \frac{d\mathrm{V}}{d(\ln \mathrm{I})}$$ equation *(89)*,

where q is the electron charge (1.6×10^{-19} C), k_B is the Boltzmann constant (1.38×10^{-23} J/K), T is the absolute temperature (~300 K, here), and $d\ln(I)/dV$ is the slope of the linear region of the ln(I)-V plot at forward bias. Accordingly, the ideality factor is estimated to be ~6.4, which is significantly higher than that of ideal Schottky barrier diodes (n = 1) *(90)*. The built-in potential is V_{bi} = ~1.0 V, see the inset in Fig. S16(C).

Overall, while the Mo/Hf(Sr,Ti)O_2/LSMO devices with a metal/oxide-like bottom interface follow interfacial Schottky-emission-dominated switching mechanism, the Mo/Hf(Sr,Ti)O_2/TiN devices with a significant increase in resistance during the reverse voltage sweeps and pronounced rectification ratio show typical electrical characteristics of a *p-n* like diode.

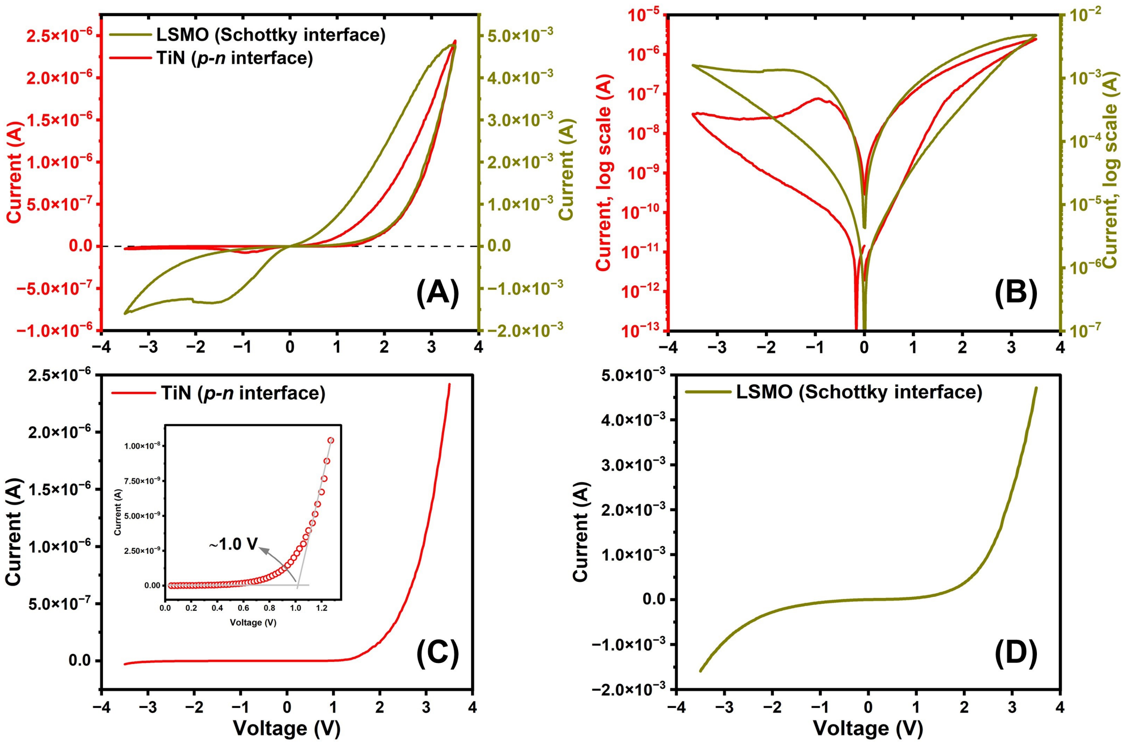

Fig. S16. Bottom electrode role in switching performance. I-V sweeping cycles of Mo/Hf(Sr,Ti)O_2 devices with TiN and LSMO bottom electrodes (d_{TE} = 50 μm), plotted in (A) linear and (B) logarithmic scales. I-V characteristic of Mo/Hf(Sr,Ti)O_2 devices with (C) TiN and (D) LSMO electrodes.

Figure S17 shows the results of the Hall effect measurement as a function of the magnetic field. There is an upward trend (positive Hall coefficient) by increasing the magnetic field that indicates the *p*-type nature of Hf(Sr,Ti)O_2. The hole concentration (*N*) was estimated by using the

$$N = \frac{\mathrm{I.B}}{\mathrm{V_H.q.t}}$$ equation *(91, 92)*,

where V_H/B is the slope of the V_H-B plot, I is the applied current, t is the thickness of Hf(Sr,Ti)O_2, and q is the electron charge. The hole concentration is estimated to be *N* = ~8.4 × 10^{17} cm^{-3}.

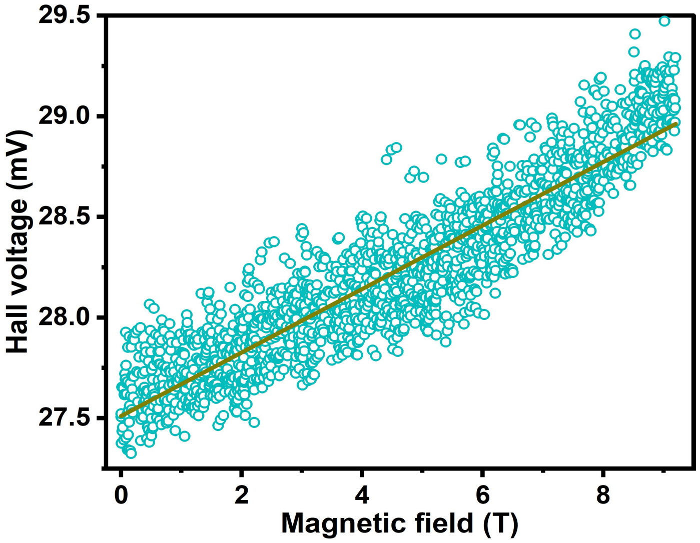

Fig. S17. Hall effect data. Hall effect voltage changes of the sputter-deposited stoichiometric Hf(Sr,Ti)O2 oxide layer as a function of the magnetic field measured at 400 K.

Figures S18(A) and S18(B) show the UV-vis transmittance spectra and Tauc plots of the stoichiometric HfO_2 and Hf(Sr,Ti)O_2 layers grown on quartz substrates. Over a wide range of wavelengths, both layers have transmittance > ~90 %. Optical bandgap energies estimated from the Tauc plots of HfO_2 and Hf(Sr,Ti)O_2 are E_g = ~5.7 and ~4.5 eV, respectively. These values are in good agreement with those obtained from our calculated optical absorption spectra shown in Fig. S18(C) that are ~6.1 eV for HfO_2 and ~4.7 eV for Hf(Sr,Ti)O_2.

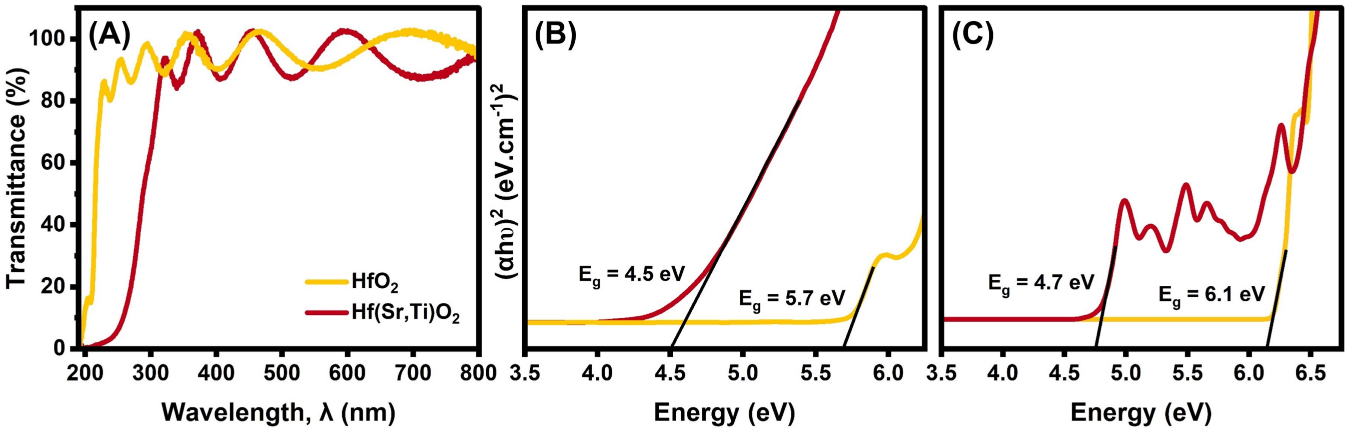

Fig. S18. UV-vis transmittance spectra and Tauc plots. (A) UV-vis transmittance spectra, (B) Tauc plots, and (C) calculated absorption spectra of reference HfO_2 and $Hf(Sr,Ti)O_2$ layers sputter-deposited on quartz substrates.

Figure S19 shows the partial and total densities of states (DOS) calculated at the HSE06 level for HfO_2 and $Hf(Sr,Ti)O_2$ under O-rich environments. The DOS calculations were carried out using two different spin channels: spin up and spin down. Figure S19 represents the spin-up DOS data. For both material systems, while Hf *d* orbitals have the dominant states in the conduction band, the valance band is dominated by the O states (*p* orbitals). In addition, the bandgap values are estimated to be ~6.0 eV for HfO_2 and ~4.5 eV for $Hf(Sr,Ti)O_2$, which are highly comparable with the optical bandgap values obtained from the UV-vis measurements and calculated absorption spectra. As shown in Fig. S19(B), the bandgap reduction in $Hf(Sr,Ti)O_2$ mainly originates from the presence of Ti dopants (electrons from Ti *d* orbitals). We note that the spin-down DOS data indicates the presence of unoccupied oxygen gap states caused by Sr dopants.

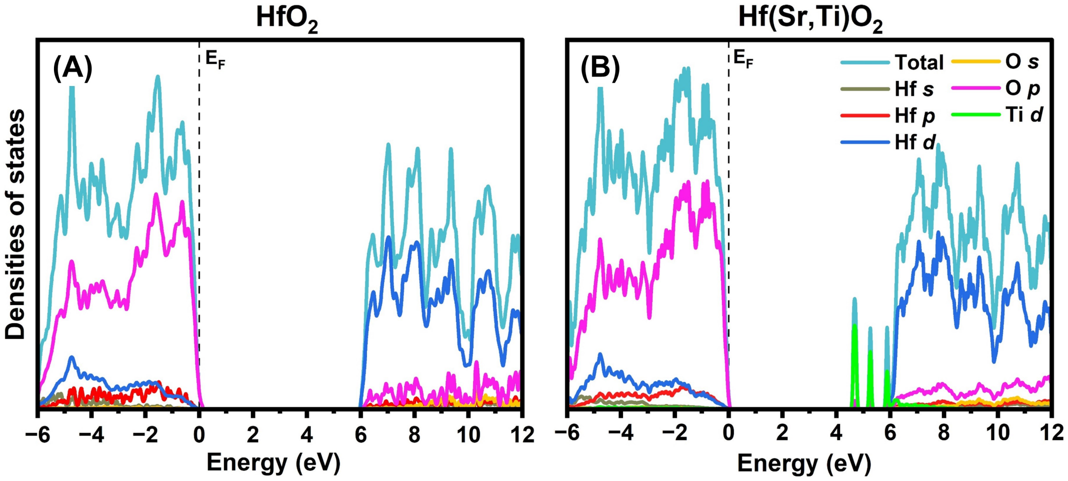

Fig. S19. Calculated DOS data. Partial and total density of states (DOS) calculated from the spin-up channel for stoichiometric (A) HfO_2 and (B) Hf(Sr,Ti)O_2. The actual Sr states cannot be seen because they are well below the valence band (as Sr is in the 2+ state).

Figure S20 compares the HRS/LRS ratios of our devices with ~40 memristors with *p-n* heterointerfaces. These memristors that are made of different materials, including *n*-type TiO_2, exhibit interfacial or hybrid (filamentary and interfacial) resistive switching (Details are given in table SII). Almost all these memristors did not demonstrate any reported neuromorphic performance. Our devices are notable by their large memory window among the interfacial *p-n* heterointerface memristors.

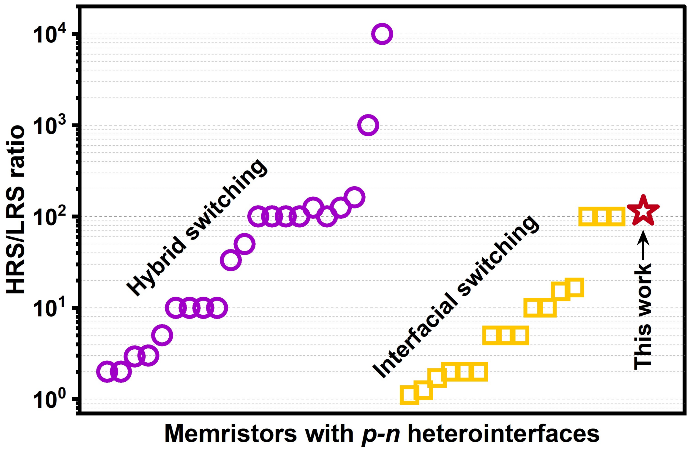

Fig. S20. Resistance ratios for various *p-n* memristors. HRS/LRS ratios of devices in this work compared with ~40 memristors with *p-n* heterointerfaces (Details are given in table SII).

Figure S21(A) schematically illustrates the isolated energy band diagrams of TiN, TiO_2 (here, O-rich TiO_xN_y), Hf(Sr,Ti)O_2, and Mo. Work function (∅), electron affinity (χ), and bandgap (E_g) values are ∅ = ~4.5 eV *(93-95)*, χ = ~4.0 eV *(96-98)*, and E_g = ~3.2 eV *(99-101)* for O-rich TiO_xN_y, and ∅ = ~5.6 eV (UPS determined), χ = ~1.8 eV *(102-104)* (since the χ value for Hf(Sr,Ti)O_2 is unknown, we

use that of HfO_2. Similar approximation was made for O-deficient sputter-deposited HfO_2 with E_g = ~4.5 eV in reference *(104)*, and E_g = ~4.5 eV (UV-vis determined) for $Hf(Sr,Ti)O_2$. As illustrated in Fig. S21(A), the Fermi energy level lies close to the valence band maximum (VBM) for *p*-type $Hf(Sr,Ti)O_2$, while it is near the conduction band minimum (CBM) for *n*-type O-rich TiO_xN_y. The energy band diagram for the $Hf(Sr,Ti)O_2$/O-rich TiO_xN_y heterostructure at the pristine state (thermal equilibrium) is schematically shown in Fig. S21(B). The work function difference between O-rich TiO_xN_y and $Hf(Sr,Ti)O_2$ generates a built-in potential V_{bi} of ~1.1 V (theoretical value obtained from the band diagram). This value is in the range of V_{bi} determined from our current-voltage and capacitance-voltage measurements, ~1.0 V and ~1.2 V, respectively. Since the TiO_xN_y layer is more defective than stochiometric $Hf(Sr,Ti)O_2$, it has significantly higher carrier concentration. Thus, the built-in potential is expected to largely form inside the $Hf(Sr,Ti)O_2$ thin film that causes a wider depletion region in this *p*-type layer: forming a space-charge region asymmetrically extended inside $Hf(Sr,Ti)O_2$.

We estimated the width of the depletion region (W_d) inside $Hf(Sr,Ti)O_2$ using the

$$W_d = \sqrt{\frac{2\varepsilon_o\varepsilon_r V_{bi}}{qN}}$$ equation *(90)*,

where ε_o is the vacuum permittivity (8.854×10^{-14} F/cm), ε_r is the oxide dielectric permittivity (we use that of Sr-doped HfO_2, ~30 *(105, 106)*, which is also close to that of Ti-doped HfO_2 *(107)*), q is the electron charge (1.6×10^{-19} C), and *N* is the carrier concentration. Depending on the O deficiency and dopant concentrations, three different regimes of low (~1.0×10^{17} cm^{-3} *(108)*), medium (~1.0×10^{19} cm^{-3} *(104, 109)*), and high (~1.0×10^{21} cm^{-3} *(70, 110, 111)*) charrier concentrations can be considered for HfO_2 with close to stoichiometry compositions. Thus, W_d can range from ~190 to ~19 to ~1.9 nm. Based on our calculations carried out on $Hf(Sr,Ti)O_2$ with an ideal crystal structure at *equilibrium* conditions, using the py-sc-fermi package *(83)*, the majority charge carriers in

stoichiometric Hf(Sr,Ti)O_2 are holes with an estimated concentration of ~2.3 × 10^{18} cm^{-3} that further supports the *p*-type nature of Hf(Sr,Ti)O_2. Overall, the concentration of holes is significantly less than the electron concentration in *n*-type, O-rich TiO_xN_y (~1.2 × 10^{20} cm^{-3}, determined from our capacitance-voltage measurements and is in the range reported for typical *n*-type TiO_2 *(112-114)*). This confirms our above speculation that the space-charge region largely extends into Hf(Sr,Ti)O_2. Our Hall-measured and calculated hole concentrations lie between the low and medium N regimes, resulting in a depletion region that its width can exceed the Hf(Sr,Ti)O_2 thickness.

The key point here is the formation of a depletion region that is asymmetrically extended inside Hf(Sr,Ti)O_2. This unique electronic structure can explain the low conductance values observed in our devices and their asymmetric trends that vary significantly with the polarity of the presynaptic spikes, see Figs. 2(A)-(C) in the main paper.

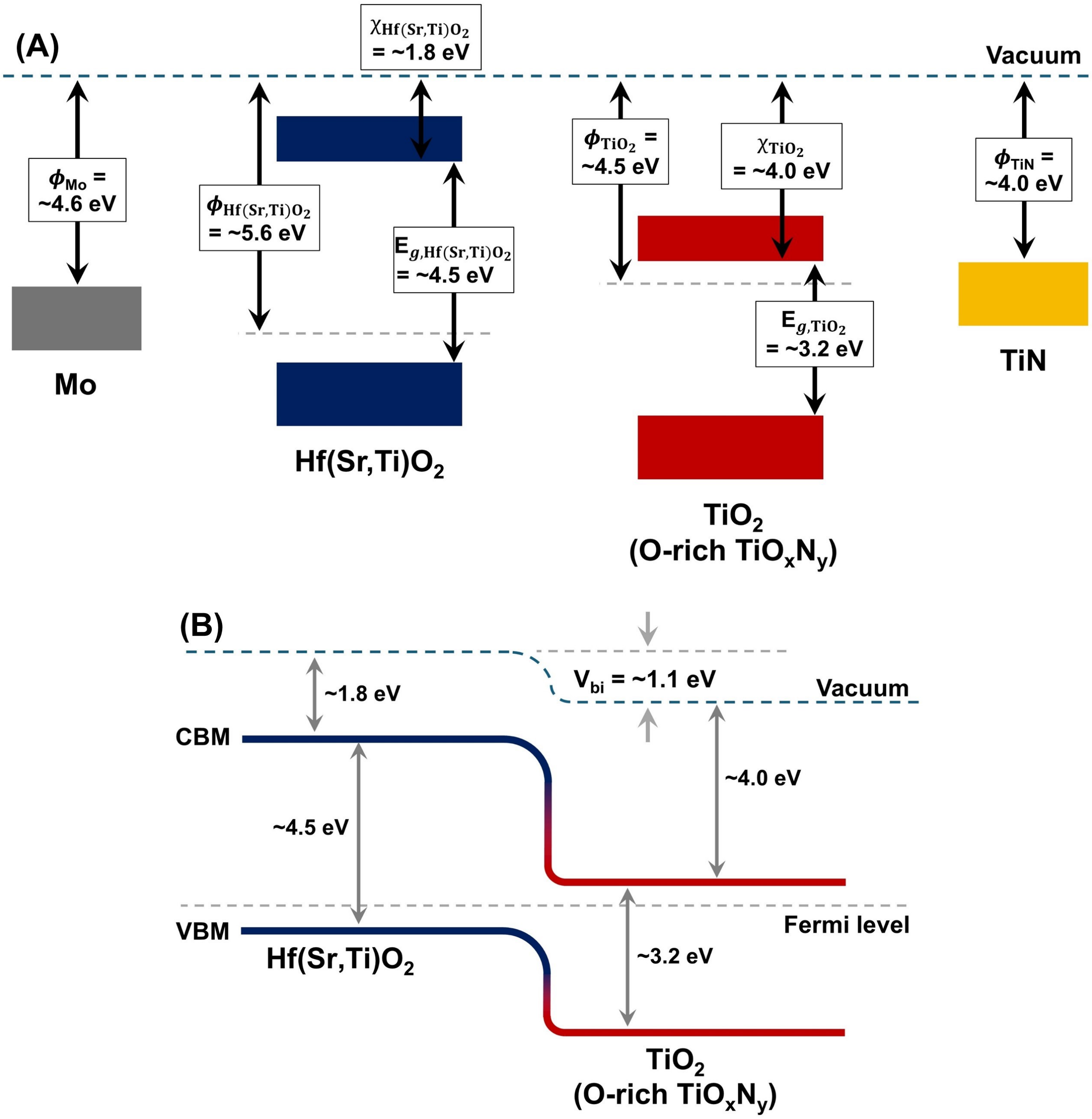

Fig. S21. Energy band diagrams. (A) Isolated energy band diagrams of TiN, TiO_2 (here, O-rich TiO_xN_y), Hf(Sr,Ti)O_2, and Mo. (B) Energy band diagram of TiO_2(here, O-rich TiO_xN_y)/Hf(Sr,Ti)O_2 heterostructure at the pristine state (thermal equilibrium).

Figure S22 shows the 2nd and 1100th I-V curves of a typical Mo/Hf(Sr,Ti)O_2/TiN device together with their double logarithmic I-V plots. Among the various mechanisms commonly employed to predict the conduction behaviour of memristive devices *(115)*, the space-charge limited current model aligns most closely with our experimental I-V data, particularly as the oxide layer in these devices is relatively thick. The double logarithmic plots obtained from the positive-voltage sweeping segments of the I-V curves, shown in Figs. S22(B) and S22(E), consist of three different regimes: Ohmic ($I \sim V$) regions at low voltages, trap-filling ($I \sim V^2$) regions at intermediate voltages, and trap-filled regions with rapid current increases ($I \sim V^n$, with $n > 2$) at higher voltages. For the trap-filled regime, the value of n depends on the trap distribution in the forbidden energy gap *(116)*. At the Ohmic regions, the concentration of thermally-generated carriers exceeds injected charge carriers. As the concentration of the injected carriers increases sufficiently, they are caught by the trapping sites induced by structural and chemical defects in the lattice of the switching layer. The device conduction at this trap-filling regime is higher than the Ohmic region. After all traps are occupied, the injected charge carriers can freely transport inside the oxide, resulting in a rapid current increase (the trap-filled regime) *(117)*. While the double logarithmic plots from the positive I-V sections have three distinct regimes, those of the negative I-V sections, shown in Figs. S22(C) and S22(F), consist of only trap-filling and trap-filled regimes. Worth mentioning that this conduction mechanism was reported for several memristors with *p-n* heterointerfaces *(116, 118-120)*; however, our devices are distinguished by their excellent uniformity, e.g., the remarkable cycle-to-cycle uniformity is confirmed by the slopes of their double logarithmic plots that reveal negligible variation even after subjecting the device to 1100 cycles.

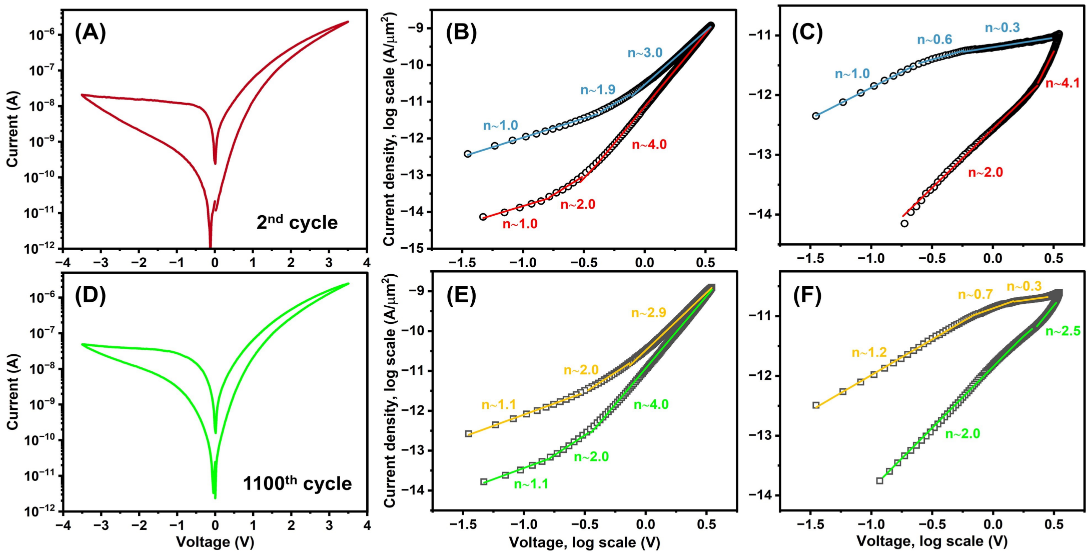

Fig. S22. I-V curves and their double logarithmic I-V plots. (A) 2nd and (D) 1100th I-V curves of a typical Mo/Hf(Sr,Ti)O_2/TiN/a-SiO_2/Si device (d_{TE} = 50 μm). The device was subjected to 1100 consecutive I-V cycles. Double logarithmic plots of the 2nd I-V cycle in (B) positive- and (C) negative-voltage sweeping regions. Double logarithmic plots of the 1100th I-V cycle in (E) positive- and (F) negative-voltage sweeping regions.

Figure S23 shows the SEM image from the top-view device geometry and a typical Mo top electrode used in the electrical measurements.

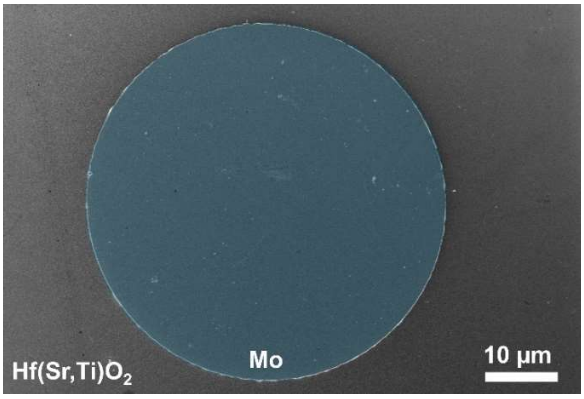

Fig. S23. Device geometry. SEM image showing the top-view device geometry and a typical Mo top electrode used in the electrical measurements. The Mo electrode is coloured.

$Hf(Sr,Ti)O_2$ thickness optimisation

The results presented in this work are obtained from optimised devices identified through systematic optimisation of deposition parameters, including oxide thickness, controlled by deposition time. Devices fabricated with different thicknesses exhibited markedly different switching behaviours. (i) Short deposition times. These devices mainly exhibited filamentary resistive switching, with poor switching performance, large variability, and no analogue modulation. The behaviour is consistent with insufficient film thickness to support the extended depletion region required for interface-dominated switching. Instead, oxygen-vacancy filaments tend to form through the full $Hf(Sr,Ti)O_2$ thickness. (ii) Long deposition times. Excessively thick layers led to weak or unstable hysteresis and incomplete switching. In these devices, the electric field across the $Hf(Sr,Ti)O_2$ layer is too small to produce effective modulation of the *p-n* depletion width at reasonable voltages; consequently, neither analogue evolution nor stable SET/RESET transitions are observed. (iii) Optimised deposition time. At the optimised growth time, the devices exhibited the behaviour reported in the manuscript: forming-free, ultra-low-current switching, high uniformity, and smooth analogue conductance evolution. At this growth condition, the electric field and the band-bending at the *p*-$Hf(Sr,Ti)O_2$/*n*-TiO_xN_y heterointerface enable controlled modulation of the depletion region, producing the desired non-filamentary *p-n* junction-dominated behaviour. These observations are consistent with earlier studies on interface-type memristors, where a critical oxide thickness is needed for stable depletion-region modulation and analogue synaptic behaviour.

Table S1. Neuromorphic characteristics reported for HfO_2-based memristive devices.

Device structure	Switching oxide growth technique and mechanism	Spike programming scheme	Postsynaptic conductance range (S)	Number of synaptic weights	Weight change symmetry / linearity	Other features	Ref.
Mo /Hf(Sr,Ti)O_2/ TiN/SiO_2/Si	Sputtering / Interfacial	Identical +1.0V / -1.0V dt = 1 ms	2.5×10^{-9} - 1.4×10^{-7}	6000 - 6000	Semi-symmetric / semi-linear	No need of forming and current compliance	*This work*
TiN/Ti/ $HfTaO_x$/TiN/ TaN/SiO_2/Si	ALD / Filamentary	Identical +2.5V / -2.4V dt = 0.1 ms	3.8 - 9.5×10^{-6}	90 - 60	Semi-symmetric / semi-linear	Need forming and current compliance	*121*
Ta/HfO_x/Pt/ Ti/SiO_2/Si	ALD / Filamentary	Non-identical +0.75-1.0V / -1.05-1.17 V dt = 0.1 µs	0.4 - 1.8×10^{-3}	26 - 13	Asymmetric / non-linear	Need forming and current compliance	*122*
Ti/TaO_x/HfO_2 /Pt/Ti/SiO_2/Si	ALD / Interfacial	NA	2.8 - 4×10^{-4}	50 - 50	Semi-symmetric / semi-linear	NA*	*123*
Ta/HfO_x/ Pt/Ti/SiO_2/Si	ALD / Filamentary	NA	0.1 - 1.2×10^{-3}	100 - 100	Symmetric / linear	1T1R configuration	*124*
TiN/HfO_2/TiN /Si	ALD / Filamentary	Identical -0.7V / +0.9V dt = 0.1 ms	0.5 - 1.5×10^{-3}	500 - 500	Asymmetric / non-linear	Need forming and current compliance	*125*
Au/HfO_x/ HfO_2/ Pt/SiO_2/Si	Sputtering / Filamentary	Identical +0.15V / -0.15V dt = 50 µs	1 - 5×10^{-4}	50 - 50	Asymmetric / non-linear	Need forming and current compliance	*126*
Pt/TiO_x/HfO_2/ Ti/Si	ALD / Filamentary	Identical -1.2V / +1.4V dt = 50 ns	0.5 - 2.5×10^{-3}	100 - 100	Asymmetric / non-linear	Need forming and current compliance	*127*
Ti/HfO_2/ Pt/SiO_2/Si	ALD / Filamentary	Identical +1.5V / -2V dt = 1 µs	6.8 - 8.0×10^{-4}	100 - 100	Asymmetric / semi-linear	Forming-free / Need current compliance	*128*
TiN/Ti/HfO_2/ Pt/SiO_2/Si	ALD / Filamentary	Non-identical -1.2-2.0V / +1.4V dt = 1 µs	10^{-7} - 10^{-4}	81 - 1	Asymmetric / non-linear	Need forming and current compliance	*129*
Pt/HfO_2/TiO_x/ Pt/Ti/Si	ALD / Filamentary	Identical -0.6V / +0.8V dt = 1 µs	1.5 - 20×10^{-5}	50 - 50	Asymmetric / non-linear	Need forming and current compliance	*130*
TiN/Ti/Al_2O_3/ HfO_2/TiN /SiO_2/Si	ALD / Filamentary	Identical +3.0V / -1.6V dt = 10 µs	0.3 - 1.0×10^{-7}	32 - 32	Asymmetric / non-linear	Need forming and	*131*

						current compliance	
$TiN/Ti/Al_2O_3/HfO_2/TiN/SiO_2/Si$	ALD / Filamentary	Identical +4.8V / -3.0V dt = 10 μs	$0.5 - 1.3 \times 10^{-7}$	64 - 64	Symmetric / linear	Need forming and current compliance	*131*
$Pt/HfO_x/Pt/Ti/SiO_2/Si$	Sputtering / Filamentary	Non-identical +0.7-1.2V	NA	18 - 0	Asymmetric / non-linear	Need forming and current compliance	*132*
$Pt/HfO_x/Ti/SiO_2/Si$	Sputtering / Filamentary	Identical +0.5V / -0.7V dt = 0.1 μs	$0.2 - 1.2 \times 10^{-3}$	100 - 100	Asymmetric / non-linear	Need forming and current compliance	*133*
$W/HfO_x/TiN/SiO_2/Si$	ALD / Interfacial	Non-identical +0.5-6.5V	$2 - 12.5 \times 10^{-5}$	7 - 0	NA	NA	*134*
$TiN/Ti/HfO_x/W/SiO_2/Si$	ALD / Filamentary	NA	0.01 - 0.0001	50 - 50	symmetric / semi-linear	Need forming and current compliance	*135*
$Ni/HfO_x/Al_2O_3/n\text{-}Si$	ALD / Interfacial	Identical +8.0V / -5.0V dt = 0.1 ms	$0.4 - 0.7 \times 10^{-7}$	50 - 50	Symmetric / linear	Need forming	*136*
$Pt/HfO_x/HfO_2/TiN/Si$	ALD / Filamentary	Identical -0.8V / +2.0V dt = 1 μs / 2 μs	$0.6 - 1.5 \times 10^{-3}$	40 - 40	Semi-symmetric / non-linear	Need forming and current compliance	*137*
$TaN/Al_2O_3/HfO_2/ITO/Glass$	ALD / Filamentary	Identical -1.0V / +1.0V dt = 1 μs	$10^{-5} - 10^{-4}$	50 - 50	Asymmetric / non-linear	Need forming and current compliance	*138*
$Ag/HfO_x/Pt/Ti/SiO_2/Si$	ALD / Filamentary	Identical +0.5V / -0.5V dt = 0.5 ms	$1 - 12 \times 10^{-3}$	30 - 30	NA	Need forming and current compliance	*139*
$Pt/HfAlO_x/TiN/SiO_2/Si$	ALD / Filamentary	Identical +0.9V / -1.6V dt = 0.1 ms	$1.6 - 3.0 \times 10^{-3}$	50 - 50	Asymmetric / semi-linear	Need forming and current compliance	*140*
$Cu/HfO_2/p\text{-}Si$	ALD / Interfacial	Identical -12.0V / +10.0V dt = 10 ms	$4.8 - 5.1 \times 10^{-3}$	40 - 40	Asymmetric / non-linear	NA	*141*
$Pd/HfO_2/WO_x/Si$	ALD / Interfacial	Identical +3V / -3.5V dt = 3 μs	$0.5 - 1.0 \times 10^{-7}$	200 - 200	Semi-symmetric / semi-linear	NA	*142*
$ITO/HfAlO_x/Pt/HfAlO_x/ITO/SiO_2/Glass$	ALD / Filamentary	Non-identical +0.4-1.18V / -0.6-1.38V dt = 0.1 ms	$1.0 - 2.5 \times 10^{-4}$	50 - 50	Asymmetric / non-linear	Need forming and current compliance	*143*

Ti/HfO_x/TiN /SiO_2/Si	ALD / Filamentary	Identical +3.0V / -3.0V dt = 1 s	2 - 40 × 10^{-6}	100 - 100	Asymmetric / non-linear	Need forming and current compliance	*144*
TiN/HfO_x/Al_2O_3/ITO/Glass	ALD / Filamentary	Non-identical -1.5V / +1.5V dt = 10-1000 µs	1.0 - 2.0 × 10^{-3}	50 - 50	Symmetric / linear	Need forming and current compliance	*145*
TiN/HfO_2/Al_2O_3/Pt/Ti/ SiO_2/Si	ALD / Filamentary	Identical +0.5V / -1.0V dt = 0.1 µs	0.1 - 3.8 × 10^{-6}	50 - 50	Asymmetric / semi-linear	Need forming and current compliance	*146*
Pt/Al_2O_3/ HfO_2/$HfAlO_x$/ TiN/SiO_2/Si	ALD / Filamentary	Identical -0.9V / +1.0V dt = 0.5 ms	3.2 - 4.2 × 10^{-3}	100 - 100	Semi-symmetric / semi-linear	Need forming and current compliance	*147*
TiN/TaO_x/HfO_2/TiN/Pt/Si	ALD / Filamentary	Identical +1.25V / -0.95V dt = 0.3 ms	1.2 - 8.5 × 10^{-5}	200 - 200	Asymmetric / non-linear	Need forming and current compliance	*148*
Al/TiO_x/HfO_2/ Pt/Ti/SiO_2/ Kapton polyimide	ALD / Filamentary	Non-identical +0.65-0.9V / -0.75-1.0V dt = 1 µs	1.4 - 2.2 × 10^{-5}	50 - 50	Symmetric / linear	Need forming and current compliance	*149*
W/TiO_2/HfO_2/ TaN	ALD / Filamentary	Identical +1.0V / -1.0V dt = 10 µs	0.5 - 5.5 × 10^{-5}	50 - 50	Symmetric / linear	Need forming and current compliance	*150*
Au/Ti/MoO_y/ HfO_2/WO_x/SiO_2/W/SiO_2/Si	Sputtering/ NA	Identical +4.0V / -2.5V dt = 10 ms	4.3 - 4.7 × 10^{-7}	500 - 500	Asymmetric / non-linear	NA	*151*
ITO/HfO_2/TiO_2/ITO/Glass	ALD / Filamentary	Identical +0.95V / -1.2V dt = 10 µs	1 - 1.12 × 10^{-3}	100 - 100	Symmetric / linear	Need forming and current compliance	*152*
Ta/Ti/Al_2O_3/ HfO_2/Pt/ SiO_2/Si	NA / Filamentary	NA	4.1 - 50 × 10^{-6}	2048	NA	NA	*72*
TiN/HfO_2 /Ti/TiN	ALD / Filamentary	Identical +0.45V / -0.5V dt = 1 ms	2 - 4 × 10^{-3}	100 - 100	Asymmetric / non-linear	Need forming and current compliance	*153*
ITO/Ag/WO_3/ HfO_2/n-Si	PLD / Filamentary	Identical +2V / -2V dt = 40 ms	1 - 35 × 10^{-6}	200 - 200	Asymmetric / non-linear	Need forming and current compliance	*154*
Pt/TiN/HfO_2/ WS_2/Pt/ SiO_2/Si	ALD / Filamentary	Identical +1.4V / -1.4V dt = 1 µs	4.3 - 4.7 × 10^{-3}	50 - 50	Semi-symmetric / semi-linear	Need forming and current compliance	*155*

/Mo/$HfZrO_2$/ HfO_2/n-Si	ALD / Interfacial	Identical +5.9V / -1.3V dt = 0.5 ms	3 - 5.5 × 10^{-3}	50 - 50	Asymmetric / non-linear	NA	*156*
Ti/HfO_2/HfO_x/ Pt/SiO_2/Si	ALD / Filamentary	Identical +1V / -1.1V dt = 1 μs	0.6 - 1.2 × 10^{-4}	60 - 60	Asymmetric / non-linear	Need forming and current compliance	*157*
W/IGZO/ $HfLaO_2$/ TaN/Si	ALD / NA	Non-identical +2-5V / -2-5V dt = 1 μs	0.5 - 4.5 × 10^{-5}	100 - 100	Symmetric / linear	NA	*158*
Pt/HfO_2/ $HfTaO_x$/ TiN/Si	Sputtering / Filamentary	Identical -0.85V / +1.1V dt = 10 μs	1.5 - 3.8 × 10^{-3}	100 - 100	Asymmetric / non-linear	Need forming and current compliance	*159*
Pt/$HfLaO_2$/ LSMO/STO/ Si	PLD / NA	Identical +5V /-5V dt = 0.5 μs	1.4 - 2.8 × 10^{-6}	100 - 60	Asymmetric / non-linear	NA	*160*
Ag/HfO_2 /NiO/Pt/ SiO_2/Si	Sputtering / Filamentary	Identical +0.4V / -0.45V dt = 1 μs	1 - 3 × 10^{-2}	50 - 50	Asymmetric / non-linear	Need forming and current compliance	*161*
Au/Sm:HfO_2 /FTO/Glass	Sol-Gel process / Filamentary	Identical +1.5V / -1.5V dt = 1 s	0.4 - 0.7 × 10^{-3}	50 - 50	Semi-symmetric / semi-linear	NA	*162*
Au/Ti/HfO_2/ Pt/Ti/SiO_2/Si	ALD / Filamentary	Identical +0.72V / -0.87V dt = 0.5 ms	0.1 - 0.7 × 10^{-3}	64 - 64	Asymmetric / non-linear	Need forming and current compliance	*163*
Mo/$HfAlO_2$/ TiN/SiO_2/Si	ALD / Filamentary	Identical +1.3V / -1.6V dt = 80 μs	0.6 - 1.9 × 10^{-3}	50 - 50	Asymmetric / non-linear	Need forming and current compliance	*164*
Pt/$HfZrO_2$/ Pt/Ti/SiO_2/Si	Sputtering / Filamentary	Identical +0.45V / -0.6V dt = 1 μs	0.1 - 0.3 × 10^{-3}	50 - 50	Asymmetric / non-linear	Need forming and current compliance	*165*
TiN/$HfAlO_2$ /n-Si	ALD / Interfacial	Identical +6.5V / -3.0V dt = 1 ms	2 - 9 × 10^{-6}	50 - 50	Asymmetric / non-linear	NA	*166*
Ti/TiO_2/HfO_2/ p-Si	ALD/ Sputtering / Interfacial	Identical +10.0V / -6.0V dt = 0.5 ms	3 - 18 × 10^{-9}	50 - 50	Asymmetric / non-linear	NA	*167*

* NA stands for not available data.

Table S2. Memristive characteristics reported for various devices with *p-n* heterostructures.

Device structure	*p*-type material	*n*-type material	LRS (Ω) at -0.5 V	HRS (Ω) at -0.5 V	Switching mechanism	Ref.
Au/Co_3O_4/ITO	Co_3O_4	ITO	~0.8×10^2	~1.3×10^4	Filamentary & interfacial	*168*
GaIn/HfO_2/NiO/ITO	NiO	HfO_2	~4.0×10^2	~5.0×10^4	Filamentary & interfacial	*169*
Au/$YMnO_3$/Nb:$SrTiO_3$	$YMnO_3$	Nb:$SrTiO_3$	~5.0×10^2	~5.0×10^6	Filamentary & interfacial	*116*
Au/ZnO/Ta_2O_5/Au	pure ZnO	doped ZnO	~5.0×10^6	~5.0×10^7	Filamentary & interfacial	*170*
Ti/ZnO/Si	Si	ZnO	~5.0×10^3	~5.0×10^5	Filamentary & interfacial	*171*
Ag/MoO_3/Cu_3SnS_4/Mo	Cu_3SnS_4	MoO_3	~5.0×10^2	~5.0×10^4	Filamentary & interfacial	*172*
$SrRuO_3$/$BiFeO_3$/Nb:$SrTiO_3$	$BiFeO_3$	Nb:$SrTiO_3$	~1.7×10^2	~5.0×10^2	Filamentary & interfacial	*173*
Al/NiO/TaO_X/ITO	NiO	TaO_X	~5.0×10^2	~5.0×10^3	Filamentary & interfacial	*118*
Al-doped ZnO_2/Zn_2SnO_4/Cu_2O/ F-doped SnO_2	Cu_2O	Zn_2SnO_4	~5.0×10^4	~5.0×10^6	Filamentary & interfacial	*174*
GaIn/NiO/ CeO_2/ITO	NiO	CeO_2	~1.0×10^1	~1.0×10^3	Filamentary & interfacial	*175*
GaIn/TiO_2(matrix)- CuO(particles)/ITO	CuO	TiO_2	~5.0×10^2	~5.0×10^5	Filamentary & interfacial	*176*
Al/ phenyl-C61-butyric acid methyl ester (PCBM)/poly(N-vinylcarba-zole) (PVK)/ITO	PVK	PCBM	~0.5×10^1	~5.0×10^6	Filamentary & interfacial	*177*
In/β-Ga_2O_3/NiO/In	NiO	β-Ga_2O_3	~8.3×10^4	~2.5×10^5	Filamentary & interfacial	*178*
In/β-Ga_2O_3/ Li-doped NiO/In	Li-doped NiO	β-Ga_2O_3	~1.0×10^2	~5.0×10^2	Filamentary & interfacial	*178*
Ag/NiO/ZnO/ITO	NiO	ZnO	~3.0×10^5	~6.0×10^5	Interfacial	*179*
Ni/ZnO(matrix)- Al(particles)/SI	Si	ZnO	~1.0×10^3	~1.0×10^5	Filamentary & interfacial	*180*
Ag/P_xO_y/$MoSe_2$/Mo	$MoSe_2$	P_xO_y	~5.0×10^2	~5.0×10^3	Filamentary & interfacial	*181*
$GdMnO_3$/ZnO/Nb:$SrTiO_3$	ZnO	$GdMnO_3$	~5.0×10^3	~5.0×10^5	Filamentary & interfacial	*182*
$GdMnO_3$/ Al-doped ZnO/Nb:$SrTiO_3$	Al-doped ZnO	$GdMnO_3$	~1.0×10^6	~2.0×10^6	Filamentary & interfacial	*182*
ITO/ZnO/ polymethylmethacrylate PMMA(intrinsic)/ CuSCN/ITO	ZnO	CuSCN	~5.0×10^5	~2.5×10^7	Filamentary & interfacial	*183*

Au/SnSe/ITO	SnSe	ITO	~1.0×10^{4}	~1.0×10^{5}	Filamentary & interfacial	*184*
ITO/*p*-IGZO/n-NiO/Ni/Au	NiO	IGZO	~5.0×10^{2}	~5.0×10^{4}	Filamentary & interfacial	*185*
Pt/NiO/TiO_2/Pt	NiO	TiO_2	NA	NA	Filamentary & interfacial	*186*
Si/NiO/Ni	NiO	Si	NA	NA	Filamentary & interfacial	*187*
Pt/NiO/ $Mg_{0.6}Zn_{0.4}O$/Pt	NiO	$Mg_{0.6}Zn_{0.4}O$	NA	NA	Filamentary & interfacial	*188*
Mo/Hf(Sr,Ti)O_2/TiN/SiO_2/Si	Hf(Sr,Ti)O_2	O-rich TiO_xN_y	~7.0×10^{7}	~8.0×10^{9}	Interfacial	*This work*
Ag/$BiFeO_3$/$Cu_2ZnSnSe_4$/ F-doped TiO_2	$BiFeO_3$	$Cu_2ZnSnSe_4$	~2.0×10^{2}	~2.5×10^{2}	Interfacial	*120*
Ni/ZnO/SI	Si	ZnO	~2.0×10^{9}	~4.0×10^{9}	Interfacial	*189*
Au/$BiFeO_3$/Pt	Bi-deficient $BiFeO_3$	Bi-rich $BiFeO_3$	~8.0×10^{2}	~4.0×10^{3}	Interfacial	*119*
Au-coated SiN/Bi_2S_3/ Cu_2FeSnS_4/ITO	Cu_2FeSnS_4	Bi_2S_3	~2.5×10^{2}	~5.0×10^{2}	Interfacial	*190*
Pt/TiO_x/TiN	O-rich TiO_x	O-poor TiO_x	~5.0×10^{2}	~5.0×10^{4}	Interfacial	*98*
Au/$BiFeO_3$/Nb:$SrTiO_3$	Bi-deficient $BiFeO_3$	Nb:$SrTiO_3$	~4.3×10^{6}	~5.0×10^{8}	Interfacial	*191*
Au/$BiFeO_3$/ $La_{2/3}Sr_{1/3}MnO_3$/ $SrTiO_3$	Bi-deficient $BiFeO_3$	$La_{2/3}Sr_{1/3}MnO_3$	~5.0×10^{7}	~5.0×10^{9}	Interfacial	*191*
Au/$BiFeO_3$/$SrRuO_3$/ $SrTiO_3$	$SrRuO_3$	O-deficient $BiFeO_3$	~9.0×10^{7}	~1.0×10^{8}	Interfacial	*191*
Ag/NiO/ $Cs_2AgBiBr_6$(intrinsic)/ SnO_2/ITO	NiO	SnO_2	~5.0×10^{2}	~8.5×10^{2}	Interfacial	*192*
Ag/graphene oxide/ZnO/Ag	graphene oxide	O- deficient ZnO	~1.0×10^{9}	~5.0×10^{9}	Interfacial	*193*
Au/Bi_2Te_3/Si	Si	Bi_2Te_3	~2.0×10^{5}	~2.0×10^{6}	Interfacial	*194*
Pt/WO_X/NiO/ITO	NiO	WO_X	~5.0×10^{5}	~5.0×10^{6}	Interfacial	*195*
Al/ZnO/NiO/ITO	NiO	ZnO	~5.0×10^{5}	~5.0×10^{7}	Interfacial	*196*
Au/ZnO/NiO/ITO	NiO	ZnO	~5.0×10^{6}	~1.0×10^{7}	Interfacial	*196*
Pt/ZnO/NiO/TiN	NiO	ZnO	~3.0×10^{4}	~5.0×10^{5}	Interfacial	*197*
Pt/$La_{0.7}Ca_{0.3}MnO_3$ (LCMO)/TiO_2/Ti	LCMO	TiO_2	~2.0×10^{2}	~1.0×10^{3}	Interfacial	*198*
Au/ZnO/NiO/Pt	NiO	ZnO	NA	NA	Interfacial	*71*
Pt/ZnO/$CuAlO_2$/Pt	$CuAlO_2$	ZnO	NA	NA	Interfacial	*71*
Ni/ZnO/Si	Si	ZnO	NA	NA	Interfacial	*71*
ITO/ ZnO(intrinsic)/ NiO/Ni	NiO	ITO	NA	NA	Interfacial	*199*

* NA stands for not available data.

REFERENCES

1. S. Kumar, X. Wang, J. P. Strachan, Y. Yang, W. D. Lu, Dynamical memristors for higher-complexity neuromorphic computing. *Nat. Rev. Mater.* **7**, 575–591 (2022).

2. F. C. Morabito, R. Kozma, C. Alippi, Y. Choe, “Advances in AI, neural networks, and brain computing: An introduction” in *Artificial Intelligence in the Age of Neural Networks and Brain Computing* (Elsevier, 2024).

3. Z. Liu, J. Mei, J. Tang, M. Xu, B. Gao, K. Wang, S. Ding, Q. Liu, Q. Qin, W. Chen, Y. Xi, Y. Li, P. Yao, H. Zhao, N. Wong, H. Qian, B. Hong, T. P. Jung, D. Ming, H. Wu, A memristor-based adaptive neuromorphic decoder for brain-computer interfaces. *Nat. Electron.* **8**, 362–372 (2025).

4. D. Kudithipudi, C. Schuman, C. M. Vineyard, T. Pandit, C. Merkel, R. Kubendran, J. B. Aimone, G. Orchard, C. Mayr, R. Benosman, J. Hays, C. Young, C. Bartolozzi, A. Majumdar, S. G. Cardwell, M. Payvand, S. Buckley, S. Kulkarni, H. A. Gonzalez, G. Cauwenberghs, C. S. Thakur, A. Subramoney, S. Furber, Neuromorphic computing at scale. *Nature* **637**, 801–812 (2025).

5. T. Shi, L. Gao, R. Zhou, Y. Tian, P. Chen, Y. Ding, S. Tang, H. Ma, J. Lu, H. Zhang, Z. Wang, B. Lyu, X. Zhang, X. Yan, Q. Liu, Fully memristive spiking neural network for energy-efficient graph learning. *Sci. Adv.* **11**, eadv2312 (2025).

6. K. Jeon, J. J. Ryu, S. Im, H. K. Seo, T. Eom, H. Ju, M. K. Yang, D. S. Jeong, G. H. Kim, Purely self-rectifying memristor-based passive crossbar array for artificial neural network accelerators. *Nat. Comm.* **15**, 129 (2024).

7. K. Roy, A. Jaiswal, P. Panda, Towards spike-based machine intelligence with neuromorphic computing. *Nature* **575**, 607–617 (2019).

8. M. A. Zidan, J. P. Strachan, W. D. Lu, The future of electronics based on memristive systems. *Nat. Electron.* **1**, 22–29 (2018).

9. Z. Wang, R. Yu, Z. Jia, Z. He, T. Yang, B. Gao, Y. Li, Z. Hu, Z. Hao, Y. Liu, J. Lu, P. Yao, J. Tang, Q. Liu, H. Qian, H. Wu, A dual-domain compute-in-memory system for general neural network inference. *Nat. Electron.* **8**, 276–287 (2025).

10. D. Ielmini, H. S. P. Wong, In-memory computing with resistive switching devices. *Nat. Electron.* **1**, 333–343 (2018).

11. W. Zhang, B. Gao, J. Tang, P. Yao, S. Yu, M. F. Chang, H. J. Yoo, H. Qian, H. Wu, Neuro-inspired computing chips. *Nat. Electron.* **3**, 371–382 (2020).

12. V. Avelar, P. Lin, W. Torell, M. A. Torres Arango, White Paper on The AI disruption: Challenges and guidance for data center design. (2023).

13. A. Mehonic, A. J. Kenyon, Brain-inspired computing needs a master plan. *Nature* **604**, 255–260 (2022).

14. Z. Wang, H. Wu, G. W. Burr, C. S. Hwang, K. L. Wang, Q. Xia, J. J. Yang, Resistive switching materials for information processing. *Nat. Rev. Mater.* **5**, 173–195 (2020).

15. F. Yang, Y. Jang, C. Wang, J. Liang, Y. Li, Metal oxide-based neuromorphic artificial visual perception devices and systems for information perception, memory and processing. *Adv. Funct. Mater.* **36**, e15213 (2026).

16. N. Jones, How to stop data centres from gobbling up the world’s electricity. *Nature* **561**, 163–166 (2018).

17. R. Verdecchia, J. Sallou, L. Cruz, A systematic review of green AI. *WIREs Data. Min. Knowl. Discov.* **13**, e1507 (2023).

18. D. S. Woo, S. M. Jin, J. K. Kim, G. H. Park, W. G. Lee, M. J. Han, J. H. Kim, T. H. Shim, J. Park, J. G. Park, Unveiling the resistive switching mechanism and low current dynamics of Ru-based hybrid synaptic memristors. *Adv. Funct. Mater.* **35**, 2416309 (2025).

19. C. S. Hwang, Prospective of semiconductor memory devices: From memory system to materials. *Adv. Electron. Mater.* **1**, 1400056 (2015).

20. V. K. Sangwan, M. C. Hersam, Neuromorphic nanoelectronic materials. *Nat. Nanotechnol.* **15**, 517–528 (2020).

21. A. Sebastian, M. Le Gallo, R. Khaddam-Aljameh, E. Eleftheriou, Memory devices and applications for in-memory computing. *Nat. Nanotechnol.* **15**, 529–544 (2020).

22. D. V. Christensen, R. Dittmann, B. Linares-Barranco, A. Sebastian, M. Le Gallo, A. Redaelli, S. Slesazeck, T. Mikolajick, S. Spiga, S. Menzel, 2022 roadmap on neuromorphic computing and engineering. *Neuromorphic Comput. Eng.* **2**, 022501 (2022).

23. G. W. Burr, R. M. Shelby, A. Sebastian, S. Kim, S. Kim, S. Sidler, K. Virwani, M. Ishii, P. Narayanan, A. Fumarola, L. L. Sanches, I. Boybat, M. Le Gallo, K. Moon, J. Woo, H. Hwang, Y. Leblebici, Neuromorphic computing using non-volatile memory. *Adv. Phys. X* **2**, 89–124 (2017).

24. M. Bear, B. Connors, M. A. Paradiso, Neuroscience: Exploring the brain, enhanced edition: Exploring the brain (Jones & Bartlett Learning, 2020).

25. S. Chen, T. Zhang, S. Tappertzhofen, Y. Yang, I. Valov, Electrochemical-memristor-based artificial neurons and synapses-fundamentals, applications, and challenges. *Adv. Mater.* **35**, 2301924 (2023).

26. T. J. Park, S. Deng, S. Manna, A. N. M. N. Islam, H. Yu, Y. Yuan, D. D. Fong, A. A. Chubykin, A. Sengupta, S. K. R. S. Sankaranarayanan, S. Ramanathan, Complex oxides for brain-inspired computing: A review. *Adv. Mater.* **35**, 2203352 (2023).

27. H. Ning, Z. Yu, Q. Zhang, H. Wen, B. Gao, Y. Mao, Y. Li, Y. Zhou, Y. Zhou, J. Chen, L. Liu, W. Wang, T. Li, Y. Li, W. Meng, W. Li, Y. Li, H. Qiu, Y. Shi, Y. Chai, H. Wu, X. Wang, An in-memory computing architecture based on a duplex two-dimensional material structure for in situ machine learning. *Nat. Nanotechnol.* **18**, 493–500 (2023).

28. C. López, Artificial intelligence and advanced materials. *Adv. Mater.* **35**, 2208683 (2023).

29. M. Vasilopoulou, Neuromorphic computing based on halide perovskites. *Nat. Electron.* **6**, 949–962 (2023).

30. M. Lanza, A. Sebastian, W. D. Lu, M. Le Gallo, M. F. Chang, D. Akinwande, F. M. Puglisi, H. N. Alshareef, M. Liu, J. B. Roldan, Memristive technologies for data storage, computation, encryption, and radio-frequency communication. *Science* **376**, eabj9979 (2022).

31. H. S. P. Wong, S. Salahuddin, Memory leads the way to better computing. *Nat. Nanotechnol.* **10**, 191–194 (2015).

32. X. Liu, C. Sun, X. Ye, X. Zhu, C. Hu, H. Tan, S. He, M. Shao, R. W. Li, Neuromorphic nanoionics for human-machine interaction: From materials to applications. *Adv. Mater.* **36**, 2311472 (2024).

33. Z. Yuan, B. Bakhit, Y. X. Liu, Z. Sun, G. I. Lampronti, X. Li, S. M. Fairclough, B. K. Tsai, A. Choudhury, C. Ducati, H. Wang, M. Hellenbrand, J. L. MacManus-Driscoll, Engineered high endurance in WO3-based resistive switching devices via a guided filament approach. *Sci. Adv.* **11**, eadt9789 (2025).

34. X. Duan, Z. Cao, K. Gao, W. Yan, S. Sun, G. Zhou, Z. Wu, F. Ren, B. Sun, Memristor-based neuromorphic chips. *Adv. Mater.* **36**, 2310704 (2024).

35. W. Banerjee, A. Kashir, S. Kamba, Hafnium Oxide (HfO_2) – A multifunctional oxide: A review on the prospect and challenges of hafnium oxide in resistive switching and ferroelectric memories. *Small* **18**, 2107575 (2022).

36. R. Dittmann, S. Menzel, R. Waser, Nanoionic memristive phenomena in metal oxides: The valence change mechanism. *Adv. Phys.* **70**, 155–349 (2021).

37. Y. Xiao, B. Jiang, Z. Zhang, S. Ke, Y. Jin, X. Wen, C. Ye, A review of memristor: Material and structure design, device performance, applications and prospects. *Sci. Technol. Adv. Mater.* **24**, 2162323 (2023).

38. J. B. Roldán, E. Miranda, D. Maldonado, A. N. Mikhaylov, N. V. Agudov, A. A. Dubkov, M. N. Koryazhkina, M. B. González, M. A. Villena, S. Poblador, M. Saludes-Tapia, R. Picos, F. Jiménez-Molinos, S. G. Stavrinides, E. Salvador, F. J. Alonso, F. Campabadal, B. Spagnolo, M. Lanza, L. O. Chua, Variability in resistive memories. *Adv. Intell. Syst.* **5**, 2200338 (2023).

39. S. Yoo, S. Chae, T. Chiang, M. Webb, T. Ma, H. Paik, Y. Park, L. Williams, K. Nomoto, H. G. Xing, S. Trolier-McKinstry, E. Kioupakis, J. T. Heron, W. D. Lu, Efficient data processing using tunable entropy-stabilized oxide memristors. *Nat. Electron.* **7**, 466–474 (2024).

40. X. Yan, J. H. Qian, V. K. Sangwan, M. C. Hersam, Progress and challenges for memtransistors in neuromorphic circuits and systems. *Adv. Mater.* **34**, 2108025 (2022).

41. H. Na, S. Kim, Enhanced reliability and controllability in filamentary oxide-based 3D vertical structured resistive memory with pulse scheme algorithm for versatile neuromorphic applications. *Adv. Funct. Mater.* **35**, 2500956 (2025).

42. S. Chen, Z. Yang, H. Hartmann, A. Besmehn, Y. Yang, I. Valov, Electrochemical ohmic memristors for continual learning. *Nat. Comm.* **16**, 2348 (2025).

43. M. Rao, H. Tang, J. Wu, W. Song, M. Zhang, W. Yin, Y. Zhuo, F. Kiani, B. Chen, X. Jiang, H. Liu, H.-Y. Chen, R. Midya, F. Ye, H. Jiang, Z. Wang, M. Wu, M. Hu, H. Wang, Q. Xia, N. Ge, J. Li, J. J. Yang, Thousands of conductance levels in memristors integrated on CMOS. *Nature* **615**, 823–829 (2023).

44. S. V. Vegesna, V. R. Rayapati, H. Schmidt, Transport properties of interface-type analog memristors. *Phys. Rev. Appl.* **22**, 034028 (2024).

45. F. Zahari, R. Marquardt, M. Kalläne, O. Gronenberg, C. Schlueter, Y. Matveyev, G. Haberfehlner, F. Diekmann, A. Nierhauve, J. Buck, A. Hanff, G. Kolhatkar, G. Kothleitner, L. Kienle, M. Ziegler, J. Carstensen, K. Rossnagel, H. Kohlstedt, Trap-assisted memristive switching in HfO_2-based devices studied by in situ soft and hard x-ray photoelectron spectroscopy. *Adv. Electron. Mater.* **9**, 2201226 (2023).

46. D. Spassov, A. Paskaleva, E. Guziewicz, W. Wozniak, T. Stanchev, T. Ivanov, J. Wojewoda-Budka, M. Janusz-Skuza, Charge storage and reliability characteristics of nonvolatile memory capacitors with HfO_2/Al_2O_3-based charge trapping layers. *Dent. Mater.* **15**, 6285 (2022).

47. S. Park, B. Spetzler, T. Ivanov, M. Ziegler, Multilayer redox-based $HfO_x/Al_2O_3/TiO_2$ memristive structures for neuromorphic computing. *Sci. Rep.* **12**, 18266 (2022).

48. A. Kumar, S. Mondal, K. S. R. Koteswara Rao, Probing the oxygen vacancy associated native defects in high-κ HfO_2 using deep level transient spectroscopy. *J. Appl. Phys.* **135**, 045305 (2024).

49. M. Ismail, M. Rasheed, C. Mahata, M. Kang, S. Kim, Mimicking biological synapses with a-$HfSiO_x$-based memristor: Implications for artificial intelligence and memory applications. *Nano Converg.* **10**, 33 (2023).

50. D. Ielmini, F. Nardi, C. Cagli, A. L. Lacaita, Size-dependent retention time in NiO-based resistive-switching memories. *IEEE Electron Device Lett.* **31**, 353–355 (2010).

51. Y. D. Lin, P. S. Chen, H. Y. Lee, Y. S. Chen, S. Z. Rahaman, K. H. Tsai, C. H. Hsu, W. S. Chen, P. H. Wang, Y. C. King, C. J. Lin, Retention model of TaO/HfOx and TaO/AlOx RRAM with self-rectifying switch characteristics. *Nanoscale Res. Lett.* **12**, 407 (2017).

52. E. Perez, M. K. Mahadevaiah, C. Zambelli, P. Olivo, C. Wenger, Data retention investigation in Al:HfO_2-based resistive random access memory arrays by using high-temperature accelerated tests. *J. Vac. Sci. Technol. B* **37**, 012202 (2019).

53. S. R. Bradley, A. L. Shluger, G. Bersuker, Electron-injection-assisted generation of oxygen vacancies in monoclinic HfO_2. *Phys. Rev. Appl.* **4**, 064008 (2015).

54. R. Izmailov, J. Strand, N. Ronchi, A. Shluger, V. Afanasev, Electron emission from deep traps in HfO_2 under thermal and optical excitation. *Phys. Rev. B* **109**, 134109 (2024).

55. M. Huang, L. Xu, J. A. del Alamo, J. Li, B. Yildiz, Nonlinear ion dynamics enable spike timing dependent plasticity of electrochemical ionic synapses. *Adv. Mater.* **37**, 2418484 (2025).

56. K. Yang, J. Joshua Yang, R. Huang, Y. Yang, Nonlinearity in memristors for neuromorphic dynamic systems. *Small Sci.* **2**, 2100049 (2022).

57. W. Huang, X. Xia, C. Zhu, P. Steichen, W. Quan, W. Mao, J. Yang, L. Chu, Memristive artificial synapses for neuromorphic computing. *Nano Micro Lett.* **13**, 85 (2021).

58. J. Li, H. Abbas, D. S. Ang, A. Ali, X. Ju, Emerging memristive artificial neuron and synapse devices for the neuromorphic electronics era. *Nanoscale Horiz.* **8**, 1456–1484 (2023).

59. Y. H. Liu, L. Q. Zhu, P. Feng, Y. Shi, Q. Wan, Freestanding artificial synapses based on laterally proton-coupled transistors on chitosan membranes. *Adv. Mater.* **27**, 5599–5604 (2015).

60. M. Shahsavari, P. Devienne, P. Boulet, "Spiking neural computing in memristive neuromorphic platforms," in *Handbook of Memristor Networks*, L. Chua, G. C. Sirakoulis, A. Adamatzky, Eds. (Springer International Publishing, 2019).

61. F. Hofer, P. Warbichler, A. Scott, R. Brydson, I. Galesic, B. Kolbesen, Electron energy loss near edge structure on the nitrogen K-edge in vanadium nitrides. *J. Microsc.* **204**, 166–171 (2001).

62. Y. Kihn, C. Mirguet, L. Calmels, EELS studies of Ti-bearing materials and ab initio calculations. *J. Electron Spectrosc. Relat. Phenom.* **143**, 117–127 (2005).

63. Y. Zhang, G. Q. Mao, X. Zhao, Y. Li, M. Zhang, Z. Wu, W. Wu, H. Sun, Y. Guo, L. Wang, X. Zhang, Q. Liu, H. Lv, K. H. Xue, G. Xu, X. Miao, S. Long, M. Liu, Evolution of the conductive filament system in HfO_2-based memristors observed by direct atomic-scale imaging. *Nat. Comm.* **12**, 7232 (2021).

64. S. U. Sharath, S. Vogel, L. Molina-Luna, E. Hildebrandt, C. Wenger, J. Kurian, M. Duerrschnabel, T. Niermann, G. Niu, P. Calka, M. Lehmann, H.-J. Kleebe, T. Schroeder, L. Alff, Control of switching modes and conductance quantization in oxygen engineered HfO based memristive devices. *Adv. Funct. Mater.* **27**, 1700432 (2017).

65. V. C. Anitha, A. N. Banerjee, S. W. Joo, Recent developments in TiO_2 as n- and p-type transparent semiconductors: Synthesis, modification, properties, and energy-related applications. *J. Mater. Sci.* **50**, 7495–7536 (2015).

66. T. Zhu, L. Ma, S. Deng, S. Liu, Progress in computational understanding of ferroelectric mechanisms in HfO_2. *npj Comput. Mater.* **10**, 188 (2024).

67. M. H. Park, T. Schenk, M. Hoffmann, S. Knebel, J. Gärtner, T. Mikolajick, U. Schroeder, Effect of acceptor doping on phase transitions of HfO_2 thin films for energy-related applications. *Nano Energy* **36**, 381–389 (2017).

68. H. Li, Y. Guo, J. Robertson, Hydrostatic piezoelectric properties of [011] poled $Pb(Mg_{1/3}Nb_{2/3})O_3$-$PbTiO_3$ single crystals and 2-2 lamellar composites. *Appl. Phys. Lett.* **104**, 032909 (2014).

69. J. Robertson, S. J. Clark, Limits to doping in oxides. *Phys. Rev. B* **83**, 075205 (2011).

70. N. Kaiser, T. Vogel, A. Zintler, S. Petzold, A. Arzumanov, E. Piros, R. Eilhardt, L. Molina-Luna, L. Alff, Defect-stabilized substoichiometric polymorphs of hafnium oxide with semiconducting properties. *ACS Appl. Mater. Interfaces* **14**, 1290–1303 (2022).

71. X. Guo, X. Li, R. Wang, W. Zhu, L. Wang, L. Zhang, Building a depletion-region width modulation model and realizing memory characteristics in PN heterostructure devices. *Nanoscale* **16**, 15722–15729 (2024).

72. B. Bakhit, M. Hellenbrand, B. K. Tsai, A. Choudhury, P. Polcik, S. Kolozsvari, H. Wang, A. J. Flewitt, J. L. MacManus-Driscoll, Enhanced non-volatile resistive switching performance through ion-assisted magnetron sputtering of TiN bottom electrodes. *Comm. Mater.* **6**, 77 (2025).

73. G. Milano, M. Luebben, M. Laurenti, L. Boarino, C. Ricciardi, I. Valov, Structure-dependent influence of moisture on resistive switching behavior of ZnO thin films. *Adv. Mater. Interfaces* **8**, 2100915 (2021).

74. B. Bakhit, D. Primetzhofer, E. Pitthan, M. A. Sortica, E. Ntemou, J. Rosen, L. Hultman, I. Petrov, G. Greczynski, Systematic compositional analysis of sputter-deposited boron-containing thin films. *J. Vac. Sci. Technol. A* **39**, 063408 (2021).

75. P. Makuła, M. Pacia, W. Macyk, How to correctly determine the band gap energy of modified semiconductor photocatalysts based on UV-Vis spectra. *J. Phys. Chem. Lett.* **9**, 6814–6817 (2018).

76. G. Kresse, J. Furthmüller, Efficient iterative schemes for ab initio total-energy calculations using a plane-wave basis set. *Phys. Rev. B* **54**, 11169–11186 (1996).

77. G. Kresse, D. Joubert, From ultrasoft pseudopotentials to the projector augmented-wave method. *Phys. Rev. B* **59**, 1758–1775 (1999).

78. A. V. Krukau, O. A. Vydrov, A. F. Izmaylov, G. E. Scuseria, Influence of the exchange screening parameter on the performance of screened hybrid functionals. *Chem. Phys.* **125**, 224106 (2006).

79. B. Z. Rico Haeuselmann, A. Togo, Espen, D. Gresch, K. Shinohara, J. Chico, mcallsen, S. Huber, M. Uhrin, S. Kavanagh, M. Wolloch, D. Marchand, J. Yu, N. Keilbart, V. Nikolaev, aiida-vasp/aiida-vasp: V4.1.0 (v4.1.0) (2024). DOI: 10.5281/zenodo.11044628

80. A. Goyal, P. Gorai, H. Peng, S. Lany, V. Stevanović, A computational framework for automation of point defect calculations. *Comput. Mater. Sci.* **130**, 1–9 (2017).

81. Y. Kumagai, N. Tsunoda, A. Takahashi, F. Oba, Insights into oxygen vacancies from high-throughput first-principles calculations. *Phys. Rev. Materials* **5**, 123803 (2021).

82. S. R. Kavanagh, A. G. Squires, A. Nicolson, I. Mosquera-Lois, A. M. Ganose, B. Zhu, K. Brlec, A. Walsh, D. O. Scanlon, doped: Python toolkit for robust and repeatable charged defect supercell calculations. *J. Open Source Software* **9**, 6433 (2024).

83. A. G. Squires, D. O. Scanlon, B. J. Morgan, py-sc-fermi: Self-consistent Fermi energies and defect concentrations from electronic structure calculations. *J. Open Source Softw.* **8**, 4962 (2023).

84. B. Tang, X. Li, J. Liao, Q. Chen, Ultralow power consumption and large dynamic range synaptic transistor based on α-In_2Se_3 nanosheets. *ACS Appl. Electron. Mater.* **4**, 598–605 (2022).

85. M. Hellenbrand, B. Bakhit, H. Dou, M. Xiao, M. O. Hill, Z. Sun, A. Mehonic, A. Chen, Q. Jia, H. Wang, J. L. MacManus-Driscoll, Thin film design of amorphous hafnium oxide nanocomposites enabling strong interfacial resistive switching uniformity. *Sci. Adv.* **9**, eadg1946 (2023).

86. W. Martienssen, H. Warlimont, in *Springer Handbook of Condensed Matter and Materials Data*. Springer Handbooks (Springer, 2005), 10.1007/3-540-30437-1.

87. S. S. Zumdahl, *Chemical Principles* (Houghton Mifflin Company, 2009).

88. K. T. Jacob, V. S. Saji, J. Gopalakrishnan, Y. Waseda, Thermodynamic evidence for phase transition in $MoO_{2-\delta}$. *J. Chem. Thermodyn.* **39**, 1539–1545 (2007).

89. K. A. Hussain, G. M. Ali, A. Boubaker, A. Kalboussi, Richardson constant and characteristics of pentacene organic planar Schottky diode. *Thin Solid Films* **771**, 139800 (2023).

90. R. K. Sharma, D. Rawal, in *The Physics of Semiconductor Devices: Proceedings of IWPSD 2017*. Springer Proceedings in Physics (Springer Cham, 2019), vol. 215; https://doi.org/10.1007/978-3-319-97604-4.

91. R. Green, White Paper 3111 on Hall effect measurements in materials characterization. (2011).

92. M. J. Deen, F. Pascal, "Electrical characterization of semiconductor materials and devices," *Springer Handbook of Electronic and Photonic Materials*, *Springer Handbooks*. S. Kasap, P. Capper, Eds. (Springer, 2017); https://doi.org/10.1007/978-3-319-48933-9_20.

93. V. Mansfeldova, M. Zlamalova, H. Tarabkova, P. Janda, M. Vorokhta, L. Piliai, L. Kavan, Work function of TiO_2 (Anatase, Rutile, and Brookite) single crystals: Effects of the environment. *J. Phys. Chem. C* **125**, 1902–1912 (2021).

94. S. Kashiwaya, J. Morasch, V. Streibel, T. Toupance, W. Jaegermann, A. Klein, The work function of TiO_2. *Surfaces* **1**, 73–89 (2018).

95. K. D. Schierbaum, U. K. Kirner, J. F. Geiger, W. Göpel, Schottky-barrier and conductivity gas sensors based upon Pd/SnO_2 and Pt/TiO_2. *Sens. Actuators B Chem.* **4**, 87–94 (1991).

96. C. Kaewmeechai, Y. Laosiritaworn, A. P. Jaroenjittichai, Band alignment of Cs_2BX_6 double halide perovskites and TiO_2 using electron affinity rule. *Results Phys.* **42**, 106015 (2022).

97. R. Könenkamp, I. Rieck, Electrical properties of Schottky diodes on nano-porous TiO_2 films. *Mater. Sci. Eng. B* **69-70**, 519–521 (2000).

98. D. Zhang, R. Dhall, M. M. Schneider, C. Song, H. Dou, S. Kunwar, N. R. Yazzie, J. Ciston, N. G. Cucciniello, P. Roy, In-situ study of understanding the resistive switching mechanisms of nitride-based memristor devices. arXiv:2410.23185 [physics.app-ph] (2024).

99. X. Cheng, X. Yu, Z. Xing, L. Yang, Synthesis and characterization of N-doped TiO_2 and its enhanced visible-light photocatalytic activity. *Arab. J. Chem.* **9**, S1706–S1711 (2016).

100. Z. Zhang, Z. Luo, Z. Yang, S. Zhang, Y. Zhang, Y. Zhou, X. Wang, X. Fu, Band-gap tuning of N-doped TiO_2 photocatalysts for visible-light-driven selective oxidation of alcohols to aldehydes in water. *RSC Adv.* **3**, 7215–7218 (2013).

101. E. M. Samsudin, S. B. Abd Hamid, Effect of band gap engineering in anionic-doped TiO_2 photocatalyst. *Appl. Surf. Sci.* **391**, 326–336 (2017).

102. H. Borkar, A. Thakre, S. S. Kushvaha, R. P. Aloysius, A. Kumar, Light assisted irreversible resistive switching in ultra thin hafnium oxide. *RSC Adv.* **5**, 35046–35051 (2015).

103. L. Baumgarten, T. Szyjka, T. Mittmann, A. Gloskovskii, C. Schlueter, T. Mikolajick, U. Schroeder, M. Müller, Smart design of fermi level pinning in HfO_2-based ferroelectric memories. *Adv. Funct. Mater.* **34**, 2307120 (2024).

104. Y. S. Kim, H. Chung, S. Kwon, J. Kim, W. Jo, Grain boundary passivation via balancing feedback of hole barrier modulation in HfO_{2-x} for nanoscale flexible electronics. *Nano Converg.* **9**, 43 (2022).

105. L. Tang, C. Chen, A. Wei, K. Li, D. Zhang, K. Zhou, Regulating crystal structure and ferroelectricity in Sr doped HfO_2 thin films fabricated by metallo-organic decomposition. *Ceram. Int.* **45**, 3140–3147 (2019).

106. M. Badillo, S. Taleb, B. Carreno Jimenez, T. Mokabber, R. Castanedo Pérez, G. Torres-Delgado, B. Noheda, M. Acuautla, (001)-oriented Sr:HfO_2 ferroelectric films deposited by a flexible chemical solution method. *ACS Appl. Electron. Mater.* **6**, 1809–1820 (2024).

107. G. Lee, J. Bae, H. J. Yoon, J. Oh, K. Kim, W. Kim, H. Jeon, Impact of Ti doping on the crystallinity and electrical properties of HfO_2 thin films using atomic layer deposition. doi.org/10.2139/ssrn.4973677.

108. D. I. Shahin, M. J. Tadjer, V. D. Wheeler, A. D. Koehler, T. J. Anderson, C. R. Eddy, Jr., A. Christou, Electrical characterization of ALD HfO_2 high-k dielectrics on ($\bar{2}01$) β-Ga_2O_3. *Appl. Phys. Lett.* **112**, 042107 (2018).

109. D. R. Islamov, V. A. Gritsenko, T. V. Perevalov, V. A. Pustovarov, O. M. Orlov, A. G. Chernikova, A. M. Markeev, S. Slesazeck, U. Schroeder, T. Mikolajick, G. Y. Krasnikov, Identification of the nature of traps involved in the field cycling of Hf0.5Zr0.5O2-based ferroelectric thin films. *Acta Mater.* **166**, 47–55 (2019).

110. D. R. Islamov, T. V. Perevalov, Effect of oxygen vacancies on the ferroelectric $Hf_{0.5}Zr_{0.5}O_2$ stabilization: DFT simulation. *Microelectron. Eng.* **216**, 111041 (2019).

111. E. Hildebrandt, J. Kurian, M. M. Müller, T. Schroeder, H. J. Kleebe, L. Alff, Controlled oxygen vacancy induced p-type conductivity in HfO_{2-x} thin films. *Appl. Phys. Lett.* **99**, 112902 (2011).

112. H. Tang, K. Prasad, R. Sanjinès, P. E. Schmid, F. Lévy, Electrical and optical properties of TiO_2 anatase thin films. *J. Appl. Phys.* **75**, 2042–2047 (1994).

113. J. Liu, W. Yang, Synthesis and electron irradiation modification of anatase TiO_2 with different morphologies. *Ceram. Int.* **48**, 10428–10437 (2022).

114. B. Yan, D. Wan, X. Chi, C. Li, M. R. Motapothula, S. Hooda, P. Yang, Z. Huang, S. Zeng, A. G. Ramesh, S. J. Pennycook, A. Rusydi, J. Ariando, T. V. Martin, T. Venkatesan, Anatase TiO_2-A model system for large polaron transport. *ACS Appl. Mater. Interfaces* **10**, 38201–38208 (2018).

115. C. Funck, S. Menzel, Comprehensive model of electron conduction in oxide-based memristive devices. *ACS Appl. Electron. Mater.* **3**, 3674–3692 (2021).

116. A. Bogusz, D. Bürger, I. Skorupa, O. G. Schmidt, H. Schmidt, Bipolar resistive switching in $YMnO_3$/Nb:$SrTiO_3$ pn-heterojunctions. *Nanotechnology* **27**, 455201 (2016).

117. K. C. Kao, *Dielectric Phenomena in Solids* (Elsevier, 2004).

118. Y. B. Zhu, K. Zheng, X. Wu, L. K. Ang, Enhanced stability of filament-type resistive switching by interface engineering. *Sci. Rep.* **7**, 43664 (2017).

119. X. Tang, X. Zhu, J. Dai, J. Yang, L. Chen, Y. Sun, Evolution of the resistive switching in chemical solution deposited-derived $BiFeO_3$ thin films with dwell time and annealing temperature. *J. Appl. Phys.* **113**, 043706 (2013).

120. X. Li, B. Sun, W. Hou, J. Chen, P. Zheng, S. Mao, S. Zhu, Y. Xia, G. Fu, Environmental factors controlled resistive switching memory behavior based on $BiFeO_3$/$Cu_2ZnSnSe_4$ heterojunction. *Results Phys.* **13**, 102308 (2019).

121. S. Chandrasekaran, F. M. Simanjuntak, R. Saminathan, D. Panda, T. Y. Tseng, Improving linearity by introducing Al in HfO_2 as a memristor synapse device. *Nanotechnology* **30**, 445205 (2019).

122. H. Jiang, L. Han, P. Lin, Z. Wang, M. H. Jang, Q. Wu, M. Barnell, J. J. Yang, H. L. Xin, Q. Xia, Sub-10 nm Ta channel responsible for superior performance of a HfO_2 memristor. *Sci. Rep.* **6**, 28525 (2016).

123. S. Kim, Y. Abbas, Y. R. Jeon, A. S. Sokolov, B. Ku, C. Choi, Engineering synaptic characteristics of TaO_x/HfO_2 bi-layered resistive switching device. *Nanotechnology* **29**, 415204 (2018).

124. C. Li, D. Belkin, Y. Li, P. Yan, M. Hu, N. Ge, H. Jiang, E. Montgomery, P. Lin, Z. Wang, W. Song, J. P. Strachan, M. Barnell, Q. Wu, R. S. Williams, J. J. Yang, Q. Xia, Efficient and self-adaptive in-situ learning in multilayer memristor neural networks. *Nat. Comm.* **9**, 2385 (2018).

125. J. Frascaroli, S. Brivio, E. Covi, S. Spiga, Evidence of soft bound behaviour in analogue memristive devices for neuromorphic computing. *Sci. Rep.* **8**, 7178 (2018).

126. T. Tan, Y. Du, A. Cao, Y. Sun, H. Zhang, G. Zha, Resistive switching of the HfO_x/HfO_2 bilayer heterostructure and its transmission characteristics as a synapse. *RSC Adv.* **8**, 41884–41891 (2018).

127. J. Liu, H. Yang, Y. Ji, Z. Ma, K. Chen, X. Zhang, H. Zhang, Y. Sun, X. Huang, S. Oda, An electronic synaptic device based on HfO_2TiO_x bilayer structure memristor with self-compliance and deep-RESET characteristics. *Nanotechnology* **29**, 415205 (2018).

128. B. Ku, Y. Abbas, S. Kim, A. S. Sokolov, Y. R. Jeon, C. Choi, Improved resistive switching and synaptic characteristics using Ar plasma irradiation on the Ti/HfO2 interface. *J. Alloys Compd.* **797**, 277–283 (2019).

129. C. Giovinazzo, J. Sandrini, E. Shahrabi, O. T. Celik, Y. Leblebici, C. Ricciardi, Analog control of retainable resistance multistates in HfO_2 resistive-switching random access memories (ReRAMs). *ACS Appl. Electron. Mater.* **1**, 900–909 (2019).

130. F. Cüppers, S. Menzel, C. Bengel, A. Hardtdegen, M. von Witzleben, U. Böttger, R. Waser, S. Hoffmann-Eifert, Exploiting the switching dynamics of HfO_2-based ReRAM devices for reliable analog memristive behavior. *APL Mater.* **7**, 091105 (2019).

131. K. C. Chuang, C. Y. Chu, H. X. Zhang, J. D. Luo, W. S. Li, Y. S. Li, H. C. Cheng, Impact of the stacking order of HfO_x and AlO_x dielectric films on RRAM switching mechanisms to behave digital resistive switching and synaptic characteristics. *IEEE J. Electron Devices Soc.* **7**, 589–595 (2019).

132. Q. Chen, G. Liu, W. Xue, J. Shang, S. Gao, X. Yi, Y. Lu, X. Chen, M. Tang, X. Zheng, R.-W. Li, Controlled construction of atomic point contact with 16 quantized conductance states in oxide resistive switching memory. *ACS Appl. Electron. Mater.* **1**, 789–798 (2019).

133. Z. Li, B. Tian, K. H. Xue, B. Wang, M. Xu, H. Lu, H. Sun, X. Miao, Coexistence of digital and analog resistive switching with low operation voltage in oxygen-gradient HfO_x memristors. *IEEE Electron Device Lett.* **40**, 1068–1071 (2019).

134. J. J. Ryu, K. Jeon, S. Yeo, G. Lee, C. Kim, G. H. Kim, Fully "Erase-free" multi-bit operation in HfO_2-based resistive switching device. *ACS Appl. Mater. Interfaces* **11**, 8234–8241 (2019).

135. G. González-Cordero, M. Pedro, J. Martin-Martinez, M. B. González, F. Jiménez-Molinos, F. Campabadal, N. Nafría, J. B. Roldán, Analysis of resistive switching processes in $TiN/Ti/HfO_2/W$ devices to mimic electronic synapses in neuromorphic circuits. *Solid State Electron.* **157**, 25–33 (2019).

136. S. Kim, J. Chen, Y. C. Chen, M. H. Kim, H. Kim, M. W. Kwon, S. Hwang, M. Ismail, Y. Li, X. S. Miao, Y. F. Chang, B. G. Park, Neuronal dynamics in HfO_x/AlO_y-based homeothermic synaptic memristors with low-power and homogeneous resistive switching. *Nanoscale* **11**, 237–245 (2019).

137. C. Liu, C. C. Zhang, Y. Q. Cao, D. Wu, P. Wang, A. D. Li, Optimization of oxygen vacancy concentration in HfO_2/HfO_x bilayer-structured ultrathin memristors by atomic layer deposition and their biological synaptic behavior. *J. Mater. Chem. C* **8**, 12478–12484 (2020).

138. C. Mahata, C. Lee, Y. An, M. H. Kim, S. Bang, C. S. Kim, J. H. Ryu, S. Kim, H. Kim, B. G. Park, Resistive switching and synaptic behaviors of an HfO_2/Al_2O_3 stack on ITO for neuromorphic systems. *J. Alloys Compd.* **826**, 154434 (2020).

139. H. Abbas, Y. Abbas, G. Hassan, A. S. Sokolov, Y. R. Jeon, B. Ku, C. J. Kang, C. Choi, The coexistence of threshold and memory switching characteristics of ALD HfO_2 memristor synaptic arrays for energy-efficient neuromorphic computing. *Nanoscale* **12**, 14120–14134 (2020).

140. Khan, S. Kim, Comparison of diverse resistive switching characteristics and demonstration of transitions among them in Al-incorporated HfO_2-based resistive switching memory for neuromorphic applications. *RSC Adv.* **10**, 31342–31347 (2020).

141. J. Yang, H. Ryu, S. Kim, Resistive and synaptic properties modulation by electroforming polarity in CMOS-compatible Cu/HfO_2/Si device. *Chaos Solitons Fractals* **145**, 110783 (2021).

142. A. A. Koroleva, M. G. Kozodaev, Y. Y. Lebedinskii, A. M. Markeev, Interface engineering for enhancement of the analog properties of W/WO_{3-x}/HfO_2/Pd resistance switched structures. *J. Phys. D Appl. Phys.* **54**, 504004 (2021).

143. H. Algadi, C. Mahata, T. Alsuwian, M. Ismail, D. Kwon, S. Kim, Gradual resistive switching and synaptic properties of ITO/HfAlO/ITO device embedded with Pt nanoparticles. *Mater. Lett.* **298**, 130011 (2021).

144. H. Kang, J. Park, D. Lee, H. W. Kim, S. Jin, M. Ahn, J. Woo, Two- and three-terminal HfO2-based multilevel resistive memories for neuromorphic analog synaptic elements. *Neuromorphic Comput. Eng.* **1**, 021001 (2021).

145. C. Mahata, S. Kim, Modified resistive switching performance by increasing Al concentration in HfO_2 on transparent indium tin oxide electrode. *Ceram. Int.* **47**, 1199–1207 (2021).

146. M. Qi, T. Fu, H. Yang, Y. Tao, C. Li, X. Xiu, Reliable analog resistive switching behaviors achieved using memristive devices in AlO_X/HfO_x bilayer structure for neuromorphic systems. *Semicond. Sci. Technol.* **37**, 035018 (2022).

147. M. Ismail, C. Mahata, S. Kim, Forming-free Pt/Al_2O_3/HfO_2/$HfAlO_x$/TiN memristor with controllable multilevel resistive switching and neuromorphic characteristics for artificial synapse. *J. Alloys Compd.* **892**, 162141 (2022).

148. T. Stecconi, R. Guido, L. Berchialla, A. La Porta, J. Weiss, Y. Popoff, M. Halter, M. Sousa, F. Horst, D. Dávila, U. Drechsler, R. Dittmann, B. J. Offrein, V. Bragaglia, Filamentary TaO_x/HfO_2 ReRAM devices for neural networks training with analog in-memory computing. *Adv. Electron. Mater.* **8**, 2200448 (2022).

149. P. Pal, K. J. Lee, S. Thunder, S. De, P. T. Huang, T. Kämpfe, Y. H. Wang, Bending resistant multibit memristor for flexible precision inference engine application. *IEEE Trans. Electron Devices* **69**, 4737–4743 (2022).

150. M. Ismail, U. Chand, C. Mahata, J. Nebhen, S. Kim, Demonstration of synaptic and resistive switching characteristics in W/TiO_2/HfO_2/TaN memristor crossbar array for bioinspired neuromorphic computing. *J. Mater. Sci. Technol.* **96**, 94–102 (2022).

151. H. Lee, D. G. Ryu, G. Lee, M. K. Song, H. Moon, J. Lee, J. Ryu, J. H. Kang, J. Suh, S. Kim, J. Lim, D. Jeon, S. Kim, J. Kim, Y. S. Lee, Vertical metal-oxide electrochemical memory for high-density synaptic array based high-performance neuromorphic computing. *Adv. Electron. Mater.* **8**, 2200378 (2022).

152. F. Wu, C. H. Chou, T. Y. Tseng, CMOS-compatible memristor for optoelectronic neuromorphic computing. *Nanoscale Res. Lett.* **17**, 105 (2022).

153. D. Maldonado, A. Cantudo, E. Perez, R. Romero-Zaliz, E. Perez-Bosch Quesada, M. K. Mahadevaiah, F. Jimenez-Molinos, C. Wenger, J. B. Roldan, TiN/Ti/HfO_2/TiN memristive devices for neuromorphic computing: From synaptic plasticity to stochastic resonance. *Front. Neurosci.* **17**, 1271956 (2023).

154. Q. Liu, S. Gao, Y. Li, W. Yue, C. Zhang, H. Kan, G. Shen, HfO_2/WO_3 heterojunction structured memristor for high-density storage and neuromorphic computing. *Adv. Mater. Technol.* **8**, 2201143 (2023).

155. M. Ismail, M. Rasheed, S. Kim, C. Mahata, M. Kang, S. Kim, Unveiling the potential of HfO_2/WS_2 bilayer films: Robust analog switching and synaptic emulation for advanced memory and neuromorphic computing. *ACS Mater. Lett.* **5**, 3080–3092 (2023).

156. J. Kim, Y. Park, J. Lee, E. Lim, J. K. Lee, S. Kim, Impact of HfO_2 dielectric layer placement in $Hf_{0.5}Zr_{0.5}O_2$-based ferroelectric tunnel junctions for neuromorphic applications. *Adv. Mater. Technol.* **9**, 2400050 (2024).

157. D. P. Sahu, K. Park, P. H. Chung, J. Han, T. S. Yoon, Linear and symmetric synaptic weight update characteristics by controlling filament geometry in oxide/suboxide HfO_x bilayer memristive device for neuromorphic computing. *Sci. Rep.* **13**, 9592 (2023).

158. Y. R. Jeon, D. Kim, B. Ku, C. Chung, C. Choi, Synaptic characteristics of atomic layer-deposited ferroelectric lanthanum-doped HfO_2 (La:HfO_2) and TaN-based artificial synapses. *ACS Appl. Mater. Interfaces* **15**, 57359–57368 (2023).

159. T. J. Chang, H. H. Le, C. Y. Li, S. Y. Chu, D. D. Lu, $HfTaO_x$ rectifying layer for HfO_x-based RRAM for high-accuracy neuromorphic computing applications. *ACS Appl. Electron. Mater.* **5**, 2566–2573 (2023).

160. J. Niu, Z. Fang, G. Liu, Z. Zhao, X. Yan, Multilevel state ferroelectric La:HfO_2-based memristors and their implementations in associative learning circuit and face recognition. *Sci. China Mater.* **66**, 1148–1156 (2023).

161. J. Chen, X. Liu, C. Liu, L. Tang, T. Bu, B. Jiang, Y. Qing, Y. Xie, Y. Wang, Y. Shan, R. Li, C. Ye, L. Liao, Reconfigurable Ag/HfO_2/NiO/Pt memristors with stable synchronous synaptic and neuronal functions for renewable homogeneous neuromorphic computing system. *Nano Lett.* **24**, 5371–5378 (2024).

162. J. Y. Zhu, J. J. Liao, J. H. Feng, Y. P. Jiang, X. G. Tang, X. B. Guo, W. H. Li, Z. H. Tang, Y. C. Zhou, Synaptic and resistive switching behaviors of Sm-doped HfO_2 films for bio-inspired neuromorphic calculations. *Int. J. Appl. Ceram. Technol.* **21**, 2498–2509 (2024).

163. D. G. Jeong, E. Park, Y. Jo, E. Yang, G. Noh, D. K. Lee, M. J. Kim, Y. Jeong, H. J. Jang, D. J. Joe, J. Chang, J. Y. Kwak, Grain boundary control for high-reliability HfO_2-based RRAM. *Chaos Solitons Fractals* **183**, 114956 (2024).

164. D. Ju, S. Kim, Implementation of edge computing using $HfAlO_x$-based memristor. *J. Alloys Compd.* **997**, 174804 (2024).

165. Q. Zhu, B. Jiang, J. Lan, Z. Hou, Y. Dong, Z. Wang, X. Feng, M. Shen, H. Yu, K. Chen, J. Li, L. Lin, F. Zhou, Y. Li, Analog $Hf_xZr_{1-x}O_2$ memristors with tunable linearity for implementation in a self-organizing map neural network. *Adv. Electron. Mater.* **10**, 2300508 (2024).

166. E. Lim, D. Ju, J. Lee, Y. Park, M. H. Kim, S. Kim, Artificial neural network classification using Al-Doped HfO_x-based ferroelectric tunneling junction with self-rectifying behaviors. *ACS Mater. Lett.* **6**, 2320–2328 (2024).

167. J. H. Ryu, S. Kim, Artificial synaptic characteristics of TiO_2/HfO_2 memristor with self-rectifying switching for brain-inspired computing. *Chaos Solitons Fractals* **140**, 110236 (2020).

168. A. Younis, D. Chu, X. Lin, J. Lee, S. Li, Bipolar resistive switching in p-type Co_3O_4 nanosheets prepared by electrochemical deposition. *Nanoscale Res. Lett.* **8**, 36 (2013).

169. Z. H. Li, J. C. Li, H. P. Cui, Bending effect on resistive switching behavior of HfO_2/NiO p-n heterojunction. *J. Alloys Compd.* **858**, 158091 (2021).

170. J. Y. Chen, M. C. Wu, Y. H. Ting, W. C. Lee, P. H. Yeh, W. W. Wu, Applications of p-n homojunction ZnO nanowires to one-diode one-memristor RRAM arrays. *Scr. Mater.* **187**, 439–444 (2020).

171. S. Chang Lee, Q. Hu, Y. J. Baek, Y. Jin Choi, C. Jung Kang, H. Ho Lee, T. S. Yoon, Analog and bipolar resistive switching in pn junction of n-type ZnO nanowires on p-type Si substrate. *J. Appl. Phys.* **114**, 064502 (2013).

172. W. Wei, H. Sun, X. Dong, Q. Lu, F. Yang, Y. Zhao, J. Chen, X. Zhang, Y. Li, A neotype self-rectifying Cu_3SnS_4-MoO_3 synaptic memristor for neuromorphic applications. *J. Chem. Eng.* **482**, 148848 (2024).

173. L. Zhao, Z. Lu, F. Zhang, G. Tian, X. Song, Z. Li, K. Huang, Z. Zhang, M. Qin, X. SujuanWu, M. Lu, X. Zeng, J. Gao, J. M. Dai, Current rectifying and resistive switching in high density $BiFeO_3$ nanocapacitor arrays on Nb-$SrTiO_3$ substrates. *Sci. Rep.* **5**, 9680 (2015).

174. S. Shrivastava, S. Pratik, A. S. Lin, T. Y. Tseng, Emulating synaptic and nociceptive behavior via negative photoconductivity of a memristor. *IEEE Trans. Electron Devices* **70**, 3530–3535 (2023).

175. J. C. Li, H. P. Cui, X. Y. Hou, Effect of p-n interface on resistive switching of NiO/CeO_2 thin films. *J. Alloys Compd.* **752**, 247–252 (2018).

176. J. C. Li, B. Chen, Y. Qian, Effect of fatigue fracture on the resistive switching of TiO_2-CuO film/ITO flexible memory device. *Curr. Appl. Phys.* **18**, 953–960 (2018).

177. Y. Sun, D. Wen, F. Sun, Influence of blending ratio on resistive switching effect in donor-acceptor type composite of PCBM and PVK-based memory devices. *Org. Electron.* **65**, 141–149 (2019).

178. S. Sikdar, B. P. Sahu, S. Dhar, Investigation of lithium (Li) doping on the resistive switching property of p-Li:NiO/n-β-Ga_2O_3 thin-film based heterojunction devices. *Appl. Phys. Lett.* **122**, 023501 (2023).

179. S. Kossar, R. Amiruddin, A. Rasool, Investigation on asymmetric resistive switching (RS) characteristics in p-NiO/n-ZnO heterojunctions. *Microelectron. Eng.* **254**, 111669 (2022).

180. X. Li, H. Yu, R. Fang, W. Zhu, L. Wang, L. Zhang, Improved resistive switching characteristics in the p+-Si/ZnO:Al/Ni heterojunction device. *Appl. Phys. A* **129**, 50 (2023).

181. Y. Li, H. Sun, L. Yue, F. Yang, X. Dong, J. Chen, X. Zhang, J. Chen, Y. Zhao, K. Chen, Y. Li, Multicolor fully light-modulated artificial synapse based on P-$MoSe_2/P_xO_y$ heterostructured memristor. *J. Phys. Chem. Lett.* **15**, 8752–8758 (2024).

182. P. Solanki, M. Vala, D. Dhruv, S. V. Bhatt, B. Kataria, Resistive switching behaviour of novel GdMnO3-based heterostructures. *Surf. Interfac.* **35**, 102474 (2022).

183. B. Cheng, J. Zhao, L. Xiao, Q. Cai, R. Guo, Y. Xiao, S. Lei, PMMA interlayer-modulated memory effects by space charge polarization in resistive switching based on CuSCN-nanopyramids/ZnO-nanorods p-n heterojunction. *Sci. Rep.* **5**, 17859 (2015).

184. W. Zhang, M. Gao, X. Lei, C. Zhai, Z. Zhang, Novel memristor with Au/SnSe/ITO structure: First fabrication via a hydrothermal and sputtering approach. *J. Alloys Compd.* **994**, 174742 (2024).

185. H. K. Li, T. P. Chen, S. G. Hu, W. L. Lee, Y. Liu, Q. Zhang, P. S. Lee, X. P. Wang, H. Y. Li, G. Q. Lo, Resistive switching in p-type nickel Oxide/n-Type indium gallium zinc oxide thin film heterojunction structure. *ECS J. Solid State Sci. Technol.* **5**, Q239 (2016).

186. K. M. Kim, S. J. Song, G. H. Kim, J. Y. Seok, M. H. Lee, J. H. Yoon, J. Park, C. S. Hwang, Collective motion of conducting filaments in Pt/n-Type TiO_2/p-type NiO/Pt stacked resistance switching memory. *Adv. Funct. Mater.* **21**, 1587–1592 (2011).

187. X. Li, H. Yu, R. Fang, W. Zhu, L. Wang, L. Zhang, Multilevel and low-power resistive switching based on pn heterojunction memory. *J. Electron. Mater.* **53**, 2162–2167 (2024).

188. X. Chen, H. Zhou, G. Wu, D. Bao, Colossal resistive switching behavior and its physical mechanism of Pt/p-NiO/n-$Mg_{0.6}Zn_{0.4}O$/Pt thin films. *Appl. Phys. A* **104**, 477–481 (2011).

189. W. Song, H. Yu, X. Li, R. Fang, W. Zhu, L. Zhang, Electric-controlled resistive switching and different synaptic behaviors in p^+-Si/n-ZnO heterojunction memristor. *IEEE Trans. Electron Devices* **70**, 1648–1652 (2023).

190. S. P. Madhusudanan, K. Mohanta, S. K. Batabyal, Electrical bistability and memory switching phenomenon in Cu_2FeSnS_4 thin films: Role of p-n junction. *J. Solid State Electrochem.* **23**, 1307–1314 (2019).

191. Y. Liu, Y. Qi, P. Zhou, C. Guan, H. Chen, J. Wang, Z. Ma, T. Zhang, Y. Liu, Mechanisms of resistive switching in $BiFeO_3$ thin films modulated by bottom electrode. *J. Phys. D Appl. Phys.* **51**, 025303 (2018).

192. S. Zhai, J. Gong, Y. Feng, Z. Que, W. Mao, X. He, Y. Xie, X. Li, L. Chu, Multilevel resistive switching in stable all-inorganic *n-i-p* double perovskite memristor. *iScience* **26**, 106461 (2023).

193. S. Ali, J. Bae, C. H. Lee, N. P. Kobayashi, S. Shin, A. Ali, Resistive switching device with highly asymmetric current–voltage characteristics: A solution to backward sneak current in passive crossbar arrays. *Nanotechnology* **29**, 455201 (2018).

194. A. M. Nawar, O. H. Abd-Elkader, A. M. El-Mahalawy, L. Aleya, On resistive switching and dielectric spectroscopy characteristics of topological insulator-based heterojunction for memory applications. *Appl. Phys. A* **130**, 158 (2024).

195. H. So, S. Kim, S. Kim, Self-rectifying NiO_X/WO_X heterojunction synaptic memristor for crossbar architectured reservoir computing system. *J. Alloys Compd.* **1003**, 175644 (2024).

196. H. Lu, X. Yuan, B. Chen, C. Gong, H. Zeng, X. Wei, Self-rectifying resistive switching device based on n-ZnO/p-NiO junction. *J. Sol-Gel Sci. Technol.* **82**, 627–634 (2017).

197. H. So, J. K. Lee, S. Kim, Short-term memory characteristics in n-type-ZnO/p-type-NiO heterojunction synaptic devices for reservoir computing. *Appl. Surf. Sci.* **625**, 157153 (2023).

198. X. J. Liu, X. M. Li, Q. Wang, W. D. Yu, R. Yang, X. Cao, X. D. Gao, L. D. Chen, Improved resistive switching properties in $Ti/TiO_x/La_{0.7}Ca_{0.3}MnO_3/Pt$ stacked structures. *Solid State Commun.* **150**, 137–141 (2010).

199. Y. Vygranenko, K. Wang, A. Nathan, Low leakage p-NiO⁄i-ZnO⁄n-ITO heterostructure ultraviolet sensor. *Appl. Phys. Lett.* **89**, 172105 (2006).
